# Fermented beverages in prehispanic Chile: a comprehensive review of their phytochemistry, traditional medicinal uses, bioactivity, and social aspects

**DOI:** 10.3389/fphar.2024.1505873

**Published:** 2024-11-21

**Authors:** Christina Mitsi, Javier Echeverría

**Affiliations:** Departamento de Ciencias del Ambiente, Facultad de Química y Biología, Universidad de Santiago de Chile, Santiago, Chile

**Keywords:** fermented beverages, chichas, prehispanic Chile, health benefits, indigenous foods, phytochemistry

## Abstract

**Background:**

Archaeological, ethnohistorical, and ethnographic evidence supports the ubiquitous use of fermented beverages in prehispanic Chile. Made from a variety of plant raw materials, these beverages have been of special importance regarding many nutritional, medicinal, social, ritual, and religious aspects.

**Purpose:**

The present review aims to provide a comprehensive review of the interdisciplinary information on traditional Chilean fermented beverages, as well as on the starting materials used for their elaboration.

**Materials and Methods:**

Anthropological and ethnopharmacological data were collected through literature searches based on archaeological evidence, books from chroniclers and explorers, as well as modern ethnographic testimonies. Literature data on the chemistry and bioactivity of the prehispanic fermented beverages and the raw materials used were mainly retrieved from digital databases such as SciFinder^®^, PubMed^®^, and Google Scholar^®^.

**Results and Discussion:**

Thirty seven plant raw materials have been used for the elaboration of traditional fermented beverages in prehispanic Chile. Phytochemical and bioactivity data regarding these beverages are rather limited, but a wide spectrum of chemical compounds, health-promoting and disease-preventative effects is reported for the starting materials.

**Conclusion:**

Despite the lack of scientific evidence on traditional prehispanic fermented beverages of Chile, the plant raw material exhibit promising phytochemical profiles and potential health-promoting and disease-preventative benefits. This review underscores the importance of integrating ethnopharmacological perspectives into modern research endeavors.

## 1 Introduction

The production of fermented beverages is one of the oldest food processing technologies known to humanity. Fermentation has been utilized for thousands of years as a cost-effective method to diversify diet, preserve food surpluses, enrich the protein, fatty acids, and vitamin content of available foodstuff, enhance innocuity and mitigate possible toxic effects of the raw materials, and decrease time and fuel requirements during food processing (Steinkraus, 2018).

Various fermentation processes are carried out by different types of microorganisms, including bacteria, yeast, and mycelial fungi, either naturally occurring or intentionally added to the starting materials. These microorganisms lead to the production of different types of fermented food (Steinkraus, 1997), among which alcoholic beverages are produced by yeast fermentation of a vast variety of raw materials.

Fermented alcoholic beverages have been highly valued as significant dietary components in numerous civilizations due to their extended shelf-life under ambient conditions, thereby contributing to food security and improving food safety. Along with their role in the human diet, there is extensive documentation of ethnohistorical and ethnographic information fomenting the role of alcoholic fermented beverages in traditional medicine, social life, and rituals (Tamang and Kailasapathy, 2010).

Although the diversity of traditional fermented beverages in Asia, Africa, and Europe has been thoroughly described in dozens of review articles and textbooks (Tamang et al., 2020), the information regarding fermented beverages prepared and consumed by ancient South American populations is not as extensive (Cutler and Cardenas, 1947; Jennings and Bowser, 2019; Barghini, 2020; Dozier and Jennings, 2021; Lasso García et al., 2024). It is worth noting that, although the topic of fermented beverages of Latin America was recently and extensively reviewed (Lasso García et al., 2024), this revision offers no information regarding Chile.

In the Andean regions, including Chile, indigenous people have long made alcoholic beverages, commonly called *chichas*, using practically all plant-based raw materials, including fruits, grains, starchy tubers, and even mushrooms (Goldstein et al., 2008; Pardo and Pizarro, 2016).

Ethnographical information regarding prehispanic fermented beverages in the Chilean territory has been thoroughly reviewed by Oriana Pardo and José Luis Pizarro (Pardo and Pizarro, 2005; 2016), unravelling of the ritual, religious and social aspects associated with the elaboration and consumption of fermented beverages in prehispanic Chile. The Spanish colonialization marked a turning point that led to the marginalization of traditional Chilean fermented beverages. The introduction of European plant species such as grape and apple, the gradual decline of indigenous communities and the extirpation campaign of the Catholic church against indigenous religious practices were among the principal reasons for the progressive impairment of the traditional practices regarding fermented beverages in Chile (Lacoste et al., 2015; Pardo and Pizarro, 2020).

These traditional beverages are still produced and consumed today, based on empirical knowledge passed down from generation to generation since the prehispanic times (Pardo and Pizarro, 2016). However, the post-colonization marginalization of the Chilean *chichas* may also be reflected to the current status of scientific knowledge regarding prehispanic fermented beverages. Despite the deep-rooted tradition and social fingerprint of *chicha* production in Chile and the diligent ethnographical research of Pardo and Pizarro (Pardo and Pizarro, 2005; Pardo and Pizarro, 2016), scientific literature essentially lacks any information regarding the chemistry and potential associated health benefits of these fermented beverages, thus limiting our understanding of past local civilizations and national heritage and also hindering the realization of future perspectives.

An ethnopharmacological perspective in the study of traditional prehispanic fermented beverages, i.e., an inter- and multidisciplinary perspective that will combine the existing knowledge and new research of diverse areas as anthropology, pharmacology, natural product chemistry, toxicology, etc. is considered more than essential in order to surpass present limitations, rescue traditional knowledge and benefit from it. Moreover, although there is no information regarding the chemistry of traditional *chichas*, it can be expected that the chemical compounds -or some of them-present in the plant raw materials would also be present in the beverages, with or without fermentation-induced transformations. Therefore, the existing scientific knowledge on the chemistry and bioactivity of the plant raw materials, combined or contrasted to traditional medical uses, could possibly mitigate the effects of the lack of respective data on the fermented beverages *per se* and facilitate the orientation of future research on potential health benefits of the traditional Chilean *chichas*.

In this regard, the present review aims to provide a comprehensive review of the interdisciplinary information on traditional Chilean fermented beverages, as well as of the currently available scientific data on the phytochemistry and bioactivity of the starting materials used for the elaboration of traditional Chilean *chichas.*

## 2 Methodology

Literature data on the chemistry and bioactivity of the prehispanic fermented beverages and the raw materials used in Chile for their elaboration were mainly retrieved from digital databases such as SciFinder^®^, PubMed^®^, and Google Scholar^®^, as well as from the scientific journal publishers’ platforms linked with these databases. The search strategy included the scientific name of each raw material without any other keywords or restrictions. All publications in peer-reviewed journals until March 2023 were considered. Since the present review refers to prehispanic fermented beverages of the Chilean territory, chemistry, traditional uses, and bioactivity data regarding species of widespread use in the Andean region (potato (*Solanum tuberosum* L. [Solanaceae]), quinoa (*Chenopodium quinoa* Willd. [Chenopodiaceae]) and maize (*Zea mays* L. [Poaceae]), as well as European species introduced in Chile during the Spanish colony (see Section 3) were not included in the literature review.

Literature data regarding yeast diversity in traditional fermented beverages (see Section 4.1) was retrieved using the same databases and platforms and the same timeframe, applying the keywords “fermented beverage”, “chicha”, “yeast” and restricting the retrieved results to traditional alcoholic fermentation products in South America.

Considering ethnohistorical, ethnographical, anthropological and ethnopharmacological data included in Sections 3, 4, 6, and 7, literature search was based on archaeological evidence, books from Chilean and non-Chilean chroniclers and explorers, as well as modern ethnographic testimonies regarding traditional practices in the past and fermentation traditions that persist in some communities in Chilean territory.

Plant nomenclature has been checked with the Kew Royal Botanic Gardens’ Medicinal Plant Names Services (MPNS, 2021) and The World Flora Online Database (WFO, 2022). Regarding phytochemical data, only secondary metabolites present in the raw materials were considered and they were classified according to their pathway and superclass (Supplementary Table S1) using the NPClassifier tool (Kim et al., 2021), except anthocyanins that are presented as a separate category and not included in the flavonoids superclass, due to their high occurrence in the raw materials. A matrix plot (Figure 2) was employed to visually summarize the occurrence of each phytochemicals’ category per plant raw material. Traditional uses and evidence-based bioactivity data (Supplementary Tables S2, S3) were grouped based on their therapeutic classification according to the Anatomical Therapeutic Chemical (ATC) system, introduced by the World Health Organization (WHO Collaborating Centre for Drug Statistics Methodology, 2023). In the cases of ATC systems for which both traditional and evidence-based bioactivity was reported, a chord diagram was employed to permit the visualization of the relationships between the botanical species and the type of available information (Figure 3). Although not included in the aforementioned systems, evidence-based antioxidant activity was also considered due to its significance and the high number of related research papers.

## 3 Geographical and botanical diversity of traditional fermented beverages

A wide range of raw materials, including cereals, pseudo-cereals, fruits, and various other plant-derived components, were used for the production of fermented beverages in prehispanic Chile. Specifically, a total of thirty eight starting materials have been reported, including thirty seven plant materials and the fungi of the genus *Cyttaria* [Cyttariaceae] (Table 1). Plant materials used for the elaboration of prehispanic *chichas* include species distributed in twenty seven genera and twenty botanical families. The majority of plants belonged to the Fabaceae (*n* = 5), Anacardiaceae (*n* = 4), and Myrtaceae (*n* = 4) botanical families. The recorded species occur as various life forms with the majority being trees (43%) and the remaining occurring as shrubs (30%), herbaceous species (24%),and geophytes (3%).

**TABLE 1:** Raw materials used for the elaboration of prehispanic fermented beverages in Chile.

Species - family	Vernacular name(s)^a^	Part used in fermentation	Geographical range of fermented beverage in Chile	Geographical distribution of raw materials^a^	References
*Alstroemeria ligtu* L. [Alstroemeriaceae]	Liuto, ligtu	Rhizomes	Center and South	VAL, RME, LBO, MAU, NUB, BIO, ARA	Maccioni (2008); Pardo and Pizarro (2013); Pardo and Pizarro (2016)
*Amomyrtus luma* (Molina) D.Legrand & Kausel [Myrtaceae]	Luma, cauchahue	Fruits	South	MAU, NUB, BIO, ARA, LRI, LLA, AIS	de Moraleda (1888); Lenz (1910); Muñoz S. et al. (1981); Molina (1987); de Mösbach (1992); Rapoport et al. (2003); Cárdenas Álvarez and Villagrán (2005); Pardo and Pizarro (2013); Pardo and Pizarro (2016); Jara Vergara et al. (2014); Cárdenas Álvarez and Muñoz Arias (2015)
*Araucaria araucana* (Molina) K.Koch (syn. *Araucaria imbricata* Pav.) [Araucariaceae]	Araucaria, piñonero, pehuén	Seeds	South	BIO, ARA, LRI	Muñoz et al. (1981); Rapoport et al. (2003); Pardo and Pizarro (2013); Pardo and Pizarro (2016)
*Aristotelia chilensis* (Molina) Stuntz [Elaeocarpaceae]	Maqui, clon	Fruits	Center and South	COQ, VAL, RME, LBO, MAU, NUB, BIO, ARA, LRI, LLA, AIS, JFE	Gay (1865); Murillo (1889); Lenz (1910); Pöppig (1960); Muñoz et al., (1981); Molina, (1987); de Mösbach, (1992); Hoffmann et al., (1992); Rapoport et al. (2003); Cárdenas Álvarez and Villagrán, (2005); Pardo and Pizarro, (2016); Pardo and Pizarro (2013); Cárdenas Álvarez and Muñoz Arias (2015)
*Baccharis* spp. [Asteraceae]	Chilca	-	South	-	Pöppig (1960)
*Berberis* spp. [Berberidaceae]	Calafate, michay	Fruits	South	-	Lenz (1910); Pöppig (1960); Rapoport et al. (2003); Pardo and Pizarro (2013); Pardo and Pizarro (2016)
*Berberis darwinii* Hook. [Berberidaceae]	Michay	Fruits	South	RME, MAU, NUB, BIO, ARA, LRI, LLA, AIS	de Mösbach (1992); Cárdenas Álvarez and Muñoz Arias (2015)
*Berberis microphylla* G. Forst. (syn*. Berberis buxifolia* Lam., *Berberis parodii* Job) [Berberidaceae]	Calafate, michay, mulun	Fruits	South	RME, LBO, MAU, NUB, BIO, ARA, LRI, LLA, AIS, MAG	Rapoport et al. (2003); Cárdenas Álvarez and Villagrán (2005); Cárdenas Álvarez and Muñoz Arias (2015)
*Bromus mango* É.Desv. [Poaceae]	Mango, mangu	Grains	South	RME, ARA	Gay (1865); Cárdenas Álvarez and Villagrán, (2005); Pardo and Pizarro (2013); Pardo and Pizarro (2016); Cárdenas Álvarez and Muñoz Arias (2015)
*Chenopodium pallidicaule* Aellen [Amaranthaceae]	Qañiwa, cañihua, cañahua	Grains	-	-	Cobo et al. (1964); Tapia (2000); Goldstein et al. (2008); Pardo and Pizarro (2013); Pardo and Pizarro (2016)
*Chenopodium quinoa* Willd. [Amaranthaceae]	Quínoa, quingua, quinua, chula, ch’iva, ch’ivaqhora	Grains	North, Center, and South	TAR, ANT, NUB, LLA	de Moraleda (1888); Lenz (1910); Pöppig (1960); Cobo et al. (1964); Molina (1987); Tapia (2000); Goldstein et al. (2008); Pardo and Pizarro (2013); Pardo and Pizarro (2016); Cárdenas Álvarez and Muñoz Arias (2015)
*Cyttaria* spp. [Cyttariaceae]	Dihueñe, pinatra, llaullau	Fungal body	South	-	Lenz (1910); de Mösbach (1992); Cárdenas Álvarez and Villagrán (2005); Pardo and Pizarro (2013); Pardo and Pizarro (2016)
*Ephedra ochreata* Miers [Ephedraceae]	Solupe, camán	Fruits	South	AIS	Rapoport et al. (2003)
*Fragaria chiloensis* (L.) Mill (syn. *Potentilla chiloensis* (L.) Mabb.) [Rosaceae]	Frutilla, lahueñe	Fruits	Center and South	LBO, MAU, NUB, BIO, ARA, LRI, LLA, AIS, MAG, JFE	Cobo et al. (1964); de Vivar (1966); de Mösbach (1992); Rapoport et al. (2003); Cárdenas Álvarez and Villagrán (2005); Pardo and Pizarro (2013); Pardo and Pizarro (2016); Cárdenas Álvarez and Muñoz Arias (2015)
*Gaultheria mucronata* (L.f.) Hook. & Arn. [Ericaceae]	Chaura	Fruits	Center and South	LRI, LLA, AIS, MAG	de Mösbach (1992); Rapoport et al. (2003); Cárdenas Álvarez and Muñoz Arias (2015)
*Gaultheria poeppigii* DC. (syn. *Pernettya myrtilloides* Zucc. ex Steud.) [Ericaceae]	Chaura	Fruits	Center and South	MAU, NUB, BIO, ARA, LRI, LLA, AIS	de Mösbach (1992); Rapoport et al. (2003)
*Geoffroea decorticans* (Gillies ex Hook. & Arn.) Burkat [Fabaceae]	Chañar	Fruits	North	AYP, TAR, ANT, ATA, COQ	de Vivar (1966); Muñoz et al. (1981); Rapoport et al. (2003); Pardo and Pizarro (2013); Pardo and Pizarro (2016)
*Greigia sphacelata* (Ruiz & Pav.) Regel [Bromeliaceae]	Chupón	Fruits	South	MAU, NUB, BIO, ARA, LLA	Cárdenas Álvarez and Villagrán (2005); Pardo and Pizarro (2013); Pardo and Pizarro (2016); Cárdenas Álvarez and Muñoz Arias (2015)
*Jubaea chilensis* (Molina) Baill. [Arecaceae]	Palma chilena	Sap	Center	COQ, VAL, RME, LBO, MAU	Murillo (1889); Muñoz et al. (1981); Pardo and Pizarro (2013); Pardo and Pizarro (2016)
*Lithraea caustica* (Molina) Hook. & Arn. [Anacardiaceae]	Litre	Fruits	Center and South	ATA, COQ, VAL, RME, LBO, MAU, NUB, BIO, ARA, LRI	Murillo (1889); Lenz (1910); Muñoz et al. (1981); de Mösbach (1992); Pardo and Pizarro (2013); Pardo and Pizarro (2016)
*Luma apiculata* (DC.) Burret (syn. *Myrceugenella apiculata* (DC.) Kausel) [Myrtaceae]	Arrayán, palo colorado	Fruits	South	COQ, VAL, RME, LBO, MAU, NUB, BIO, ARA, LRI, LLA, AIS	Muñoz S. et al. (1981); Rapoport et al. (2003); Pardo and Pizarro (2013); Pardo and Pizarro (2016); Cárdenas Álvarez and Muñoz Arias (2015)
*Muehlenbeckia hastulata* (Sm.) I.M.Johnst. [Polygonaceae]	Mollaca, quilo, voqui negro	Fruits	South	AYP, TAR, ATA, COQ, VAL, RME, LBO, MAU, NUB, BIO, ARA, LRI, LLA	Murillo (1889); Lenz, (1910); Muñoz et al., (1981); de Mösbach (1992); Rapoport et al. (2003); Pardo and Pizarro (2013); Pardo and Pizarro (2016); Jara Vergara et al. (2014)
*Muehlenbeckia sagittifolia* (Ortega) Meisn. [Polygonaceae]	Mollaca	Fruits	-	-	Lenz (1910)
*Neltuma alba* (Griseb.) C.E.Hughes & G.P.Lewis (syn. *Prosopis alba* Griseb.) [Fabaceae]	Algarrobo blanco	Pods	North	AYP, TAR, ANT, ATA	Muñoz et al. (1981); de Mösbach (1992); Pardo and Pizarro (2013); Pardo and Pizarro (2016)
*Neltuma alpataco* (Phil.) C.E.Hughes & G.P.Lewis (syn. *Prosopis alpataco* Phil.) [Fabaceae]	Alpataco, pichai	Pods	South	-	Rapoport et al. (2003)
*Neltuma chilensis* (Molina) C.E.Hughes & G.P.Lewis (syn. *Prosopis chilensis* (Molina) Stuntz) [Fabaceae]	Algarrobo del centro	Pods	North	TAR, ANT, ATA, COQ, VAL, RME, LBO	Muñoz et al., (1981); Pardo and Pizarro (2013); Pardo and Pizarro (2016)
*Otholobium glandulosum* (L.) J.W.Grimes (syn. *Psoralea glandulosa* L.) [Fabaceae]	Culén	Fresh stems and leaves	South	AYP, TAR, COQ, VAL, RME, LBO, MAU, NUB, BIO, ARA, LRI	Hoffmann et al. (1992); Cárdenas Álvarez and Muñoz Arias (2015)
*Peumus boldus* Molina [Monimiaceae]	Boldo, boldu	Fruits	South	COQ, VAL, RME, LBO, MAU, NUB, BIO, ARA, LRI, LLA	San Martín and Doll8 (1998)8; Pardo and Pizarro (2013); Pardo and Pizarro (2016)
*Prumnopitys andina* (Poepp. ex Endl.) de Laub. (syn. *Podocarpus andinus* Poepp. ex Endl.) [Podocarpaceae]	Lleuque, uva de la cordillera	Fruits	-	MAU, NUB, BIO, ARA	Muñoz et al. (1981); Pardo and Pizarro (2013); Pardo and Pizarro (2016)
*Ribes magellanicum* Poir. [Grossulariaceae]	Zarzaparrilla, parrilla	Fruits	South	COQ, VAL, RME, LBO, MAU, NUB, BIO, ARA	Rapoport et al. (2003); Cárdenas Álvarez and Villagrán (2005); Pardo and Pizarro (2013); Pardo and Pizarro (2016); Cárdenas Álvarez and Muñoz Arias (2015)
*Rubus geoides* Sm. [Rosaceae]	Miñe, frutilla	Fruits	South	BIO, ARA, LRI, LLA, AIS, MAG, JFE	Cárdenas Álvarez and Muñoz Arias (2015); Pardo and Pizarro (2016)
*Schinus latifolia* (Gillies ex Lindl.) Engl. (syn. *Lithraea molle* Gay) [Anacardiaceae]	Molle	Fruits	Center	COQ, VAL, RME, LBO, MAU	Murillo (1889); Muñoz et al. (1981); de Mösbach (1992); Pardo and Pizarro (2013); Pardo and Pizarro (2016)
*Schinus molle* L. [Anacardiaceae]	Molle, pimiento, pimentero	Fruits	North and Center	-	Gay (1865); Lenz (1910); Pöppig (1960); Cobo et al. (1964); Molina (1987); de Mösbach (1992); Goldstein et al., (2008); Pardo and Pizarro (2013); Pardo and Pizarro (2016)
*Schinus polygama* (Cav.) Cabrera (syn. *Duvaua dependens* DC.) [Anacardiaceae]	Huingán	Fruits	Center	ATA, COQ, VAL, RME, LBO, MAU, NUB, BIO, ARA, LRI, LLA	Gay (1865); Murillo (1889); Lenz (1910); Muñoz et al. (1981); Molina (1987); de Mösbach (1992); Pardo and Pizarro (2013); Pardo and Pizarro (2016)
*Solanum tuberosum* L. [Solanaceae]	Papa, poñi	Tubers	North, Center, and South	ANT, VAL, RME, LBO, BIO, ARA, LLA, AIS	de Moraleda, (1888); Goldstein et al. (2008); Pardo and Pizarro, (2013); Pardo and Pizarro (2016); Cárdenas Álvarez and Muñoz Arias (2015)
*Ugni molinae* Turcz. (syn. *Ugni philippii* O.Berg, *Ugni poeppigii* O.Berg) [Myrtaceae]	Murtilla, murta, murtillo, uñi	Fruits	South	LBO, MAU, NUB, BIO, ARA, LRI, LLA, AIS, JFE	Gay (1865); Lenz, 1910; Muñoz et al., 1981; Molina, 1987; de Mösbach, 1992; Rapoport et al., 2003; Cárdenas Álvarez and Villagrán, 2005; Pardo and Pizarro, 2013; 2016; Cárdenas Álvarez and Muñoz Arias, 2015)
*Ugni selkirkii* (Hook. & Arn.) O.Berg (syn. *Ugni berteroi* (Phil.) F.Phil.) [Myrtaceae]	Murtillo	Fruits	-	JFE	de Mösbach (1992)
*Zea mays* L. [Poaceae]	Uhua, hua, maíz	Grains	North, Center, and South	IPA	Gay (1865); Lenz (1910); Cobo et al. (1964); de Vivar (1966); Molina (1987); Goldstein et al. (2008); Pardo and Pizarro (2013); Pardo and Pizarro (2016); Cárdenas Álvarez and Muñoz Arias (2015)

^a^ (Rodriguez et al., 2018). Distribution abbreviations: AYP: arica y parinacota, TAR: Tarapacá, ANT: antofagasta, ATA: atacama, COQ: coquimbo, VAL: Valparaíso, RME: metropolitana de santiago, LBO: Libertador Bernardo O’ higgins, MAU: maule, NUB: Ñuble, BIO: Biobío, ARA: Araucanía, LRI: Los Ríos, LLA: los lagos, AIS: Aisén, MAG: magallanes, JFE: Juan Fernández; IPA: Isla de Pascua (Easter Island).

The majority of the plant species used in prehispanic fermented beverages were wild harvested from mountain forests, wetlands, shrublands, and wastelands, while only a few of them were cultivated, i.e., *Chenopodium pallidicaule* Aellen [Chenopodiaceae], *C. quinoa* (Delatorre-Herrera, 2003), *S. tuberosum* (Spooner et al., 2012), and *Z. mays* (Ugalde et al., 2021). Among them, the elaboration of fermented beverages from potato (*S. tuberosum*) tubers, as well as grains of quinoa (*C. quinoa* and maize (*Z. mays* L. [Poaceae]) was not characteristic of the Chilean territory but was and remains widespread in the Andean region.

Furthermore, although out of the scope of the present review, it should be highlighted that since the Spanish colony, grains and fruits of introduced species such as European apple (*Malus domestica* Suckow Borkh. [Rosaceae]), European pear (*Pyrus communis* L. [Rosaceae]), blackberry (*Rubus ulmifolius* Schott [Rosaceae]), quince (*Cydonia oblonga* Mill. [Rosaceae]), linseed (*Linum* spp. [Linaceae]), wheat (*Triticum* spp. [Poaceae]), and barley (*Hordeum vulgare* L. [Poaceae]) were used to make fermented drinks (Gay, 1865; de Moraleda, 1888; Cárdenas Álvarez and Villagrán, 2005; Pardo and Pizarro, 2005; Pardo and Pizarro, 2013; Pardo and Pizarro, 2016).

**FIGURE 1:**
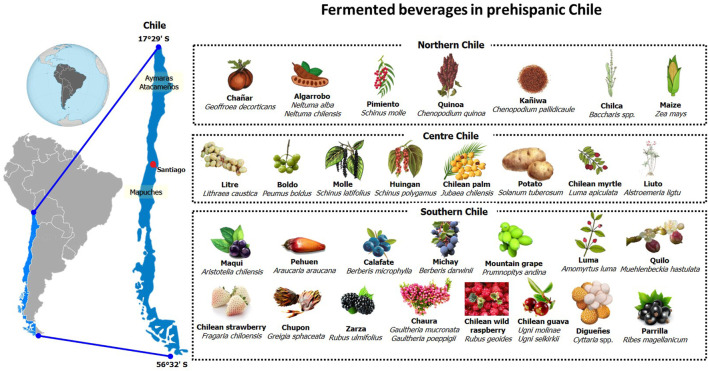
Geographical and botanical diversity of traditional fermented beverages in prehispanic Chile.

Regarding the geographical distribution of plant raw materials and, hence, the range of the respective fermented beverages, those can be divided in three zones, i.e., North (Regions of Arica and Parinacota, Tarapacá, Antofagasta, Atacama, and Coquimbo), Center (Metropolitan Region of Santiago and Regions of Valparaíso, Libertador Bernardo O’ Higgins, Maule, and Ñuble), and South (Regions of Biobío, Araucanía, Los Ríos, Los Lagos, Aisén, and Magallanes). Among those zones, the variety of raw materials used for the elaboration of fermented beverages was limited to the use of cereal grains and legume pods and fruits in dry regions in Northern Chile, while the climatic conditions in the central and southern zones of Chile permitted the use of a much wider variety of plant species and parts (Table 1; Figure 1).

### 3.1 Northern Chile

In Northern Chile, despite the hostile conditions, small Aymara and Atacameño farming and herding settlements existed in the region for several thousand years (Aldunate et al., 1983). They built elaborate trade networks that extended to the coast and the edge of the Amazon; however, just like plants, survival there has developed in a unique way. In the hyperarid Atacama Desert, although native plant resources are scarce, there is a high variety and diversity of food plant species used from ancient to current indigenous populations. The sweet pods of algarrobo trees (*Neltuma* spp. [Fabaceae]), and fruits of chañar (*Geoffroea decorticans* (Gillies ex Hook. & Arn.) Burkart [Fabaecae]) and molle (*Schinus molle* L. [Anacardiaceae]) were key components of the economy and social life, important domestic foods and were consumed as *chicha* during special social activities of the Atacameño people. There is abundant archaeological evidence of the preparation of fermented drinks based on algarrobo, chañar, and molle fruits throughout the prehistory of the Atacama desert (García et al., 2020; Schmeda-Hirschmann et al., 2020; Ugalde et al., 2021; Nuñez and Echeverría, 2024). The maize (*Z. mays*) *chicha*, and to a lesser extent quinoa (*C. quinoa*) *chicha*, were introduced and consumed during the Inka period (Vinton et al., 2009; Wilson et al., 2013; Arriaza et al., 2015). Due to the cultivation of these species, their use for the production of fermented beverages expanded to all Chilean territory.

### 3.2 Central Chile

The central zone of Chile is characterized by a Mediterranean-type climate with hot dry summers and cold rainy winters (Armesto and Martinez, 1978), while the vegetation of the area is characterized by a spatially heterogeneous mosaic of different types of dry forest, including some xerophytes, savanna-type shrubland and evergreen sclerophyllous forest (Gajardo, 1994). Fruits of various tree species such as *Aristotelia chilensis* (Molina) Stuntz [Elaeocarpaceae], *Lithraea caustica* (Molina) Hook. & Arn. [Anacardiaceae], *Schinus latifolia* (Gillies ex Lindl.) Engl. [Anacardiaceae], *S. molle*, and *Schinus polygama* (Cav.) Cabrera [Anacardiaceae] were used by inhabitants for the preparation of fermented beverages (Table 1). Additionally, the sap extracted from the trunk of the Chilean palm *Jubaea chilensis* (Molina) Baill. [Arecaceae] and the rhizomes of *Alstroemeria ligtu* L. [Alstroemeriaceae] were also exploited for this purpose.

### 3.3 Southern Chile

The Patagonian and Valdivian temperate forests are the most predominant ecoregions in southern Chile (Tecklin et al., 2011). Indigenous people used to prepare fermented beverages using various types of berries or berry-like fruits growing in these ecoregions, including *Amomyrtus luma* (Molina) D. Legrand & Kausel [Myrtaceae], *Berberis darwinii* Hook. and *B. microphylla* G. Forst. [Berberidaceae], *Ephedra ochreata* Miers [Ephedraceae], *Fragaria chiloensis* (L.) Mill [Rosaceae], *Gaultheria mucronata* (L.f.) Hook. & Arn., and *G. poeppigii* DC. [Ericaceae], *Greigia sphacelata* (Ruiz & Pav.) Regel [Bromeliaceae], *Luma apiculata* (DC.) Burret [Myrtaceae], *Muehlenbeckia hastulata* (Sm.) I.M.Johnst [Polygonaceae], *Peumus boldus* Molina [Monimiaceae], *Ribes magellanicum* Poir. [Grossulariaceae], *Rubus geoides* Sm. [Rosaceae], and *Ugni molinae* Turcz [Myrtaceae] (Schmeda-Hirschmann et al., 2019). Although fruit-derived fermented beverages were the most common in the Chilean South, during prehispanic times *chichas* were also prepared using fresh aerial parts of culén (*Otholobium glandulosum* (L.) J.W.Grimes [Fabaceae]) and potato (*S. tuberosum*) tubers.

Furthermore, through the cooking and fermentation of the seeds of *Araucaria araucana* (Molina) K. Koch [Araucariaceae], known as *piñones*, the highly valued alcoholic beverage called *muday* is obtained (Herrmann, 2006). There is archaeological evidence regarding the fermentation of *A. araucana* seeds in Patagonian Andean Forests (Saghessi et al., 2024), while the production and consumption continue up to day and *muday* is widely used in ceremonies of the Mapuche people (Herrmann, 2006).

Finally, indigenous peoples of the Chilean South, including Huilliches and Mapuches, gathered the fruiting bodies of *Cyttaria* spp., an ascomycete fungus that grows parasitically on *Nothofagus* Blume species in the temperate rainforests of the central-southern regions of Argentina and Chile (Minter et al., 1987). These were used as a food source and also to make *chicha* (de Mösbach, 1992).

## 4 Traditional fermentation processes and conditions in prehispanic Chile

### 4.1 Yeast strains associated with traditional fermented beverages

Yeasts belonging to the genus *Saccharomyces*, and in particular the species *Saccharomyces cerevisiae*, dominate alcoholic fermentation worldwide. Several recent studies have investigated the microbiological characterization of Latin American traditional *chichas*, e.g., Brazil (Puerari et al., 2015a; Puerari et al., 2015b; Resende et al., 2018; Chacón-Mayorga et al., 2021), Ecuador (Piló et al., 2018; Grijalva-Vallejos et al., 2020; Grijalva-Vallejos et al., 2021), Colombia (Delgado-Ospina et al., 2022), México (Gutiérrez-Sarmiento et al., 2022). In the bordering areas of the Chilean territory, such as northern Argentina and Peru, maize-based *chichas* are primarily made using the yeast *S. cerevisiae* (Vallejo et al., 2013; Mendoza et al., 2017). In Andean Patagonia (Argentina-Chile), the fermentation of apple *chicha* has been attributed to *Saccharomyces uvarum* (Rodríguez et al., 2016), while *S. uvarum* and *Saccharomyces eubayanus* are associated with spontaneous fermentations of *Araucaria araucana* seeds for the elaboration of *muday* (Rodríguez et al., 2014). However, to date, the scientific literature does not provide information regarding the yeast species used in the past and/or currently for the production of fermented beverages from northern and central Chile, nor for the fermentation of plant raw materials other than araucaria seeds in the Chilean South (Table 1). Most importantly, microbial process are associated to human nutrition, but also to cultural heritage and traditional medicinal knowledge (Nabhan et al., 2010). Thus, the lack of any ethnomicrobiological information regarding traditional fermented beverages in prehispanic Chile poses a threat to the associated indigenous knowledge and highlights the need to further investigate Chilean *chichas* under an holistic perspective which will include ethnomicrobiology.

### 4.2 Fermentation and storage conditions

*Chichas* were traditionally produced using a great variety of procedures, utensils, and local traditions. The elaboration practices reported in prehispanic Chile have been previously reviewed by Pardo and Pizarro (2016). In general, the differences in the preparation procedures can be mainly attributed to the raw material used for the production of the fermented beverage, i.e., whether the starting material was starch-rich or not. In the case of plant materials with a high starch content, a pre-treatment was applied consisting of either malting or chewing the raw material. During malting, the starch-rich grains were soaked in water, left to germinate, and then sun-dried to stop germination. The enzymes, mainly amylases, produced during malting or contained in human saliva and introduced to the raw materials during chewing converted starch to simpler sugars that could be used by yeast during fermentation (Pardo and Pizarro, 2016; Dozier and Jennings, 2021). On the other hand, when berries or other non-starchy plant material were used, no pre-treatment was needed.

The non-starchy starting material, as well as the pre-treated cereals, were mashed or ground, boiled in water, strained, and left to ferment. Although chroniclers report different fermentation practices in prehispanic Chile, traditional fermented beverages were produced after short fermentation processes that lasted from 12 h to 2–4 days (Pardo and Pizarro, 2016), and the resulting *chichas* were typically of low alcoholic content and were often consumed well before fermentation was complete (Dozier and Jennings, 2021).

Fermentation vessels were exclusively used for the preparation of *chicha*, therefore yeast residues from past production served as starting culture for the new fermentation (Cutler and Cardenas, 1947; Dozier and Jennings, 2021). In prehispanic Chile, *chicha* was fermented and stored mainly in clay vessels, while it is also reported the use of bottle gourds (*Lagenaria siceraria* (Molina) Standl.) in the north and of leather baskets (*tracal*) in the Chilean south (Pardo and Pizarro, 2016).

## 5 Phytochemistry of the Chilean prehispanic fermented beverages and the raw materials used for their elaboration

Scientific literature provides rather limited information on the chemical composition of Chilean traditional fermented beverages. To our knowledge, there is only one study reporting the presence of quercetin, vicenin II, vitexin, and cinnamic acid in beverages of *Neltuma alba* (Griseb.) C.E.Hughes & G.P.Lewis [Fabaceae] pods after spontaneous fermentation (Rodríguez et al., 2020).

Furthermore, there is a lack of scientific information regarding the chemical composition of several starting materials used for the elaboration of prehispanic fermented beverages, i.e., the rhizomes of *A. ligtu*; the fruits of *A. luma*, *E. ochreata*, *L. caustica*, *M. hastulata*, *M. sagittifolia* (Ortega) Meisn. [Polygonaceae], *S. latifolia*, *Ugni selkirkii* (Hook. & Arn.) O. Berg [Myrtaceae]; the grains of *Bromus mango* É.Desv. [Poaceae]; the pods of *Neltuma alpataco* (Phil.) C.E.Hughes & G.P.Lewis [Fabaceae] and the sap of *J. chilensis*.

**FIGURE 2:**
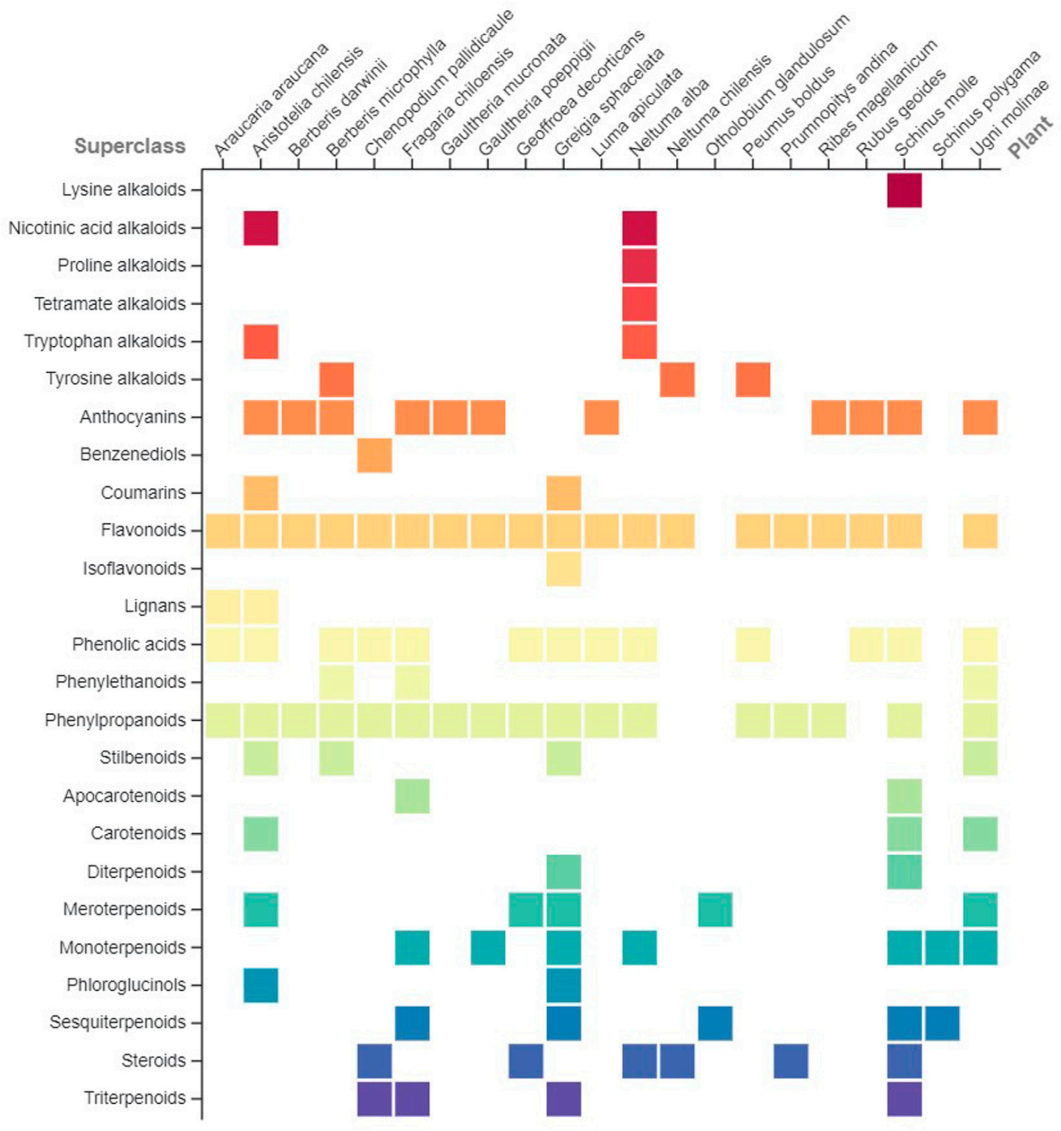
Classification per chemical superclass of the compounds reported in the raw materials used in the elaboration of prehispanic fermented beverages in Chile. Only the chemical composition of the part used in fermentation (Table 1) has been considered.

However, despite the aforementioned limitations, a significant number of compounds have been reported in raw materials used for the preparation of prehispanic *chichas* in Chile, as presented in summary in Figure 2 and in detail in Supplementary Table S1. Considering only the plant parts used during fermentation, the most abundant chemical group reported in plant starting materials corresponds to molecules resulting from the shikimates and phenylpropanoids synthetic pathway, especially flavonoids and phenylpropanoids. Terpenoids have also been reported in several raw materials, while alkaloids are present to a much lesser extent. Although the type of molecules cannot be directly correlated to specific plant parts and/or botanical families, the presence of a wide spectrum of compounds must be highlighted. Considering that many of these chemical groups have been associated with pharmacological activities and benefits for the human health (Yao et al., 2004; Rao and Rao, 2007; Cox-Georgian et al., 2019; Neelam et al., 2020; Rajput et al., 2022), it is considered essential to further study the phytochemistry of both the raw materials and of the beverages resulting by their fermentation.

## 6 Traditional medicinal uses and evidence-based bioactivity and health benefits of the Chilean prehispanic fermented beverages and their raw materials

Several health benefits have been traditionally attributed to prehispanic fermented beverages, being the most widely reported that of a potent diuretic and, consequently, a remedy to prevent and/or treat kidney and bladder stones (Pardo and Pizarro, 2016). While this health benefit has been well documented for all *chichas*, the fermented beverages of several plant raw materials have been correlated with additional medicinal uses such as aperitives, astringents, refrigerants, stomachics, and tonics (Supplementary Table S2). However, scientific evidence only reports the potent *in vitro* antioxidant effect and the inhibition of COX-2 and iNOS protein expression in RAW 264.7 macrophages in the case of fermented *A. chilensis* extracts (Wang et al., 2012) and the *in vitro* antioxidant and anti-lipoperoxidation effects of *N. alba* extracts after spontaneous fermentation (Rodríguez et al., 2020).

Regarding the thirty four plant raw materials used for the preparation of prehispanic *chichas* in Chile, i. e., those presented in Table 1 without considering the widely used quinoa, potato, and maize, 60% is widely known and used in the traditional medicine of Chilean indigenous people (Supplementary Table S2). However, scientific literature lacks information on the bioactivity of several plant raw materials used for the elaboration of prehispanic fermented beverages, i.e., the rhizomes of *A. ligtu*; the fruits of *A. luma*, *E. ochreata*, *L. caustica*, *M. hastulata*, *Muehlenbeckia sagittifolia*, *S. latifolia*, *S. polygama* (Cav.) Cabrera [Anacardiaceae], *U. selkirkii*; the grains of *B. mango*; the pods of *N. alpataco* and the sap of *J. chilensis* (Supplementary Table S3).

**FIGURE 3:**
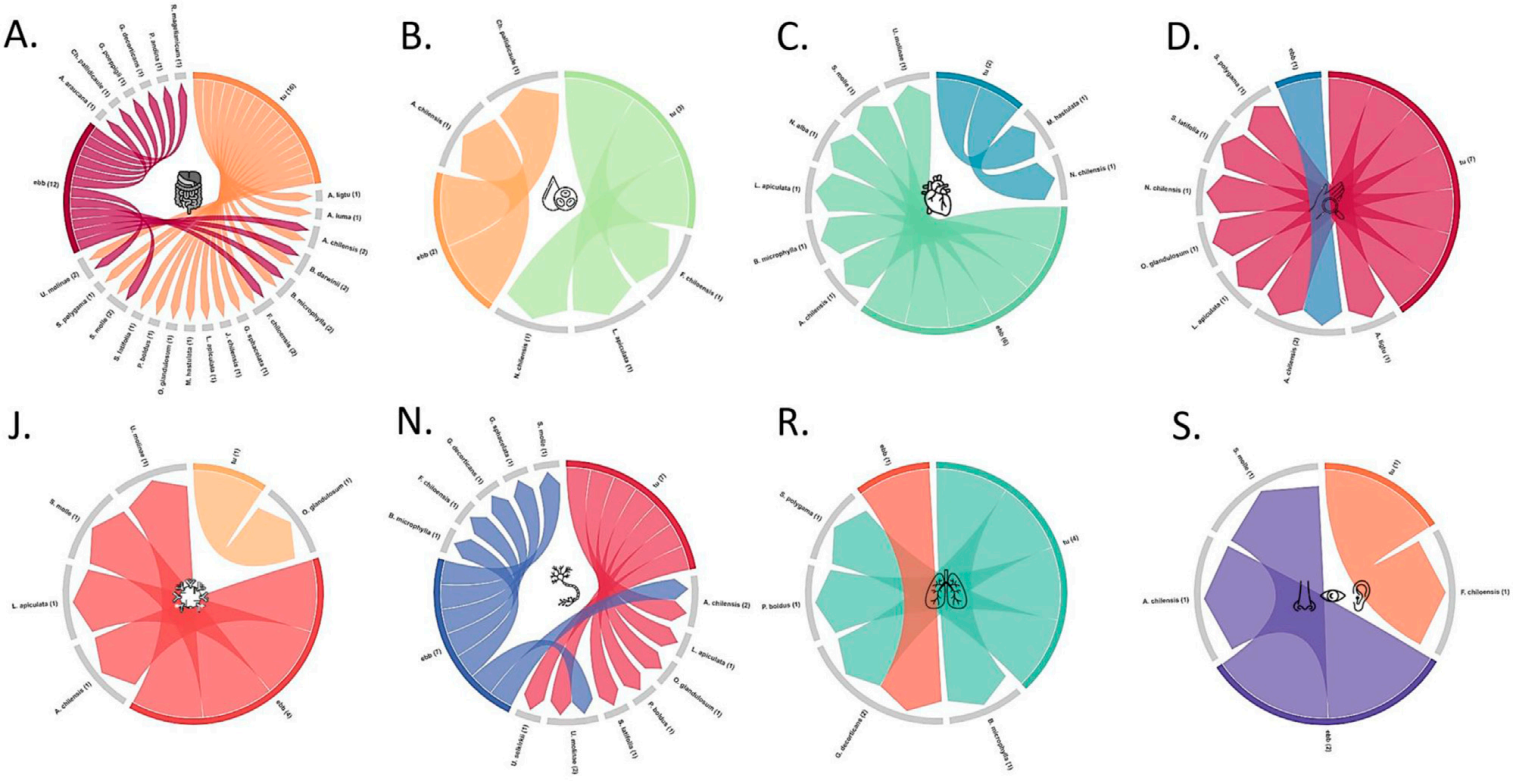
Chord charts correlating the existence of reports on traditional medicinal uses (tu) and evidence-based bioactivity (ebb) regarding raw materials used for the elaboration of prehispanic fermented beverages in Chile, as classified according to the Anatomical Therapeutic Chemical (ATC) system [**(A)**: Alimentary tract and metabolism; **(B)** Blood and blood forming organs; **(C)** Cardiovascular system; **(D)** Dermatologicals; **(J)** Antiinfectives; **(N)**: Nervous system; **(R)**: Respiratory system; **(S)**: Sensory organs]. Numbers in parenthesis indicate correlations between nodes (tu/ebb and plant species).

Conditions related to the digestive tract and to metabolism are those for which there is the highest number of reported traditional uses and evidence-based health benefits (Figure 3A; Supplementary Tables S2, S3). Along with the aforementioned medicinal properties attributed to fermented beverages, a total of sixteen starting materials is associated with traditional uses related to the human alimentary tract, while scientific literature supports similar bioactivity for twelve of the plant raw materials. However, it has to be highlighted that both traditional uses reports and scientific evidence exist for only six plant materials, i.e., the fruits of *A. chilensis*, *B. darwinii*, *B. microphylla*, *F. chiloensis*, *S. molle*, and *U. molinae* (Figure 3A).

Furthermore, there are several traditional uses and scientific evidence related to pathologies of the human nervous system (Figure 3N; Supplementary Tables S2, S3). Seven of the starting materials used for the preparation of prehispanic *chichas* have been used traditionally as tonics, stimulants, and other remedies for the nervous system, while the existing scientific literature supports this type of bioactivity for equal number of plant materials. However, traditional uses and scientific evidence concur only in the case of two raw materials, i.e., the fruits of *A. chilensis*, and *U. molinae* (Figure 3N).

Despite the fact that seven of the examined plant raw materials are traditionally reported as dermatologicals, scientific evidence supports health benefits in the case of skin conditions only for the fruits of *A. chilensis* (Figure 3D; Supplementary Tables S2, S3).

In the case of health benefits related to pathologies of the human respiratory system (Figure 3R; Supplementary Tables S2, S3), although traditional medicines of the indigenous peoples of Chile have associated them with preparations from the fruits of *Berberis microphylla*, *P. boldus*, *S. polygama*, and *G. decorticans*, the existing scientific evidence supports the bioactivity related with the respiratory system only for the latter.

In the case of pathologies related to blood and blood-forming organs, there is no concurrence among the three plant materials with known traditional uses (*F. chiloensis*, *Luma apiculata*, and *Neltuma chilensis*) and those for which there is evidence-based reported bioactivity, i.e., *Aristotelia chilensis* and *C. pallidicaule* (Figure 3B; Supplementary Tables S2, S3). Similarly, there is no concurrence between the two raw materials traditionally used against pathologies of the cardiovascular system and the six reported in scientific literature to present related health benefits (Figure 3C; Supplementary Tables S2, S3). Furthermore, while the aerial parts of *O. glandulosum* is the only plant raw material with documented traditional anti-infective uses, there is scientific evidence supporting the bioactivity of *A. chilensis*, *Luma apiculata*, *chinus molle*, and *U. molinae* fruits (Figure 3J; Supplementary Tables S2, S3). In the case of conditions affecting the sensory organs, only the fruits of *F. chiloensis* present documented traditional uses, wheres scientific evidence is only available for the fruits of *A. chilensis* and *S. molle* (Figure 3S; Supplementary Tables S2, S3).

Interestingly, in the case of pathologies related to the genitourinary system and sex hormones, while there are traditional uses documented for the seeds of *Araucaria araucana* and the fruits of *F. chiloensis*, *P. boldus*, *S. latifolia*, and *S. polygama* (Supplementary Table S2), scientific literature does not provide evidence that supports or refutes these uses. Similarly, while traditional knowledge supports the use of the fruits of *S. polygama* and *P. boldus* as a remedy for musculoskeletal conditions, the existing scientific literature does not provide any information regarding any of the plant raw materials used for the elaboration of prehispanic *chichas* in the Chilean territory (Supplementary Table S2).

Regarding hormone-related bioactivity, there is scientific evidence supporting only the goitrogenic effect of *Araucaria araucana* seed flour (Supplementary Tables S3), while no hormone-related traditional uses are reported for this or any other raw material used for the elaboration of fermented beverages in prehispanic Chile. Similarly, despite the lack of reports on traditional uses, the fruits of *A. chilensis*, *B. microphylla,* and *S. molle* have demonstrated antineoplastic effects (Supplementary Table S3).

Finally, scientific literature supports the antioxidant capacity *in vitro*, in different cell lines and in *vivo* models of many raw materials used for the preparation of prehispanic fermented beverages, namely, the seeds of *Araucaria araucana*; the fruits of *A. chilensis*, *B. darwinii*, *B. microphylla*, *F. chiloensis, G. mucronata*, *G. poeppigii*, *G. decorticans*, *G. sphacelata*, *Luma apiculata*, *Prumnopitys andina* (Poepp. ex Endl.) de Laub. [Podocarpaceae], *R. magellanicum*, *R. geoides*, *S. molle*, and *U. molinae*; the grains of *C. pallidicaule*; the fresh aerial parts of *O. glandulosum* and the pods of *N. alba* and *N. chilensis* (Supplementary Table S3). Particularly in the cases of *A. chilensis* fruits and *N. alba* pods, an interesting antioxidant potential has also been reported for fermented extracts.

In conclusion, it has to be highlighted that, despite the well-documented traditional uses of the prehispanic fermented beverages and of the plant starting materials, the existing scientific literature that supports or refutes these uses is rather limited. Furthermore, in most cases where bioactivity has been investigated, scientific experimentation has not considered traditional uses and knowledge, stressing the need to enhance the ethnopharmacological aspect in modern research so as to unravel the full health-promoting potential of traditional prehispanic fermented beverages and the related plant raw materials.

## 7 Social impact and implications

### 7.1 Role of fermented beverages in prehispanic societies

The chroniclers noted, reproached, and condemned the natives of Chile for their austerity with food but their propensity for consuming *chicha* (de Valdivia, 1553; Gay, 1865; Pérez de García, 1900; Guevara, 1911). However, unlike current context of alcohol consumption, the consumption of *chicha* was more of a social ritual in which participated the entire community, including children, although they consumed less alcoholic preparations (Ovalle, 1646; Núñez de Pineda y Bascuñán, 1973).

In this context, the role of women in *chicha* crafting has been of paramount importance, particularly in Chile. The preparation of fermented beverages by women still stands as a cornerstone culinary tradition among Andean and Mapuche communities (González de Nájera, 1614; Ovalle, 1646; Rosales, 1877). Every step of the process, from the careful selection and gathering of raw materials to the grinding of maize into flour, the provision of water, the actual preparation, and finally, the storage and distribution of *chicha*, was meticulously orchestrated by women (Pardo and Pizarro, 2016). From an early age, young women were apprenticed in the art of *chicha*-making, a tutelage passed down by experienced elders within the community. The preparation of *chicha* epitomized the intergenerational transfer of culinary knowledge, with mature women assuming the role of mentors in guiding the next-generation (Manquilef, 1911).

In prehispanic Chile, fermented beverages held profound significance in the fabric of social life, spanning from everyday communal gatherings to elaborate ritual ceremonies. In the Andes societies, where monetary exchange was absent, reciprocity formed the cornerstone of economic relations (Hastorf and Johannessen, 1993). In this intricate web of exchange, *chicha* emerged as a key element, serving as both a mediator and a facilitator of social interactions. *Chicha* thus played a dual role, symbolizing both the tangible exchange of sustenance and the intangible exchange of goodwill and mutual assistance.

In the Andean region, the introduction of maize during the Inca Empire ushered in profound cultural transformations, particularly in culinary practices. The act of sharing *chicha* became emblematic of social cohesion and hierarchy, fostering its symbolic-religious significance over time. Maize *chicha*, in particular, held immense importance due to its central role in rituals that reinforced power dynamics across various levels of social organization. With the arrival of the Spanish, the Mapuche lacked the centralized state organization of the Inca Empire, and *chicha* did not hold the same centralizing role. However, chiefs demonstrated their status, wealth, and magnificence through the lavish display of *chicha* offerings. The abundance of *chicha* they could provide was often associated with the number of consorts they possessed, symbolizing their perceived power and influence within the community (Rosales, 1877).

Along with its role as a power symbol, *chicha* was deeply integrated into various ceremonies associated with virtually every aspect of communal life in prehispanic Chile. Thus, *chicha* offerings were included in agro-propitiatory and livestock-related ceremonies, expressing gratitude to the natural elements and forces essential for survival and identity formation (Núñez, 1969). In the northern regions, religious events are imbued with profound symbolism, such as the *tinka* (from quechua *t’inka*: gift, tip, reward) or *convido*, which symbolizes both gratitude to Pachamama, the Andean Mother Earth deity, for blessings received and supplication for new benefits (Pardo and Pizarro, 2016). Fermented beverages formed an essential element of the *tinka* rituals, during which *chicha* libations were performed (Grebe and Hidalgo, 1988). Moreover, the cleaning of irrigation canals and *kochas* (*coha or qucha*) is an ancient ceremony that takes place between August and October in numerous oases of the Andean highlands and the Atacama salt flat basin. During this ritual, participants consume algarrobo and maize *chicha* (Grebe and Hidalgo, 1988; Castro and Varela, 1994). The flowering, known as *floreo* or sign of cattle, is a propitiatory ceremony of Northern Chile, aimed at ensuring the health and reproduction of livestock, particularly llamas. This ritual involves marking and adorning the llamas with colorful decorations, and it is conducted in the Andean plateau during both winter and summer solstices, coinciding with the lambing seasons of llamas and sheep respectively (Mostny et al., 1954; Castro, 2009). Throughout the ceremony, participants partake in the consumption of algarrobo and maize *chicha*, reinforcing the connection between humans, animals, and the natural world (Dannemann, 1977). In southern Chile, rituals associated with harvests hold a central place in community life, with threshing festivals (known as *trilla* or *ñiun, ñi’nan, ñiwiñan*, or *ñiwin-ncn*) serving not only practical, but also propitiatory purposes for fertility (Medina, 1882; Lenz, 1897).

Along with agricultural rituals, *chicha* held a special role in social gatherings such as weddings, family meetings (*cahuines*), welcome and farewell parties, where participants were offered *chicha* and food (Ovalle, 1646; Febrés, 1765; Rosales, 1877; de Moraleda, 1888; Guevara, 1911; Manquilef, 1911; Núñez de Pineda y Bascuñán, 1973; Nuñez de Pineda y Basscuñan et al., 1984; Quiroz, 1997). In Mapuche and Atacameño communities, *minga, mink’a,* or *ayni* is a tradition involving the collaborative effort of neighbors and friends in a communal task of an agricultural nature such as planting, weeding, harvesting, building houses (Mostny et al., 1954), constructing or repairing roads, or cleaning irrigation canals, among others. The organizer of the *minga* is expected to provide food and fermented beverages, such as *chicha*, for the participating workers (Núñez de Pineda y Bascuñán, 1973).

Fermented beverages formed an essential part of the religious practices in perhispanic Chile. *Chicha* has always accompanied the ceremonies that mark the life of man, such as births, rituals of passage, deaths, remembrance of the dead (González de Nájera, 1614; Rosales, 1877; Mostny et al., 1954; de Vivar, 1966; Núñez de Pineda y Bascuñán, 1973; Coña, 2006). The consumption of fermented foods and drinks was essential in magical religious festivals to affirm ethnographic supervenience (Núñez, 1969). In Mapuche communities, the *nguillatún* is a ritual meeting ceremony where prayers or requests are made in order to please the *pillan* or the ancestors, offering them dances, food and drink (Encina, 1955). During the *nguillatún*, various ceremonial activities take place, including the consumption and libation of *chicha* made from araucaria seeds and wheat (Joseph, 1930; Gundermann, 1985). Moreover, the *machiluwun* is the ritual ceremony of consecration of a *Machi*, the highest religious authority in a Mapuche community. When the ceremony is announced, the parents and inhabitants of their future jurisdiction store food and prepare the *chicha* that will be distributed. *Chicha* libation is made in glasses especially intended for the ceremony (Joseph, 1930; Métraux, 1985; Coña, 2006).

During armed conflicts and wars, *chicha* played an important role. Women typically remained in the rear to offer *chicha* to soldiers as a refreshment. Among the Mapuche, in times when warfare necessitated expanding the circle of alliances, a supreme chief or war chief (*toqui*) was elected. Upon such an election, a war preparation meeting was convened, attended by initiated warriors. These gatherings were accompanied by lavish feasts featuring ample food and drink, including *chicha* (Rosales, 1877; de Vivar, 1966; Mariño de Lobera, 1970; Ercilla y Zúñiga, 1989). The victories, defeats, and terms of wars gave rise to great celebrations, accompanied by abundant consumption of *chicha*, which were prepared especially for these occasions in memory and honor of the victors (Rosales, 1877; Ercilla y Zúñiga, 1989).

Finally, chicha was also present in sports and playful activities. The *palin* or *uño*, also known as *chueca*, was the Mapuche people’s quintessential sport (Pereira Salas, 1947). Following the chueca games, copious amounts of *chicha* were consumed, often leading to significant intoxication and, in some instances, riots (Rosales, 1877; Pereira Salas, 1947; Coña, 2006). Consequently, governors frequently prohibited such games (Pereira Salas, 1947). Despite the attempts to regulate or suppress such festivities, the *chueca* and the accompanying revelries persisted within Mapuche society (Coña, 2006).

### 7.2 Current status of fermented beverages in Chile

The Spanish colony marked the gradual economical, social and religious marginalization of the traditional fermented beverages of the Chilean territory. Thus, *chichas* derived from native resources, as well as the ethnobotanical and ethnopharmacological heritage associated to their preparation and use became predominantly confined to small indigenous communities in rural areas both in the northern and southern extremities of Chile (Lacoste et al., 2015; Pardo and Pizarro, 2020).

Despite the aforementioned, the production of fermented beverages within small, rural, indigenous groups is still deeply intertwined with tradition and community practices. Fermented beverages are often an integral part of cultural identity, reflecting the unique customs, beliefs, and values of a community. The recipes, techniques, and ingredients used in the production of these beverages are passed down through generations, preserving cultural heritage and reinforcing a sense of belonging and identity among community members. This knowledge encompasses not only the fermentation process itself, but also the selection and preparation of ingredients, the timing of production, and the rituals and ceremonies associated with consumption. Moreover, fermented beverages production often involves collective effort, fostering cooperation and solidarity within the community (Pardo and Pizarro, 2020).

In Chilean territory, many indigenous communities have developed sustainable practices for the production of fermented beverages, utilizing locally sourced ingredients and traditional fermentation techniques that are adapted to their natural environment. This sustainable approach not only preserves biodiversity and traditional cultural and medicinal knowledge, but also strengthens the community’s resilience. In addition to its cultural and social significance, the production of fermented beverages can also have economic implications for indigenous communities. Selling or trading these beverages locally or regionally may provide supplementary income for households, contributing to livelihood diversification and economic resilience (Chavarría and Fuentealba Urzúa, 2018; Montecinos and Alvear, 2018).

## 8 Conclusion

Citing the pioneer Professor Bo Holmstedt (1918–2002) one of the main objectives of Ethnopharmacology is *“to rescue and document an important cultural heritage before it is lost”* (Bruhn and Rivier, 2019). Furthermore, one of the current key topics in ethnopharmacological research is the need to decolonize the field, addressing past misconducts and promoting the protection of traditional knowledge and the fair benefit-sharing as proposed by the Nagoya Protocol (Schultz and Garbe, 2023). Given the historical journey of the traditional fermented beverages of prehispanic Chile, their marginalization and its consequences both on scientific and social level, the need to fullfill both the aforementioned objectives is nowadays imperative.

Still, the incentive for further researching the chemistry and bioactivity of the traditional Chilean *chichas* cannot and should not be limited to safeguarding cultural heritage. With the recent surge in interest surrounding fermented foods and emerging evidence highlighting their health-promoting and disease-preventative benefits, there has been a renewed focus on reviving traditional preparations, especially fermented beverages, for local marketing. These efforts aim to capitalize the growing consumer interest in natural, health-promoting products with cultural significance.

In this context, the results obtained from scientific characterization -chemical, microbiological and pharmacological-can provide valuable input for further advancement in understanding these traditional beverages. Moreover, by leveraging traditional medicinal knowledge, we can better understand the bioactive compounds in these beverages and their health-promoting effects. While currently available literature lacks information on the chemistry and bioactivity of traditional prehispanic Chilean *chichas*, the data reviewed herein regarding the plant raw materials employed reveal a promising potential and stress the need to further investigate these beverages and the traditional knowledge associated to them.
