# Supplementary material for: Fermented beverages in prehispanic Chile: a comprehensive review of their phytochemistry, traditional medicinal uses, bioactivity, and social aspects
Source: Front Pharmacol. 2024 Nov 21;15:1505873. doi: 10.3389/fphar.2024.1505873 (PMC11617176; doi:10.3389/fphar.2024.1505873)
Supplement: Supplementary file 1 [file Table1.DOCX]

**Table S1.** Phytochemistry of the raw materials used in the elaboration of prehispanic fermented beverages in Chile. Only the chemical composition of the part used in fermentation **(Table 1)** has been considered.

| **Species - Family** | **Chemical compounds per superclass** | **References** |
| --- | --- | --- |
| *Araucaria araucana* (Molina) K.Koch (syn. *Araucaria imbricata* Pav.) [Araucariaceae] | **Flavonoids:** amentoflavone; bilobetin; catechin; epicatechin; epigallocatechin; eriodictyol; gallocatechin; ginkgetin; quercetin; taxifolin | (Gallia et al., 2020; Schmeda-Hirschmann et al., 2021) |
|  | **Lignans:** isolariciresinol; lariciresinol; secoisolariciresinol | (Schmeda-Hirschmann et al., 2021) |
|  | **Phenolic acids:** gallic acid; homogentisic acid; piperonylic acid; protocatechuic acid | (Gallia et al., 2020; Schmeda-Hirschmann et al., 2021) |
|  | **Phenylpropanoids:** caffeic acid; 3-*p*-coumaroylquinic acid; 4-*p*-coumaroylquinic acid; ferulic acid; sinapic acid; vanillic acid | (Schmeda-Hirschmann et al., 2021) |
| *Aristotelia chilensis* (Molina) Stuntz [Elaeocarpaceae] | **Anthocyanins:** cyanidin 3-glucoside; cyanidin 3-sambubioside; cyanidin 3,5-diglucoside; cyanidin 3,5-di-(6-acetylglucoside); cyanidin 3-glucoside-5-rhamnoside; cyanidin 3-(2′-acetylrutinoside); cyanidin 3-sambubioside-5-glucoside; cyanidin 3-(3″,6″-dimalonylglucoside); cyanidin 3-(6-malonylglucoside)-7-(6-feruloylglucoside)-3′-glucoside; cyanidin 3-xylosyl (coumaroylglucosyl)galactoside; cyanidin 3-[6-(6-p-coumarylglucosyl)-2-xylosylgalactoside]; delphinidin 3-galactoside; delphinidin 3-glucoside; delphinidin 3-sambubioside; delphinidin 3,5-diglucoside; delphinidin-3,7-diglucoside; delphinidin 3-sambubioside-5-glucoside; delphinidin 3-(2″-galloylgalactoside); delphinidin 3,7-diglucoside-3′,5′-di(6-*p*-coumaroyl-*β*-glucoside); delphinidin 3-(6-feruloyl)-5-diglucoside; malvidin 3-rutinoside; malvidin 3,5-diglucoside; malvidin 3,7-diglucoside; pelargonidin 3-glucoside; pelargonidin 3-rutinoside; pelargonidin 3-sambubioside; pelargonidin 3-sambubioside-5-glucoside; pelargonidin 3-(6″-p-coumaryl sambubioside)-5-(6″′-malonylglucoside); peonidin 3-glucoside-5-(6″-acetylglucoside); peonidin 3-rutinoside; petunidin 3-glucoside; petunidin 3,5-diglucoside | (Diaz et al., 1984; Escribano-Bailón et al., 2006; Ruiz et al., 2010; Schreckinger et al., 2010, 2012; Céspedes et al., 2010a, 2010b, 2017; Gironés-Vilaplana et al., 2012b, 2012a, 2014; Lila et al., 2012; Rojo et al., 2012; Wang et al., 2012; Tanaka et al., 2013; Fredes et al., 2014, 2018a, 2018b; Reyes-Farias et al., 2014, 2016; Genskowsky et al., 2016; Lucas-Gonzalez et al., 2016; Brauch et al., 2016, 2017; Li et al., 2017; Overall et al., 2017; Quispe-Fuentes et al., 2018; Vázquez-Espinosa et al., 2018, 2019; Viuda-Martos et al., 2018; Di Lorenzo et al., 2019; Bastías-Montes et al., 2019, 2022; Sandoval et al., 2019; Zhou et al., 2019; Chen et al., 2020; Agulló et al., 2021b, 2021a; del Campo et al., 2021; Rodríguez et al., 2021; Pineda et al., 2022; Pinto et al., 2022) |
|  | **Carotenoids:** *β*-carotene | (Rodríguez et al., 2016) |
|  | **Coumarins:** brevifolincarboxylic acid; umbelliferone; urolithin M5 | (Zhou et al., 2019; Chen et al., 2020) |
|  | **Flavonoids:** apigenin; apigenin 7-O-glucoside; astilbin; astragalin; avicularine; caryatin; catechin; chrysin; epicatechin; (-)-epigallocatechin gallate; flavan-3-ol; galangin; (-)-gallocatechin gallate; 3-hydroxyflavone; hyperoside; isorhamnetin; isorhamnetin 3-glucoside; kaempferol; kaempferol 3-galactoside; kaempferol 7-glucoside; kaempferol 3-[2″-glucosyl-6″-acetyl-galactoside]7-glucoside; kaempferol 3-(4″;6″-diacetylglucoside)-7-rhamnoside; luteolin 7-glucoside; mangiferin; mangiferin 6'-gallate; myricetin; myricetin 3-galactoside; myricetin 3-glucoside; myricetin 3-glucoside-7-galactoside; narcissin; naringenin; nicotiflorin; pinobanksin; pinocembrin; procyanidin B1; procyanidin B2; quercetin; quercetin 3-glucuronide; quercetin 3-glucoside; quercetin 4′-galactoside; quercetin 5,3′-dimethyl ether; quercetin 3-(6″″-ferulylsophorotrioside); tetramethylquercetin 3-rutinoside; quercitrin; reinutrin; rutin | (Céspedes et al., 2010a; Ruiz et al., 2010, 2016; Céspedes et al., 2010b, 2017; Rubilar et al., 2011; Gironés-Vilaplana et al., 2014, 2012b; Brauch et al., 2016; Lucas-Gonzalez et al., 2016; Rodríguez et al., 2016, 2021; Genskowsky et al., 2016; Li et al., 2017; Nowak et al., 2018; Quispe-Fuentes et al., 2018, 2019; Viuda-Martos et al., 2018; Zhou et al., 2019; Di Lorenzo et al., 2019; Chen et al., 2020; Agulló et al., 2021b; Roldán et al., 2021; Concha-Meyer et al., 2021; Pineda et al., 2022; Pinto et al., 2022) |
|  | **Lignans:** matairesinol | (Chen et al., 2020) |
|  | **Meroterpenoids:** *α*-tocopherol; *γ*-tocopherol | (Rodríguez et al., 2016) |
|  | **Nicotinic acid alkaloids:** nicotinic acid; pyridoxine | (Rodríguez et al., 2016) |
|  | **Phenolic acids:** ellagic acid; ellagic acid 4-O-*β*-D-xylopyranoside; gallic acid; 5-galloylquinic acid; gentistic acid; granatin B; 4-hydroxybenzoic acid; methyl gallate; methyl protocatechuate; protocatechuic acid; protocatechuic acid 4-glucoside; quinic acid; shikimic acid; valoneic acid dilactone | (Céspedes et al., 2010b, 2010a, 2017; Wang et al., 2012; Gironés-Vilaplana et al., 2012b, 2014; Brauch et al., 2016; Lucas-Gonzalez et al., 2016; Rodríguez et al., 2016, 2021; Ruiz et al., 2016; Genskowsky et al., 2016; Li et al., 2017; Nowak et al., 2018; Quispe-Fuentes et al., 2018, 2019; Zhou et al., 2019; Di Lorenzo et al., 2019; Chen et al., 2020; Roldán et al., 2021; Peçanha et al., 2022; Pineda et al., 2022) |
|  | **Phenylpropanoids:** caffeic acid; caftaric acid; chlorogenic acid; *p*-coumaric acid; cryptochlorogenic acid; ferulic acid; *trans*-ferulic acid; neochlorogenic acid; sinapic acid | (Céspedes et al., 2010b, 2010a, 2017; Gironés-Vilaplana et al., 2012b, 2014; Rodríguez et al., 2016, 2021; Genskowsky et al., 2016; Quispe-Fuentes et al., 2019; Nowak et al., 2018; Quispe-Fuentes et al., 2018; Zhou et al., 2019; Roldán et al., 2021; Concha-Meyer et al., 2021; Pineda et al., 2022) |
|  | **Phloroglucinols:** myrciaphenone A; *O*^2^-*β*-glucosidyl-phloroglucinaldehyde | (Li et al., 2017) |
|  | **Stilbenoids:** pterostilbene | (Roldán et al., 2021) |
|  | **Tryptophan alkaloids:** aristoteline; 3-hydroxyindole | (Céspedes et al., 2009, 2010b; Peçanha et al., 2022) |
| *Berberis darwinii* Hook. [Berberidaceae] | **Anthocyanins:** cyanidin 3-glucoside; cyanidin 3-rutinoside; delphinidin 3-glucoside; delphinidin 3-rutinoside; malvidin-3-glucoside; peonidin 3-glucoside; petunidin 3-gentiobioside; petunidin 3-glucoside; petunidin 3-rutinoside | (Medrano et al., 1985; Chamorro et al., 2019) |
|  | **Flavonoids:** epicatechin | (Chamorro et al., 2019) |
|  | **Phenylpropanoids:** chlorogenic acid; 3-*p*-coumaroylquinic acid; 5-*p*-coumaroylquinic acid; neochlorogenic acid | (Chamorro et al., 2019) |
| *Berberis microphylla* G. Forst. (syn*. Berberis buxifolia* Lam*.*, *Berberis parodii* Job ) [Berberidaceae] | **Anthocyanins:** cyanidin 3-glucoside; cyanidin 3-rutinoside; cyanidin 3,7-diglucoside; delphinidin 3-arabinoside; delphinidin 3-galactoside; delphinidin 3-glucoside; delphinidin 3-rutinoside; delphinidin 3;5-diglucoside; delphinidin 3,7-diglucoside; delphinidin 3-rutinoside-5-glucoside; malvidin 3-glucoside; malvidin 3-rutinoside; malvidin 3,7-diglucoside; malvidin 3-rutinoside-5-glucoside; malvidin 3-(6΄΄-acetyl) galactoside; malvidin 3-(6΄΄-coumaroyl) glucoside; peonidin 3-arabinoside; peonidin 3-glucoside; peonidin 3,7-diglucoside; peonidin 3-rutinoside; petunidin 3-galactoside; petunidin 3-glucoside; petunidin 3-rutinoside; petunidin 3,7-diglucoside; petunidin 3-rutinoside-5-glucoside; petunidin 3-(6΄΄-acetyl) glucoside | (Ruiz et al., 2010, 2013b, 2013a, 2014a; Brito et al., 2014; Reyes-Farias et al., 2014; Ramirez et al., 2015, 2021; Bustamante et al., 2018; López et al., 2018; Calfío and Huidobro-Toro, 2019; Chamorro et al., 2019; Boeri et al., 2020; Soto-Covasich et al., 2020; Olivares-Caro et al., 2020; Romero-Román et al., 2021b, 2021a) |
|  | **Flavonoids:** catechin; hyperoside; isorhamnetin; isorhamnetin 3-galactoside; isorhamnetin-3-glucoside; isorhamnetin 3-rutinoside-7-glucoside; kaempferol; kaempferol-3-rutinoside; myricetin; myricetin-3-glucoside; myricetin 3-rutinoside; myricetin 3-rutinoside-7-glucoside; narcissin; 2',3,4,4',6'-pentahydroxychalcone 4'-O-*β*-D-glucoside; quercetin; quercetin-3-glucoside; quercetin 3-rutinoside-7-glucoside; quercitrin; rutin | (Ruiz et al., 2010, 2014b; Brito et al., 2014; Ramirez et al., 2015; Arena et al., 2017; Bustamante et al., 2018; López et al., 2018; Calfío and Huidobro-Toro, 2019; Chamorro et al., 2019; Boeri et al., 2020; Olivares-Caro et al., 2020; Romero-Román et al., 2021b) |
|  | **Phenolic acids:** gallic acid; syringic acid | (Arena et al., 2017; Boeri et al., 2020) |
|  | **Phenylethanoids:** hydroxytyrosol | (Boeri et al., 2020) |
|  | **Phenylpropanoids:** caffeic acid; 3-*trans*-caffeoylglucaric acid; 4-*trans*-caffeoylglucaric acid; chlorogenic acid; *p*-coumaric acid; cryptochlorogenic acid; O-feruloylgalactaric acid; isochlorogenic acid A; isochlorogenic acid C; ferulic acid; 3-feruloylquinic acid; 4-feruloylquinic acid; neochlorogenic acid | (Ruiz et al., 2013b, 2014a; Brito et al., 2014; Ramirez et al., 2015; Arena et al., 2017; Bustamante et al., 2018; López et al., 2018; Chamorro et al., 2019; Boeri et al., 2020; Olivares-Caro et al., 2020) |
|  | **Stilbenoids:** *trans*-resveratrol | (Boeri et al., 2020) |
|  | **Tyrosine alkaloids:** berberine; jatrorrhizine | (Ruiz et al., 2014b; Olivares-Caro et al., 2020) |
| *Chenopodium pallidicaule* Aellen [Amaranthaceae] | **Benzenediols*:** 4-methylresorcinol; resorcinol  *chemical superclass not identified by NPClassifier (Kim et al., 2021a) | (Peñarrieta et al., 2008) |
|  | **Flavonoids:** alcesefoliside; apiorutin; isorhamnetin; isorhamnetin 3-O-robinoside; isorhamnetin 3-O-(2;6-di-O-*α*-rhamnosyl)-*β*-galactopyranoside; isorhamnetin 3-O-*β*-D-apiofuranosyl-(1→2)-O-[*α*-L-rhamnopyranosyl(1→6)]-*β*-D-glucopyranoside; kaempferol; kaempferol 3-O-robinobioside; myricetin; narcissin; quercetin; quercetin 3-O-robinobioside; quercetin 3-O-*β*-D-apiofuranosyl-(1→2)-O-[*α*-L-rhamnopyranosyl(1→6)]-*β*-D-galactopyranoside; rhamnetin; rutin | (Rastrelli et al., 1995; Peñarrieta et al., 2008; Repo-Carrasco-Valencia et al., 2010; Coronado-Olano et al., 2021) |
|  | **Phenolic acids:** catechin; catechin gallate; gallic acid; 4-hydroxybenzoic acid | (Peñarrieta et al., 2008; Repo-Carrasco-Valencia et al., 2010; Coronado-Olano et al., 2021) |
|  | **Phenylpropanoids:** caffeic acid; chlorogenic acid; *p*-coumaric acid; ferulic acid; vanillic acid | (Peñarrieta et al., 2008; Repo-Carrasco-Valencia et al., 2010; Coronado-Olano et al., 2021) |
|  | **Steroids:** 20-hydroxyecdysone; 20,26-dihydroxyecdysone | (Rastrelli et al., 1996b) |
|  | **Triterpenoids:** lucyoside H; momordin II; olean-12-en-28-oic acid, 3-[(O-*β*-D-glucopyranosyl-(1→2)-O-[*β*-D-glucopyranosyl-(1→4)]-*β*-D-glucopyranosyl)oxy]-, *β*-D-glucopyranosyl ester, (3*β*)-; olean-12-ene-28,29-dioic acid, 3-(*β*-D-glucopyranosyloxy)-23-hydroxy-, 28-*β*-D-glucopyranosyl 29-methyl ester, (3*β,*4*α,*20*β*)-; olean-12-ene-28,29-dioic acid, 3-[(O-*β*-D-glucopyranosyl-(1→4)-O-*β*-D-glucopyranosyl-(1→4)-*β*-D-glucopyranosyl)oxy]-23-hydroxy-, 28-*β*-D-glucopyranosyl 29-methyl ester, (3*β,*4*α,*20*β*)-; olean-12-ene-28,29-dioic acid, 3-[(4-O-*β*-D-glucopyranosyl-*β*-D-glucopyranosyl)oxy]-23-hydroxy-, 28-*β*-D-glucopyranosyl 29-methyl ester, (3*β,*4*α,*20*β*)-; *β*-D-glucopyranosiduronic acid, (3*β*,4*α*)-28-(*β*-D-glucopyranosyloxy)-23-hydroxy-28-oxoolean-12-en-3-yl 3-O-*α*-L-arabinopyranosyl- | (Rastrelli et al., 1996a) |
| *Fragaria chiloensis* (L.) Mill (syn. *Potentilla chiloensis* (L.) Mabb.) [Rosaceae] | **Anthocyanins**: cyanidin 3-glucoside; pelargonidin 3-glucoside; pelargonidin 3-rutinoside | (Cheel et al., 2005; Wang and Lewers, 2007; Simirgiotis et al., 2009; Salvatierra et al., 2010, 2013; Simirgiotis and Schmeda-Hirschmann, 2010; Muñoz et al., 2011; López et al., 2018; Chamorro et al., 2019; Han et al., 2019; Noriega et al., 2021) |
|  | **Apocarotenoids:** 3-oxo-*α*-ionol | (Prat et al., 2014) |
|  | **Flavonoids:** astragalin; benzophenone; catechin; epicatechin; isorhamnetin 3-glucoside; kaempferol; nicotiflorin; procyanidin B; quercetin; quercetin 3-glucoside; quercetin 3-glucuronide | (Cheel et al., 2005; Wang and Lewers, 2007; Simirgiotis et al., 2009; Simirgiotis and Schmeda-Hirschmann, 2010; Muñoz et al., 2011; Salvatierra et al., 2013; Prat et al., 2014; Thomas-Valdés et al., 2019; López et al., 2018; Thomas-Valdés et al., 2018; Han et al., 2019; Noriega et al., 2021) |
|  | **Monoterpenoids:** L-carvomenthone; D-carvone; 1,8-cineole; estragole; D-limonene; linalool; 8-hydroxylinalool; isobornyl acetate; *p*-menthan-3-one; 2,6-dimethyl-7-octene-1,6-diol; *γ*-terpinene; *α*-terpineol | (Prat et al., 2014; Noriega et al., 2021) |
|  | **Phenolic acids:** benzyl benzoate; ellagic acid; pedunculagin | (Cheel et al., 2005; Wang and Lewers, 2007; Simirgiotis et al., 2009; Simirgiotis and Schmeda-Hirschmann, 2010; Prat et al., 2014; López et al., 2018; Thomas-Valdés et al., 2018; Noriega et al., 2021) |
|  | **Phenylethanoids:** 2-phenylethanol | (Prat et al., 2014; Noriega et al., 2021) |
|  | **Phenylpropanoids**: anethole; benzyl acetate; cinnamic acid; 1-*O*-*E*-cinnamoyl-*β*-D-rhamnopyranoside; 1-*O*-*E*-cinnamoyl-R-xylofuranosyl-(1→6)-*β*-D-glucopyranose; 1-*O*-*E*-cinnamoyl-*β*-D-xylopyranoside; cinnamyl alcohol; cinnamyl acetate; hydroxycinnamyl alcohol; hydroxycinnamyl acetate; *p*-coumaric acid 4-glucoside; 3-phenylpropanal; styrene; vanillin | (Cheel et al., 2005; Muñoz et al., 2011; Prat et al., 2014; Noriega et al., 2021) |
|  | **Sesquiterpenoids:** *α*-amorphene; *α*-farnesene; farnesol; *α*-muurolene | (Prat et al., 2014; Noriega et al., 2021) |
|  | **Triterpenoids:** squalene | (Prat et al., 2014) |
| *Gaultheria mucronata* (L.f.) Hook. & Arn. [Ericaceae] | **Anthocyanins:** cyanidin 3-galactoside; cyanidin 3-glucoside; cyanidin 3-lathyroside; delphinidin 3-galactoside | (Ruiz et al., 2013a) |
|  | **Flavonoids:** quercitrin | (Ruiz et al., 2015) |
|  | **Phenylpropanoids:** chlorogenic acid; neochlorogenic acid | (Ruiz et al., 2015) |
| *Gaultheria poeppigii* DC. (syn. *Pernettya myrtilloides* Zucc. ex Steud.) [Ericaceae] | **Anthocyanins**: cyanidin 3-arabinoside; cyanidin 3-galactoside; delphinidin-3-arabinoside; delphinidin 3-galactoside | (Mieres-Castro et al., 2019, 2022; Oyarzún et al., 2020) |
|  | **Flavonoids**: astragalin; hyperoside; quercetin; quercetin-3-O-arabinoside; quercetin-3-O-glucoside; quercetin 3-glucuronide; quercitrin | (Mieres-Castro et al., 2019, 2022; Oyarzún et al., 2020) |
|  | **Monoterpenoids:** 6*α*-hydroxydihydromonotropein-10-*trans*-cinnamate; monotropein-10-*trans*-cinnamate; vaccinoside | (Mieres-Castro et al., 2019, 2022) |
|  | **Phenylpropanoids**: caffeoylglucaric acid; 5-O-caffeoylshikimic acid; chlorogenic acid; neochlorogenic acid; 5-*p*-coumaroylquinic acid | (Mieres-Castro et al., 2019, 2022; Oyarzún et al., 2020) |
| *Geoffroea decorticans* (Gillies ex Hook. & Arn.) Burkat [Fabaceae] | **Flavonoids:** apigenin; apigenin 7-glucoside; baicalin; kaempferol; luteolin; quercetin; quercetin 7-rhamnoside; quercetin 3-glucoside-7-rhamnoside; quercitrin; rutin | (Silva et al., 1999; Costamagna et al., 2016; Jiménez-Aspee et al., 2017) |
|  | **Meroterpenoids:** *α*-tocopherol; *γ*-tocopherol | (Cittadini et al., 2021) |
|  | **Phenolic acids:** gallic acid; protocatechuic acid; quinic acid; syringic acid | (Costamagna et al., 2016; Cittadini et al., 2021) |
|  | **Phenylpropanoids:** 3,5-dicaffeoylquinic acid; 4,5-dicaffeoylquinic acid; *p*-coumaric acid; 5-*p*-coumaroylquinic acid; vanillic acid; vanillin | (Costamagna et al., 2016, 2017; Jiménez-Aspee et al., 2017; Cittadini et al., 2021) |
|  | **Steroids:** Δ^5^-avenasterol*; Δ^7^-avenasterol*; campesterol*; cholesterol*; *β*-sitosterol*; stigmasterol*; Δ^7^-stigmasterol*  *reported only in seeds | (Lamarque et al., 2000; Maestri et al., 2002) |
| *Greigia sphacelata* Ruiz & Pav.) Regel [Bromeliaceae] | **Coumarins:** esculetin-7-O-glucuronide; 7-hydroxycoumarin glucuronide; scopoletin 7-O-glucuronide  **Diterpenoids:** marrubiin  **Flavonoids:** catechin; diffutidin; procyanidin A1; procyanidin B1  **Isoflavonoids:** daidzein-7-O-galactoside; daidzin; genistein-7-O-di-glucoside; genistein-7-O-di-galactoside; genistin; lupinisoflavone A; ononin; tectoridin  **Meroterpenoids:** evodinnol  **Monoterpenoids:** ebuloside; jatamanvaltrate H  **Phenolic acids:** bis(2-hydroxyethyl) phthalate; glucosyringic acid; quinic acid  **Phenylpropanoids:** 2-caffeoylisocitric acid; 1-O-*trans*-*p*-coumaroylglycerol; 1,3-O-di-*trans*-*p*-coumaroylglycerol  **Phloroglucinols:** congestiflorone  **Sesquiterpenoids:** dictamnoside N; euonyminol  **Stilbenoids:** amurensin  **Triterpenoids:** quillaic acid | (Barrientos et al., 2020) |
| *Luma apiculata* (DC.) Burret (syn. *Myrceugenella apiculata* (DC.) Kausel) [Myrtaceae] | **Anthocyanins:** cyanidin 3-glucoside; delphinidin 3-arabinoside; delphinidin 3-galactoside; malvidin 3-arabinoside; malvidin 3-galactoside; malvidin 3-glucoside; malvidin 3-(6΄΄acetyl) galactoside; peonidin 3-arabinoside; peonidin 3-galactoside; peonidin 3-glucoside; petunidin 3-arabinoside; petunidin 3-glucoside | (Simirgiotis et al., 2013; Brito et al., 2014; Ramirez et al., 2015; Fuentes et al., 2016) |
|  | **Flavonoids:** catechin; epicatechin; epigallocatechin gallate; hyperoside; isorhamnetin; isorhamnetin 3-glactoside; myricetin; myricetin 3-galactoside; myricitrin; quercetin; quercetin 3-glucoside; rutin | (Simirgiotis et al., 2013; Brito et al., 2014; Ramirez et al., 2015; Fuentes et al., 2016; Viktorová et al., 2020; Velásquez et al., 2022) |
|  | **Phenolic acids:** gallic acid; syringic acid | (Velásquez et al., 2022) |
|  | **Phenylpropanoids:** caffeic acid; chlorogenic acid; cinnamic acid; ferulic acid; 3-feruloylquinic acid; 4-feruloylquinic acid; neochlorogenic acid | (Brito et al., 2014; Ramirez et al., 2015; Velásquez et al., 2022) |
| *Neltuma alba* (Griseb.) C.E.Hughes & G.P.Lewis (syn. *Prosopis alba* Griseb.) [Fabaceae] | **Flavonoids:** apigenin; isoschaftoside; isovitexin; luteolin; luteolin 7-glucoside; 3-methylquercetin; myricetin 3-glucoside; narcissin; quercetin**; quercetin 3-glucoside; rutin; schaftoside; thermopsoside; vicenin II**; vitexin**  **reported also in fermented beverages after spontaneous fermentation | (Pérez et al., 2014; Cattaneo et al., 2016, 2019; Picariello et al., 2017; Young et al., 2017; Rodriguez et al., 2019; Rodríguez et al., 2020; Correa Uriburu et al., 2022) |
|  | **Monoterpenoids:** linalool oxide A; linalool oxide B | (Takeoka et al., 2008) |
|  | **Nicotinic acid alkaloids:** pyridine | (Takeoka et al., 2008) |
|  | **Phenolic acids:** methyl salicylate | (Takeoka et al., 2008) |
|  | **Phenylpropanoids:** benzylideneacetone; methyl cinnamate; cinnamic acid**; *p*-coumaric acid; ferulic acid; guaiacol; 4-vinyl-2-methoxyphenol  **reported also in fermented beverages after spontaneous fermentation | (Takeoka et al., 2008; Rodriguez et al., 2019; Rodríguez et al., 2020) |
|  | **Proline alkaloids:** 2-acetylpyrrole | (Takeoka et al., 2008) |
|  | **Steroids:** Δ^5^-avenasterol*; Δ^7^-avenasterol*; campesterol*; cholesterol*; *β*-sitosterol*; stigmasterol*; Δ^7^-stigmasterol*  *reported only in seeds | (Lamarque et al., 1994) |
|  | **Tetramate alkaloids:** ethylpyrazine; 2,5-dimethyl-3-ethylpyrazine; methylpyrazine; 2-ethyl-5-methylpyrazine; 2-ethyl-6-methylpyrazine; 2,3-dimethylpyrazine; 2,5-dimethylpyrazine; 2,6-dimethylpyrazine; 2,3,5-trimethylpyrazine; 2-propy1-3,6-dimethylpyrazine; 2-methyl-6-vinylpyrazine | (Takeoka et al., 2008) |
|  | **Tryptophan alkaloids:** tryptamine | (Pérez et al., 2014) |
| *Neltuma chilensis (Molina) C.E.Hughes & G.P.Lewis* (syn. *Prosopis chilensis* (Molina) Stuntz) [Fabaceae] | **Flavonoids:** isovitexin | (Schmeda-Hirschmann et al., 2015) |
|  | **Steroids:** Δ^5^-avenasterol*; Δ^7^-avenasterol*; campesterol*; cholesterol*; *β*-sitosterol*; stigmasterol*; Δ^7^-stigmasterol*  *reported only in seeds | (Lamarque et al., 1994) |
|  | **Tyrosine alkaloids:** L-dopa | (Rajaram and Janardhanan, 1991) |
| *Otholobium glandulosum* (L.) J.W.Grimes (syn. *Psoralea glandulosa* L.) [Fabaceae] | **Meroterpenoids:** bakuchiol*; cyclobakuchiol A; cyclobakuchiol B; 3-hydroxybakuchiol*; 12-hydroxybakuchiol*  **Sesquiterpenoids:** caryophyllene oxide  *chemical superclass not identified by NPClassifier (Kim et al., 2021a) | (Madrid et al., 2013) |
| *Peumus boldus* Molina [Monimiaceae] | **Flavonoids:** catechin; chrysin; epicatechin; quercetin; rutin | (Velásquez et al., 2017; Otero et al., 2022) |
|  | **Phenolic acids**: chlorogenic acid; 3-hydroxybenzoic acid; syringic acid | (Otero et al., 2022) |
|  | **Phenylpropanoids**: caffeic acid; *p*-coumaric acid; sinapic acid | (Velásquez et al., 2017; Otero et al., 2022) |
|  | **Tyrosine alkaloids**: boldine; higenamine; isocorydine; laurolitsine; N-methyllaurotetanine | (Otero et al., 2022) |
| *Prumnopitys andina* (Poepp. ex Endl.) de Laub. (syn. *Podocarpus andinus* Poepp. ex Endl.) [Podocarpaceae] | **Flavonoids:** orientin  **Phenylpropanoids:** chlorogenic acid; 5-*p*-coumaroylquinic acid; 3,5-dicaffeoylquinic acid  **Steroids:** 20-hydroxyecdysone | (Jiménez-Aspee et al., 2019) |
| *Ribes magellanicum* Poir. [Grossulariaceae] | **Anthocyanins:** cyanidin 3-glucoside; cyanidin 3-rutinoside; delphinidin 3-glucoside; delphinidin 3-rutinoside | (Medrano et al., 1985; Ruiz et al., 2013a; Burgos-Edwards et al., 2017, 2018; Theoduloz et al., 2018) |
|  | **Flavonoids:** astragalin; catechin; epicatechin; epigallocatechin; hyperoside; isovitexin; kaempferol; quercetin; quercetin 3-glucoside; quercetin 3-rhamnoside-7-glucoside; rutin | (Ruiz et al., 2015; Jiménez-Aspee et al., 2016b; Burgos-Edwards et al., 2017, 2018) |
|  | **Phenylpropanoids:** chlorogenic acid; *p*-coumaric acid; 3-*p*-coumaroylquinic acid; 4-*p*-coumaroylquinic acid; 5-*p*-coumaroylquinic acid; cryptochlorogenic acid; 3-O-feruloylquinic acid; neochlorogenic acid | (Ruiz et al., 2015; Burgos-Edwards et al., 2017, 2018; Theoduloz et al., 2018) |
| *Rubus geoides* Sm. [Rosaceae] | **Anthocyanins:** cyanidin 3-glucoside; cyanidin 3-sambubioside; cyanidin 3-sophoroside | (Ruiz et al., 2013a) |
|  | **Flavonoids:** catechin; hyperoside; quercetin | (Ruiz et al., 2015; Jiménez-Aspee et al., 2016a) |
|  | **Phenolic acids**: ellagic acid | (Jiménez-Aspee et al., 2016a) |
| *Schinus molle* L. [Anacardiaceae] | **Anthocyanins:** cyanidin 3-galactoside; 7-methylcyanidin 3-galactoside; pelargonidin 3-galactoside; 7-methylpelargonidin 3-galactoside | (Feuereisen et al., 2017) |
|  | **Apocarotenoids:** 4-(2-hydroxy-2,6,6-trimethylcyclohexyl)-3-buten-2-one; 2-hydroxy-2,4,4-trimethyl-3-(3-methylbuta-1,3-dienyl)cyclohexanone | (Al-Andal et al., 2019) |
|  | **Carotenoids:** *β*-carotene; *β*-cryptoxanthin; lutein; lycopene; phytoene; phytofluene | (Giuffrida et al., 2020) |
|  | **Diterpenoids:** cembrene; 4,8,13-duvatriene-1,3-diol; kaur-15-en-19-ol acetate; kaur-16-ene; ent-16-kauren-19-ol acetate; neocembrene; thunbergol | (Bendaoud et al., 2010; Al-Andal et al., 2019) |
|  | **Flavonoids:** agathisflavone; amentoflavone; 2'',3''-dihydroamentoflavone; tetrahydroamentoflavone; 7-O-methylapigenin; catechin; catechin 3-gallate; chamaejasmin; engeletin; epicatechin; hinokiflavone; kaempferol; luteolin; masazinoflavanone; neochamaejasmin B; quercetin; quercetin 3-glucoside; quercetin 3-O-glucuronide; quercetin 3-lathyroside; quercetin 3-(2-galloylglucoside); quercitrin; robustaflavone; rutin | (Yueqin et al., 2003; Ono et al., 2008; Feuereisen et al., 2017; Tlili et al., 2018; Volpini-Klein et al., 2020; Feriani et al., 2021, 2022; Kim et al., 2021b; Osman et al., 2021) |
|  | **Lysine alkaloids:** piperine | (Kim et al., 2021b) |
|  | **Monoterpenoids:** borneol; bornyl acetate; camphene; *α*-campholenal; camphor; 2-carene; 3-carene; *trans*-3-caren-2-ol; carvacrol; carveol; carvotanacetone; 1,8-cineole; 2-acetoxy-1,8-cineole; citronellol; methyl citronellate; citronellyl acetate; cryptone; 3,5-dimethylcyclohexanol; *o*-cymene; *p*-cymene; *p*-cymen-8-ol; dihydrocarveol; (*E*)-2,3-epoxycarane; *α*-fenchene; fenchyl acetate; geraniol; geranyl acetate; geranyl butyrate; geranyl propionate; (*E*)-methyl geranate; grandisol; ipsdienone; isoborneol; isomenthone; isomyrcenol; isopinocamphone; isopiperitenol; 4-isopropylbenzaldehyde; limonene; limonene-1,2-epoxide; linalool; linalool oxide A; linalyl acetate; linalyl butyrate; lineatin; *cis*-*p*-menth-2-en-1-ol; *trans*-*p*-menth-2-en-1-ol; *cis*-*p*-mentha-2,8-dien-1-ol; *trans*-*p*-mentha-2,8-dien-1-ol; *cis*-1(7),8- *p*-menthadien-2-ol; *trans*-1(7),8- *p*-menthadien-2-ol; *p*-mentha-1,8-dien-4-ol; *p*-menth-3-en-2-one; *β*-myrcene; myrtenal; myrtenol; 6-isopropenyl-4,8a-dimethyl-1,2,3,5,6,7,8,8a-octahydro-naphthalen-2-ol; neral; neryl butyrate; (3*E*)-2,6-dimethylocta-3,7-diene-2,6-diol; *cis*-*β*-ocimene; *trans*-*β*-ocimene; *allo*-ocimene; *neo*-*allo*-ocimene; 2,6-dimethylocta-1,5,7-trien-3-ol; *α*-phellandrene; *α*-phellandrene epoxide; *β*-phellandrene; phellandral; pinanediol; *α*-pinene; *β*-pinene; *β*-pinene oxide; pinocamphone; *trans*-pinocarveol; *cis*-piperitol; *trans*-piperitol; piperitone; sabinene; sabinol; sabinyl acetate; dehydrosabinaketone; sylvestrene; *α*-terpinene; *γ*-terpinene; terpinen-4-ol; *α*-terpineol; *α*-terpinolene; *α*-terpinyl acetate; *α*-thujene; *α*-thujenal; *α*-thujone; *β*-thujone; thymol; tricyclene; *trans*-verbenyl acetate | (Bernhard et al., 1983; Maffei and Chialva, 1990; Baser et al., 1997; Huaman et al., 2004; Hayouni et al., 2008; Atti dos Santos Santos et al., 2009; Abdel-Sattar et al., 2010; Zahed et al., 2010, 2011; Bendaoud et al., 2010; Hosni et al., 2011; Pérez-López et al., 2011; Rocha et al., 2012; Guerra-Boone et al., 2013; Martins et al., 2014; dos Santos Cavalcanti et al., 2015; Hamdan et al., 2016; Eryigit et al., 2017; Kasmi et al., 2017; Rey-Valeirón et al., 2017; Aboalhaija et al., 2019b, 2019a; Giuffrida et al., 2020; Osman et al., 2021; Volpini-Klein et al., 2021; Belhoussaine et al., 2022; Chaaban et al., 2022) |
|  | **Phenolic acids:** *p*-anisaldehyde; benzoic acid; *p*-aminobenzoic acid; 4-hydroxybenzoic acid; ellagic acid; eucaglobulin; gallic acid; pentagalloylglucose; *β*-glucogallin; protocatechuic acid; quinic acid; methyl salicylate; salicylic acid; shikimic acid; syringaldehyde; syringic acid | (Bendaoud et al., 2010; Galvez Ranilla et al., 2010; Feuereisen et al., 2017; Tlili et al., 2018; Feriani et al., 2021, 2022; Kim et al., 2021b; Osman et al., 2021) |
|  | **Phenylpropanoids:** anethole; chlorogenic acid; methyl *trans*-cinnamate; *p*-coumaric acid; ferulic acid; vanillic acid | (Galvez Ranilla et al., 2010; Eryigit et al., 2017; Tlili et al., 2018; Volpini-Klein et al., 2020; Kim et al., 2021b) |
|  | **Sesquiterpenoids:** *α*-acorenol; 4-acoren-3-one; agarospirol; amorpha-4,9-dien-2-ol; *α*-amorphene; aromadendrene; *allo*-aromadendrene; *cis*-*α*-bergamotene; *trans*-*α*-bergamotene; bicyclogermacrene; *epi*-bicyclosesquiphellandrene; *β*-bisabolene; cis-(Z)-*α*-bisabolene epoxide; *α*-bisabolenol; *β*-bisabolol; 1-endo-bourbonanol; *β*-bourbonene; bulnesol; cadina-1,4-diene; *α*-cadinene; *γ*-cadinene; *δ*-cadinene; *α*-cadinol; *epi*-*α*-cadinol; *α*-cadinol methyl ether; *δ*-cadinol; *τ*-cadinol; *α*-calacorene; *β*-calacorene; *cis*-calamenene; *trans*-calamenene; carotol; *cis*-*β*-caryophyllene; *trans*-*β*-caryophyllene; (*E*)-*epi*-*β*-caryophyllene; caryophyllene acetate; caryophyllene oxide; 4,8-epoxy- *β*-caryophyllene; cedrelanol; *α*-cedrene; chrysothol; copaborneol; *α-*copaene; *β*-copaene; *β*-copaen-4- *α*-ol; *δ*-copaene; *α*-cubebene; *β*-cubebene; cubebol; 4-*epi*-cubebol; cubenol; 1,10-di-epicubenol; 1-*epi*-cubenol; *γ*-curcumene; *trans*-dauc-8-en-4- *β*-ol; 9,10-dimethyltricyclo[4.2.1.1(2,5)]decane-9,10-diol; *β*-elemene; *γ*-elemene; elemol; 10-*epi*-elemol; 7*α*H-eudesma-3,5-diene; *β*-eudesmene; 1*β*,6*α*-dihydroxy-7-*epi*-eudesm-3-ene; 1*β*,6*β*-dihydroxy-7-*epi*-eudesm-3-ene; *α*-eudesmol; *β*-eudesmol; *γ*-eudesmol; 8-*epi*-*γ*-eudesmol; *α*-farnesene; *β*-farnesene; farnesol; germacrene A; germacrene B; germacrene D; germacrene D-4-ol; 1,6-germacradien-5-ol; gleenol; globulol; epiglobulol; 4*β*,6*β*-dihydroxy-10*α*-methoxy-1*α*,5*β*,7*α*(*H*)-guaiane; *β*-guaiene; 4*β*,6*β*-dihydroxy-1*α*,5*β*(*H*)-guai-9-ene; guaia-3,7-diene; guaiol; guaiol acetate; *a*-gurjunene; *β*-gurjunene; *γ*-gurjunene; *τ*-gurjunene; hedycaryol; 5-methyl-1-phenylbicyclo[3.2.0]heptane; *α*-himachalene; *α*-humulene; humulene-6,7-epoxide; isoaromadendrene epoxide; isocalamendiol; dehydroxyisocalamendiol; isoledene; juniper camphor; ledene; ledol; longifolenaldehyde; longifolene; *α*-muurolene; *γ*-muurolene; *α*-muurol; *epi*-*α*-muurol; *τ*-muurolol; *cis*-nerolidol; *trans*-nerolidol; nootkatol; oplodiol; oplopanone; *β*-oplopenone; palustrol; platambin; 2-(4-ethenyl-4-methyl-3-prop-1-en-2-ylcyclohexyl)propan-2-ol; 2-[(2S,4*α*S)-4*α*-methyl-8-methylidene-1,2,3,4,5,6,7,8*α*-octahydronaphthalen-2-yl]propan-2-ol; 1-methyl-3-(2,2,6-trimethyl-bicyclo[4.1.0]hept-1-yl)-propenyl acetate; *α* -selinene; *β*-selinene; *δ*-selinene; shyobunol; *β*-spathulene; spathulenol; teucladiol; teuclatriol; 10-*epi*-teuclatriol; valencene; valerianol; viridiflorol; *α*-ylangene | (Terhune et al., 1974; Bernhard et al., 1983; Maffei and Chialva, 1990; Baser et al., 1997; Huaman et al., 2004; Hayouni et al., 2008; Atti dos Santos Santos et al., 2009; Abdel-Sattar et al., 2010; Zahed et al., 2010, 2011; Bendaoud et al., 2010; Hosni et al., 2011; Pérez-López et al., 2011; Rocha et al., 2012; Guerra-Boone et al., 2013; Martins et al., 2014; dos Santos Cavalcanti et al., 2015; Hamdan et al., 2016; Eryigit et al., 2017; Kasmi et al., 2017; Rey-Valeirón et al., 2017; Aboalhaija et al., 2019b, 2019a; Al-Andal et al., 2019; Giuffrida et al., 2020; Osman et al., 2021; Volpini-Klein et al., 2021; Belhoussaine et al., 2022; Chaaban et al., 2022) |
|  | **Steroids:** *β*-sitosterol | (Al-Andal et al., 2019) |
|  | **Triterpenoids:** elemonic acid; isomasticadienolic acid; 3- *epi*-isomasticadienolic acid; isomasticadienonalic acid; isomasticadienonic acid; 3- *epi*-isomasticadienolalic acid; masticadienonic acid; (24*Z*)-3*α*-hydroxy-7-oxo-8,24-tirucalladiene-26-oic acid | (Pozzo-Balbi et al., 1978; Yueqin et al., 2003; Ono et al., 2008) |
| *Schinus polygama* (Cav.) Cabrera (syn. *Duvaua dependens* DC.) [ Anacardiaceae] | **Monoterpenoids:** borneol; bornyl acetate; camphene; camphor; *p*-cymene; *α*-fenchol; limonene; linalool; myrtenol; *α*-pinene; *β*-pinene; *trans*-pinocarveol; terpinene-4-ol; *α*-terpineol; *α*-thujene  **Sesquiterpenoids:** *epi*-bicyclosesquiphellandrene; *γ*-cadinene; *δ*-cadinene; *δ*-cadinol; *β*-caryophyllene; *α*-copaene; *α*-cubebene; elemol; *α*-humulene; isolongifolol; *α*-muurolene; selina-3,11-diene-6-*α*-ol | (Erazo et al., 2006) |
| *Ugni molinae* Turcz. (syn. *Ugni philippii* O.Berg, *Ugni poeppigii* O.Berg) [Myrtaceae] | **Anthocyanins:** cyanidin 3-arabinoside; cyanidin 3-galactoside; cyanidin 3-glucoside; cyanidin 3-rutinoside; cyanidin 3-(6΄΄-succinoyl) glucoside; delphinidin 3-arabinoside; delphinidin 3-glucoside; malvidin 3-glucoside; pelargonidin 3-arabinoside; peonidin 3-arabinoside; peonidin 3-glucoside; petunidin 3-galactoside; petunidin 3-glucoside; petunidin 3-rutinoside | (Ruiz et al., 2010; Brito et al., 2014; Junqueira-Gonçalves et al., 2015; Ramirez et al., 2015; López et al., 2019) |
|  | **Carotenoids:** *β*-carotene | (López et al., 2017b) |
|  | **Flavonoids:** astragalin; catechin; epicatechin; hyperoside; isorhamnetin; kaempferol; luteolin; myricetin; quercetin; quercetin 3-glucoside; quercitrin; rutin | (Brito et al., 2014; Junqueira-Gonçalves et al., 2015; Ramirez et al., 2015; Jofré et al., 2016, 2019; López de Dicastillo et al., 2017; López et al., 2017b, 2019; Pérez-Arancibia et al., 2021; Gómez-Pérez et al., 2022) |
|  | **Meroterpenoids:** *α*-tocopherol; *β*-tocopherol; *γ*-tocopherol; *δ*-tocopherol | (López et al., 2017a) |
|  | **Monoterpenoids:** 1,8-cineole; limonene; *α*-pinene | (Scheuermann et al., 2008) |
|  | **Phenolic acids:** ellagic acid; gallic acid; 4-hydroxybenzoic acid; protocatechuic acid; syringic acid | (Junqueira-Gonçalves et al., 2015; Jofré et al., 2016, 2019; López de Dicastillo et al., 2017; López et al., 2017b, 2019; Pérez-Arancibia et al., 2021; Gómez-Pérez et al., 2022) |
|  | **Phenylethanoids:** hydroxytyrosol; tyrosol | (López et al., 2019) |
|  | **Phenylpropanoids:** caffeic acid 3-glucoside; chlorogenic acid; *trans*-cinnamic acid; *p*-coumaric acid; ferulic acid; 3-feruloylquinic acid; 4-feruloylquinic acid; neochlorogenic acid | (Brito et al., 2014; Junqueira-Gonçalves et al., 2015; Ramirez et al., 2015; López et al., 2017b, 2019; Gómez-Pérez et al., 2022) |
|  | **Stilbenoids:** *trans*-resveratrol | (Salazar et al., 2017) |

**References**

Abdel-Sattar, E., Zaitoun, A. A., Farag, M. A., Gayed, S. H. El, and Harraz, F. M. H. (2010). Chemical composition, insecticidal and insect repellent activity of Schinus molle L. leaf and fruit essential oils against Trogoderma granarium and Tribolium castaneum. *Nat. Prod. Res.* 24, 226–235. doi: 10.1080/14786410802346223

Aboalhaija, N. H., Amro, R., Abaza, I. F., Khalil, Al-Aboudi, A., Abu-Zarga, M., et al. (2019a). Schinus molle L. Collected from Jordan and Turkey: Essential Oil Composition and Anticholinesterase Activity. *J. Essent. Oil Bear. Plants* 22, 704–716. doi: 10.1080/0972060X.2019.1639552

Aboalhaija, N. H., Awwad, O., Khalil, E., Abbassi, R., Abaza, I. F., and Afifi, F. U. (2019b). Chemodiversity and Antiproliferative Activity of the Essential Oil of Schinus molle Growing in Jordan. *Chem. Biodivers.* 16, e1900388. doi: https://doi.org/10.1002/cbdv.201900388

Agulló, V., González-Trujano, M. E., Hernandez-Leon, A., Estrada-Camarena, E., Pellicer, F., and García-Viguera, C. (2021a). Antinociceptive effects of maqui-berry (Aristotelia chilensis (Mol.) Stuntz). *Int. J. Food Sci. Nutr.* 72, 947–955. doi: 10.1080/09637486.2021.1895727

Agulló, V., González-Trujano, M. E., Hernandez-Leon, A., Estrada-Camarena, E., Pellicer, F., and García-Viguera, C. (2021b). Synergistic Interaction in the Analgesic-Like Effects of Maqui Berry and Citrus Is Antagonized by Sweeteners. *Nutrients* 13. doi: 10.3390/nu13072466

Al-Andal, A., Moustafa, M., and Alrumman, S. (2019). Variations in Chemicals and Antimicrobial Properties of Schinus molle Fruits Grown in Abha Area, Saudi Arabia. *Arab. J. Sci. Eng.* 44, 87–101. doi: 10.1007/s13369-018-3660-x

Arena, M. E., Postemsky, P. D., and Curvetto, N. R. (2017). Changes in the phenolic compounds and antioxidant capacity of Berberis microphylla G. Forst. berries in relation to light intensity and fertilization. *Sci. Hortic. (Amsterdam).* 218, 63–71. doi: https://doi.org/10.1016/j.scienta.2017.02.004

Atti dos Santos Santos, A. C., Rossato, M., Agostini, F., Atti Serafini, L., dos Santos, P. L., Molon, R., et al. (2009). Chemical composition of the essential oils from leaves and fruits of Schinus molle L. and Schinus terebinthifolius Raddi from Southern Brazil. *J. Essent. Oil Bear. Plants* 12, 16–25.

Barrientos, R. E., Ahmed, S., Cortés, C., Fernández-Galleguillos, C., Romero-Parra, J., Simirgiotis, M. J., et al. (2020). Chemical Fingerprinting and Biological Evaluation of the Endemic Chilean Fruit Greigia sphacelata (Ruiz and Pav.) Regel (Bromeliaceae) by UHPLC-PDA-Orbitrap-Mass Spectrometry. *Molecules* 25. doi: 10.3390/molecules25163750

Baser, K. H. C., Kürkçüoglu, M., Demirçakmak, B., Uülker, N., and Beis, S. H. (1997). Composition of the Essential Oil of Schinus molle L. Grown in Turkey. *J. Essent. Oil Res.* 9, 693–696. doi: 10.1080/10412905.1997.9700813

Bastías-Montes, J. M., Vidal-San-Martín, C., Tamarit-Pino, Y., Muñoz-Fariña, O., García-Figueroa, O., Quevedo-León, R., et al. (2022). Cryoconcentration by Centrifugation&ndash;Filtration: A Simultaneous, Efficient and Innovative Method to Increase Thermosensitive Bioactive Compounds of Aqueous Maqui (Aristotelia chilensis (Mol.) Stuntz) Extract. *Processes* 10. doi: 10.3390/pr10010025

Bastías-Montes, J. M., Vidal-San Martín, C., Muñoz-Fariña, O., Petzold-Maldonado, G., Quevedo-León, R., Wang, H., et al. (2019). Cryoconcentration procedure for aqueous extracts of maqui fruits prepared by centrifugation and filtration from fruits harvested in different years from the same localities. *J. Berry Res.* 9, 377–394. doi: 10.3233/JBR-180368

Belhoussaine, O., El Kourchi, C., Harhar, H., Bouyahya, A., El Yadini, A., Fozia, F., et al. (2022). Chemical Composition, Antioxidant, Insecticidal Activity, and Comparative Analysis of Essential Oils of Leaves and Fruits of *Schinus molle* and *Schinus terebinthifolius*. *Evidence-Based Complement. Altern. Med.* 2022, 4288890. doi: 10.1155/2022/4288890

Bendaoud, H., Romdhane, M., Souchard, J. P., Cazaux, S., and Bouajila, J. (2010). Chemical Composition and Anticancer and Antioxidant Activities of Schinus Molle L. and Schinus Terebinthifolius Raddi Berries Essential Oils. *J. Food Sci.* 75, C466–C472. doi: https://doi.org/10.1111/j.1750-3841.2010.01711.x

Bernhard, R. A., Shibamoto, T., Yamaguchi, K., and White, E. (1983). The volatile constituents of Schinus molle L. *J. Agric. Food Chem.* 31, 463–466. doi: 10.1021/jf00116a075

Boeri, P., Piñuel, L., Dalzotto, D., Monasterio, R., Fontana, A., Sharry, S., et al. (2020). Argentine Patagonia barberry chemical composition and evaluation of its antioxidant capacity. *J. Food Biochem.* 44, e13254. doi: https://doi.org/10.1111/jfbc.13254

Brauch, J. E., Buchweitz, M., Schweiggert, R. M., and Carle, R. (2016). Detailed analyses of fresh and dried maqui (Aristotelia chilensis (Mol.) Stuntz) berries and juice. *Food Chem.* 190, 308–316. doi: https://doi.org/10.1016/j.foodchem.2015.05.097

Brauch, J. E., Reuter, L., Conrad, J., Vogel, H., Schweiggert, R. M., and Carle, R. (2017). Characterization of anthocyanins in novel Chilean maqui berry clones by HPLC–DAD–ESI/MSn and NMR-spectroscopy. *J. Food Compos. Anal.* 58, 16–22. doi: https://doi.org/10.1016/j.jfca.2017.01.003

Brito, A., Areche, C., Sepúlveda, B., Kennelly, E. J., and Simirgiotis, M. J. (2014). Anthocyanin Characterization, Total Phenolic Quantification and Antioxidant Features of Some Chilean Edible Berry Extracts. *Molecules* 19, 10936–10955. doi: 10.3390/molecules190810936

Burgos-Edwards, A., Jiménez-Aspee, F., Theoduloz, C., and Schmeda-Hirschmann, G. (2018). Colonic fermentation of polyphenols from Chilean currants (Ribes spp.) and its effect on antioxidant capacity and metabolic syndrome-associated enzymes. *Food Chem.* 258, 144–155. doi: https://doi.org/10.1016/j.foodchem.2018.03.053

Burgos-Edwards, A., Jiménez-Aspee, F., Thomas-Valdés, S., Schmeda-Hirschmann, G., and Theoduloz, C. (2017). Qualitative and quantitative changes in polyphenol composition and bioactivity of Ribes magellanicum and R. punctatum after in vitro gastrointestinal digestion. *Food Chem.* 237, 1073–1082. doi: https://doi.org/10.1016/j.foodchem.2017.06.060

Bustamante, L., Pastene, E., Duran-Sandoval, D., Vergara, C., Von Baer, D., and Mardones, C. (2018). Pharmacokinetics of low molecular weight phenolic compounds in gerbil plasma after the consumption of calafate berry (Berberis microphylla) extract. *Food Chem.* 268, 347–354. doi: https://doi.org/10.1016/j.foodchem.2018.06.048

Calfío, C., and Huidobro-Toro, J. P. (2019). Potent Vasodilator and Cellular Antioxidant Activity of Endemic Patagonian Calafate Berries (Berberis microphylla) with Nutraceutical Potential. *Molecules* 24. doi: 10.3390/molecules24152700

Cattaneo, F., Costamagna, M. S., Zampini, I. C., Sayago, J., Alberto, M. R., Chamorro, V., et al. (2016). Flour from Prosopis alba cotyledons: A natural source of nutrient and bioactive phytochemicals. *Food Chem.* 208, 89–96. doi: https://doi.org/10.1016/j.foodchem.2016.03.115

Cattaneo, F., Roco, J., Alarcón, G., Isla, M. I., and Jeréz, S. (2019). <em>Prosopis alba</em> seed flour improves vascular function in a rabbit model of high fat diet-induced metabolic syndrome. *Heliyon* 5. doi: 10.1016/j.heliyon.2019.e01967

Céspedes, C. L., Alarcon, J., Avila, J. G., and Nieto, A. (2010a). Anti-inflammatory Activity of Aristotelia chilensis Mol. (Stuntz) (Elaeocarpaceae). *Boletín Latinoam. y del Caribe Plantas Med. y Aromáticas* 9, 127–135.

Céspedes, C. L., Alarcon, J., Valdez-Morales, M., and Paredes-López, O. (2009). Antioxidant Activity of an Unusual 3-Hydroxyindole Derivative Isolated from Fruits of Aristotelia chilensis (Molina) Stuntz. 64, 759–762. doi: doi:10.1515/znc-2009-9-1024

Céspedes, C. L., Pavon, N., Dominguez, M., Alarcon, J., Balbontin, C., Kubo, I., et al. (2017). The chilean superfruit black-berry Aristotelia chilensis (Elaeocarpaceae), Maqui as mediator in inflammation-associated disorders. *Food Chem. Toxicol.* 108, 438–450. doi: https://doi.org/10.1016/j.fct.2016.12.036

Céspedes, C. L., Valdez-Morales, M., Avila, J. G., El-Hafidi, M., Alarcón, J., and Paredes-López, O. (2010b). Phytochemical profile and the antioxidant activity of Chilean wild black-berry fruits, Aristotelia chilensis (Mol) Stuntz (Elaeocarpaceae). *Food Chem.* 119, 886–895. doi: https://doi.org/10.1016/j.foodchem.2009.07.045

Chaaban, S. Ben, Haouel-Hamdi, S., Bachrouch, O., Mahjoubi, K., and Mediouni Ben Jemâa, J. (2022). Fumigant toxicity of four essential oils against the carob moth Ectomyelois ceratoniae Zeller and the Mediterranean flour moth Ephestia kuehniella. *Int. J. Environ. Health Res.*, 1–13. doi: 10.1080/09603123.2022.2152431

Chamorro, M. F., Reiner, G., Theoduloz, C., Ladio, A., Schmeda-Hirschmann, G., Gómez-Alonso, S., et al. (2019). Polyphenol Composition and (Bio)Activity of Berberis Species and Wild Strawberry from the Argentinean Patagonia. *Molecules* 24. doi: 10.3390/molecules24183331

Cheel, J., Theoduloz, C., Rodríguez, J., Saud, G., Caligari, P. D. S., and Schmeda-Hirschmann, G. (2005). E-Cinnamic Acid Derivatives and Phenolics from Chilean Strawberry Fruits, Fragaria chiloensis ssp. chiloensis. *J. Agric. Food Chem.* 53, 8512–8518. doi: 10.1021/jf051294g

Chen, L., Zhou, G., Meng, X.-S., Fu, H.-Y., Mo, Q.-G., and Wang, Y.-W. (2020). Photoprotection of maqui berry against ultraviolet B-induced photodamage in vitro and in vivo. *Food Funct.* 11, 2749–2762. doi: 10.1039/C9FO01902B

Cittadini, M. C., García-Estévez, I., Escribano-Bailón, M. T., Bodoira, R. M., Barrionuevo, D., and Maestri, D. (2021). Nutritional and nutraceutical compounds of fruits from native trees (Ziziphus mistol and Geoffroea decorticans) of the dry chaco forest. *J. Food Compos. Anal.* 97, 103775. doi: https://doi.org/10.1016/j.jfca.2020.103775

Concha-Meyer, A. A., Sepúlveda, G., Pérez-Díaz, R., and Torres, C. A. (2021). Effect of preservation processing on quality attributes and phenolic profile of maqui (Aristotelia chilensis mol. Stuntz) fruit. *LWT* 149, 111920. doi: https://doi.org/10.1016/j.lwt.2021.111920

Coronado-Olano, J., Repo-Carrasco-Valencia, R., Reategui, O., Toscano, E., Valdez, E., Zimic, M., et al. (2021). Inhibitory activity against α-amylase and α-glucosidase by phenolic compounds of quinoa (Chenopodium quinoa Willd.) and cañihua (Chenopodium pallidicaule Aellen) from the Andean region of Peru. *Pharmacogn. J.* 13.

Correa Uriburu, F. M., Cattaneo, F., Maldonado, L. M., Zampini, I. C., Alberto, M. R., and Isla, M. I. (2022). Prosopis alba Seed as a Functional Food Waste for Food Formulation Enrichment. *Foods* 11. doi: 10.3390/foods11182857

Costamagna, M. S., Gómez-Mascaraque, L. G., Zampini, I. C., Alberto, M. R., Pérez, J., López-Rubio, A., et al. (2017). Microencapsulated chañar phenolics: A potential ingredient for functional foods development. *J. Funct. Foods* 37, 523–530. doi: https://doi.org/10.1016/j.jff.2017.08.018

Costamagna, M. S., Zampini, I. C., Alberto, M. R., Cuello, S., Torres, S., Pérez, J., et al. (2016). Polyphenols rich fraction from Geoffroea decorticans fruits flour affects key enzymes involved in metabolic syndrome, oxidative stress and inflammatory process. *Food Chem.* 190, 392–402. doi: https://doi.org/10.1016/j.foodchem.2015.05.068

del Campo, A., Salamanca, C., Fajardo, A., Díaz-Castro, F., Bustos, C., Calfío, C., et al. (2021). Anthocyanins from Aristotelia chilensis Prevent Olanzapine-Induced Hepatic-Lipid Accumulation but Not Insulin Resistance in Skeletal Muscle Cells. *Molecules* 26. doi: 10.3390/molecules26206149

Di Lorenzo, A., Sobolev, A. P., Nabavi, S. F., Sureda, A., Moghaddam, A. H., Khanjani, S., et al. (2019). Antidepressive effects of a chemically characterized maqui berry extract (Aristotelia chilensis (Molina) Stuntz) in a mouse model of post-stroke depression. *Food Chem. Toxicol.* 129, 434–443. doi: https://doi.org/10.1016/j.fct.2019.04.023

Diaz, L. S., Rosende, C. G., and Antunez, M. I. (1984). Spectrophotometric identification of anthocyanin pigments from “maqui” fruits (Aristotelia chilensis, Mol, Stuntz). *Rev. Agroquim. y Tecnol. Aliment.* 24, 538–550.

dos Santos Cavalcanti, A., de Souza Alves, M., da Silva, L. C. P., dos Santos Patrocínio, D., Sanches, M. N., Chaves, D. S. de A., et al. (2015). Volatiles composition and extraction kinetics from Schinus terebinthifolius and Schinus molle leaves and fruit. *Rev. Bras. Farmacogn.* 25, 356–362. doi: https://doi.org/10.1016/j.bjp.2015.07.003

Erazo, S., Delporte, C., Negrete, R., García, R., Zaldívar, M., Iturra, G., et al. (2006). Constituents and biological activities of Schinus polygamus. *J. Ethnopharmacol.* 107, 395–400. doi: https://doi.org/10.1016/j.jep.2006.03.028

Eryigit, T., Yildirim, B., Ekici, K., and Çirka, M. (2017). Chemical Composition, Antimicrobial and Antioxidant Properties of Schinus molle L. Essential Oil from Turkey. *J. Essent. Oil Bear. Plants* 20, 570–577. doi: 10.1080/0972060X.2017.1304286

Escribano-Bailón, M. T., Alcalde-Eon, C., Muñoz, O., Rivas-Gonzalo, J. C., and Santos-Buelga, C. (2006). Anthocyanins in Berries of Maqui [Aristotelia chilensis (Mol.) Stuntz]. *Phytochem. Anal.* 17, 8–14.

Feriani, A., Tir, M., Aldahmash, W., Mnafgui, K., Hichem, A., Gómez-Caravaca, A. M., et al. (2022). In vivo evaluation and molecular docking studies of Schinus molle L. fruit extract protective effect against isoproterenol-induced infarction in rats. *Environ. Sci. Pollut. Res.* 29, 80910–80925. doi: 10.1007/s11356-022-21422-4

Feriani, A., Tir, M., Mufti, A., Caravaca, A. M. G., Contreras, M. del M., Taamalli, A., et al. (2021). HPLC–ESI–QTOF–MS/MS profiling and therapeutic effects of Schinus terebinthifolius and Schinus molle fruits: investigation of their antioxidant, antidiabetic, anti-inflammatory and antinociceptive properties. *Inflammopharmacology* 29, 467–481. doi: 10.1007/s10787-021-00791-1

Feuereisen, M. M., Zimmermann, B. F., Schulze-Kaysers, N., and Schieber, A. (2017). Differentiation of Brazilian Peppertree (Schinus terebinthifolius Raddi) and Peruvian Peppertree (Schinus molle L.) Fruits by UHPLC–UV–MS Analysis of Their Anthocyanin and Biflavonoid Profiles. *J. Agric. Food Chem.* 65, 5330–5338. doi: 10.1021/acs.jafc.7b00480

Fredes, C., Becerra, C., Parada, J., and Robert, P. (2018a). The Microencapsulation of Maqui (Aristotelia chilensis (Mol.) Stuntz) Juice by Spray-Drying and Freeze-Drying Produces Powders with Similar Anthocyanin Stability and Bioaccessibility. *Molecules* 23. doi: 10.3390/molecules23051227

Fredes, C., Osorio, M. J., Parada, J., and Robert, P. (2018b). Stability and bioaccessibility of anthocyanins from maqui (Aristotelia chilensis [Mol.] Stuntz) juice microparticles. *LWT* 91, 549–556. doi: https://doi.org/10.1016/j.lwt.2018.01.090

Fredes, C., Yousef, G. G., Robert, P., Grace, M. H., Lila, M. A., Gómez, M., et al. (2014). Anthocyanin profiling of wild maqui berries (Aristotelia chilensis [Mol.] Stuntz) from different geographical regions in Chile. *J. Sci. Food Agric.* 94, 2639–2648. doi: https://doi.org/10.1002/jsfa.6602

Fuentes, L., Valdenegro, M., Gómez, M.-G., Ayala-Raso, A., Quiroga, E., Martínez, J.-P., et al. (2016). Characterization of fruit development and potential health benefits of arrayan (Luma apiculata), a native berry of South America. *Food Chem.* 196, 1239–1247. doi: https://doi.org/10.1016/j.foodchem.2015.10.003

Gallia, M. C., Bachmeier, E., Ferrari, A., Queralt, I., Mazzeo, M. A., and Bongiovanni, G. A. (2020). Pehuén (Araucaria araucana) seed residues are a valuable source of natural antioxidants with nutraceutical, chemoprotective and metal corrosion-inhibiting properties. *Bioorg. Chem.* 104, 104175. doi: https://doi.org/10.1016/j.bioorg.2020.104175

Galvez Ranilla, L., Kwon, Y.-I., Apostolidis, E., and Shetty, K. (2010). Phenolic compounds, antioxidant activity and in vitro inhibitory potential against key enzymes relevant for hyperglycemia and hypertension of commonly used medicinal plants, herbs and spices in Latin America. *Bioresour. Technol.* 101, 4676–4689. doi: https://doi.org/10.1016/j.biortech.2010.01.093

Genskowsky, E., Puente, L. A., Pérez-Álvarez, J. A., Fernández-López, J., Muñoz, L. A., and Viuda-Martos, M. (2016). Determination of polyphenolic profile, antioxidant activity and antibacterial properties of maqui [Aristotelia chilensis (Molina) Stuntz] a Chilean blackberry. *J. Sci. Food Agric.* 96, 4235–4242. doi: https://doi.org/10.1002/jsfa.7628

Gironés-Vilaplana, A., Baenas, N., Villaño, D., Speisky, H., García-Viguera, C., and Moreno, D. A. (2014). Evaluation of Latin-American fruits rich in phytochemicals with biological effects. *J. Funct. Foods* 7, 599–608. doi: https://doi.org/10.1016/j.jff.2013.12.025

Gironés-Vilaplana, A., Mena, P., García-Viguera, C., and Moreno, D. A. (2012a). A novel beverage rich in antioxidant phenolics: Maqui berry (Aristotelia chilensis) and lemon juice. *LWT* 47, 279–286. doi: https://doi.org/10.1016/j.lwt.2012.01.020

Gironés-Vilaplana, A., Valentão, P., Moreno, D. A., Ferreres, F., Garcı́a-Viguera, C., and Andrade, P. B. (2012b). New Beverages of Lemon Juice Enriched with the Exotic Berries Maqui, Açaı́, and Blackthorn: Bioactive Components and in Vitro Biological Properties. *J. Agric. Food Chem.* 60, 6571–6580. doi: 10.1021/jf300873k

Giuffrida, D., Martínez, N., Arrieta-Garay, Y., Fariña, L., Boido, E., and Dellacassa, E. (2020). Valorisation of Schinus molle fruit as a source of volatile compounds in foods as flavours and fragrances. *Food Res. Int.* 133, 109103. doi: https://doi.org/10.1016/j.foodres.2020.109103

Gómez-Pérez, L. S., Moraga, N., Ah-Hen, K. S., Rodríguez, A., and Vega-Gálvez, A. (2022). Dietary fibre in processed murta (Ugni molinae Turcz) berries: bioactive components and antioxidant capacity. *J. Food Sci. Technol.* 59, 3093–3101. doi: 10.1007/s13197-022-05416-1

Guerra-Boone, L., Álvarez-Román, R., Salazar-Aranda, R., Torres-Cirio, A., Rivas-Galindo, V. M., de Torres, N. W., et al. (2013). Chemical compositions and antimicrobial and antioxidant activities of the essential oils from Magnolia grandiflora, Chrysactinia mexicana, and Schinus molle found in Northeast Mexico. *Nat. Prod. Commun.* 8, 1934578X1300800133. doi: 10.1177/1934578X1300800133

Hamdan, D. I., Al-Gendy, A. A., and El-Shazly, A. M. (2016). Chemical composition and cytotoxic activity of the essential oils of Schinus molle growing in Egypt. *J. Pharm. Sci. Res.* 8, 779.

Han, Y., Song, M., Gu, M., Ren, D., Zhu, X., Cao, X., et al. (2019). Dietary Intake of Whole Strawberry Inhibited Colonic Inflammation in Dextran-Sulfate-Sodium-Treated Mice via Restoring Immune Homeostasis and Alleviating Gut Microbiota Dysbiosis. *J. Agric. Food Chem.* 67, 9168–9177. doi: 10.1021/acs.jafc.8b05581

Hayouni, E. A., Chraief, I., Abedrabba, M., Bouix, M., Leveau, J.-Y., Mohammed, H., et al. (2008). Tunisian Salvia officinalis L. and Schinus molle L. essential oils: Their chemical compositions and their preservative effects against Salmonella inoculated in minced beef meat. *Int. J. Food Microbiol.* 125, 242–251. doi: https://doi.org/10.1016/j.ijfoodmicro.2008.04.005

Hosni, K., Jemli, M., Dziri, S., M’rabet, Y., Ennigrou, A., Sghaier, A., et al. (2011). Changes in phytochemical, antimicrobial and free radical scavenging activities of the Peruvian pepper tree (Schinus molle L.) as influenced by fruit maturation. *Ind. Crops Prod.* 34, 1622–1628. doi: https://doi.org/10.1016/j.indcrop.2011.06.004

Huaman, Y., de la Cruz, O. A., Bosilcov, A., and Batiu, I. (2004). Essential oil from the fruits of Schinus molle L. from Peru. *J. Essent. Oil Bear. Plants* 7, 223–227. doi: 10.1080/0972-060X.2004.10643396

Jiménez-Aspee, F., Theoduloz, C., Ávila, F., Thomas-Valdés, S., Mardones, C., von Baer, D., et al. (2016a). The Chilean wild raspberry (Rubus geoides Sm.) increases intracellular GSH content and protects against H2O2 and methylglyoxal-induced damage in AGS cells. *Food Chem.* 194, 908–919. doi: https://doi.org/10.1016/j.foodchem.2015.08.117

Jiménez-Aspee, F., Theoduloz, C., Pormetter, L., Mettke, J., Ávila, F., and Schmeda-Hirschmann, G. (2019). Andean Prumnopitys Andina (Podocarpacae) Fruit Extracts: Characterization of Secondary Metabolites and Potential Cytoprotective Effect. *Molecules* 24. doi: 10.3390/molecules24224028

Jiménez-Aspee, F., Theoduloz, C., Soriano, M. D. P. C., Ugalde-Arbizu, M., Alberto, M. R., Zampini, I. C., et al. (2017). The Native Fruit Geoffroea decorticans from Arid Northern Chile: Phenolic Composition, Antioxidant Activities and In Vitro Inhibition of Pro-Inflammatory and Metabolic Syndrome-Associated Enzymes. *Molecules* 22. doi: 10.3390/molecules22091565

Jiménez-Aspee, F., Thomas-Valdés, S., Schulz, A., Ladio, A., Theoduloz, C., and Schmeda-Hirschmann, G. (2016b). Antioxidant activity and phenolic profiles of the wild currant Ribes magellanicum from Chilean and Argentinean Patagonia. *Food Sci. Nutr.* 4, 595–610. doi: https://doi.org/10.1002/fsn3.323

Jofré, I., Cuevas, M., de Castro, L. S., de Agostini Losano, J. D., Torres, M. A., Alvear, M., et al. (2019). Antioxidant Effect of a Polyphenol-Rich Murtilla (*Ugni molinae* Turcz.) Extract and Its Effect on the Regulation of Metabolism in Refrigerated Boar Sperm. *Oxid. Med. Cell. Longev.* 2019, 2917513. doi: 10.1155/2019/2917513

Jofré, I., Pezoa, C., Cuevas, M., Scheuermann, E., Freires, I. A., Rosalen, P. L., et al. (2016). Antioxidant and Vasodilator Activity of *Ugni molinae* Turcz. (Murtilla) and Its Modulatory Mechanism in Hypotensive Response. *Oxid. Med. Cell. Longev.* 2016, 6513416. doi: 10.1155/2016/6513416

Junqueira-Gonçalves, M. P., Yáñez, L., Morales, C., Navarro, M., Contreras, R. A., and Zúñiga, G. E. (2015). Isolation and Characterization of Phenolic Compounds and Anthocyanins from Murta (Ugni molinae Turcz.) Fruits. Assessment of Antioxidant and Antibacterial Activity. *Molecules* 20, 5698–5713. doi: 10.3390/molecules20045698

Kasmi, A., Hammami, M., Raoelison, E. G., Abderrabba, M., Bouajila, J., and Ducamp, C. (2017). Chemical Composition and Behavioral Effects of Five Plant Essential Oils on the Green Pea Aphid Acyrthosiphon pisum (Harris) (Homoptera: Aphididae). *Chem. Biodivers.* 14, e1600464. doi: https://doi.org/10.1002/cbdv.201600464

Kim, H. W., Wang, M., Leber, C. A., Nothias, L.-F., Reher, R., Kang, K. Bin, et al. (2021a). NPClassifier: A Deep Neural Network-Based Structural Classification Tool for Natural Products. *J. Nat. Prod.* 84, 2795–2807. doi: 10.1021/acs.jnatprod.1c00399

Kim, M. J., Kim, D. W., Kim, J. G., Shin, Y., Jung, S. K., and Kim, Y.-J. (2021b). Analysis of the Chemical, Antioxidant, and Anti-Inflammatory Properties of Pink Pepper (Schinus molle L.). *Antioxidants* 10. doi: 10.3390/antiox10071062

Lamarque, A. L., Maestri, D. M., Grosso, N. R., Zygadlo, J. A., and Guzmán, C. A. (1994). Proximate composition and seed lipid components of some Prosopis (Leguminosae) from Argentina. *J. Sci. Food Agric.* 66, 323–326. doi: https://doi.org/10.1002/jsfa.2740660309

Lamarque, A. L., Maestri, D. M., Zygadlo, J. A., and Guzmán, C. A. (2000). Chemical evaluation of Geoffroea decorticans seeds as source of oil and protein. *Grasas y Aceites* 51, 241–243. doi: 10.3989/gya.2000.v51.i4.418

Li, J., Yuan, C., Pan, L., Benatrehina, P. A., Chai, H., Keller, W. J., et al. (2017). Bioassay-Guided Isolation of Antioxidant and Cytoprotective Constituents from a Maqui Berry (Aristotelia chilensis) Dietary Supplement Ingredient As Markers for Qualitative and Quantitative Analysis. *J. Agric. Food Chem.* 65, 8634–8642. doi: 10.1021/acs.jafc.7b03261

Lila, M. A., Ribnicky, D. M., Rojo, L. E., Rojas-Silva, P., Oren, A., Havenaar, R., et al. (2012). Complementary Approaches To Gauge the Bioavailability and Distribution of Ingested Berry Polyphenolics. *J. Agric. Food Chem.* 60, 5763–5771. doi: 10.1021/jf203526h

López de Dicastillo, C., Bustos, F., Valenzuela, X., López-Carballo, G., Vilariño, J. M., and Galotto, M. J. (2017). Chilean berry Ugni molinae Turcz. fruit and leaves extracts with interesting antioxidant, antimicrobial and tyrosinase inhibitory properties. *Food Res. Int.* 102, 119–128. doi: https://doi.org/10.1016/j.foodres.2017.09.073

López, J., Shun Ah-Hen, K., Vega-Gálvez, A., Morales, A., García-Segovia, P., and Uribe, E. (2017a). Effects of drying methods on quality attributes of murta (Ugni molinae turcz) berries: bioactivity, nutritional aspects, texture profile, microstructure and functional properties. *J. Food Process Eng.* 40, e12511. doi: https://doi.org/10.1111/jfpe.12511

López, J., Vega-Gálvez, A., Bilbao-Sainz, C., Chiou, B.-S., Uribe, E., and Quispe-Fuentes, I. (2017b). Influence of vacuum drying temperature on: Physico-chemical composition and antioxidant properties of murta berries. *J. Food Process Eng.* 40, e12569. doi: https://doi.org/10.1111/jfpe.12569

López, J., Vega-Gálvez, A., Rodríguez, A., Stucken, K., Barraza, C., and Aguilera, L. E. (2019). Relationship between antimicrobial activity, phenolic profile and antioxidant capacity of murta ( Ugni molinae Turcz) extracts prepared by different drying methods. *J. Berry Res.* 9, 587–601. doi: 10.3233/JBR-190403

López, M. D., Baenas, N., Retamal-Salgado, J., Zapata, N., and Moreno, D. A. (2018). Underutilized Native Biobío Berries: Opportunities for Foods and Trade. *Nat. Prod. Commun.* 13, 1934578X1801301226. doi: 10.1177/1934578X1801301226

Lucas-Gonzalez, R., Navarro-Coves, S., Pérez-Álvarez, J. A., Fernández-López, J., Muñoz, L. A., and Viuda-Martos, M. (2016). Assessment of polyphenolic profile stability and changes in the antioxidant potential of maqui berry (Aristotelia chilensis (Molina) Stuntz) during in vitro gastrointestinal digestion. *Ind. Crops Prod.* 94, 774–782. doi: https://doi.org/10.1016/j.indcrop.2016.09.057

Madrid, A., Espinoza, L., Mellado, M., Montenegro, I., Gonzalez, C., Santander, R., et al. (2013). Study of the chemical composition of the resinous exudate isolated from Psoralea glandulosa and evaluation of the antioxidant properties of the terpenoids and the resin. *Boletín Latinoam. y del Caribe Plantas Med. y Aromáticas* 12, 338–345.

Maestri, D. M., Fortunato, R. H., Guzmán, C. A., Torres, M. M., and Lamarque, A. L. (2002). Seed compositional studies of some species of Papilionoideae (Leguminosae) native to Argentina. *J. Sci. Food Agric.* 82, 248–251. doi: https://doi.org/10.1002/jsfa.1022

Maffei, M., and Chialva, F. (1990). Essential oils from Schinus molle L. berries and leaves. *Flavour Fragr. J.* 5, 49–52. doi: https://doi.org/10.1002/ffj.2730050109

Martins, M. do R., Arantes, S., Candeias, F., Tinoco, M. T., and Cruz-Morais, J. (2014). Antioxidant, antimicrobial and toxicological properties of Schinus molle L. essential oils. *J. Ethnopharmacol.* 151, 485–492. doi: https://doi.org/10.1016/j.jep.2013.10.063

Medrano, M. A., Tomas, M. A., and Frontera, M. A. (1985). Isolation and identification of anthocyanins in fruits from Chubut Province (Argentina). Fruits of Ribes aureum Pursh, R. magellanicum Poir and Berberis darwinii Hook. *Rev. Latinoam. Química* 16, 84–86.

Mieres-Castro, D., Schmeda-Hirschmann, G., Theoduloz, C., Gómez-Alonso, S., Pérez-Navarro, J., Márquez, K., et al. (2019). Antioxidant activity and the isolation of polyphenols and new iridoids from Chilean Gaultheria phillyreifolia and G. poeppigii berries. *Food Chem.* 291, 167–179. doi: https://doi.org/10.1016/j.foodchem.2019.04.019

Mieres-Castro, D., Theoduloz, C., Sus, N., Burgos-Edwards, A., Schmeda-Hirschmann, G., Frank, J., et al. (2022). Iridoids and polyphenols from chilean Gaultheria spp. berries decrease the glucose uptake in Caco-2 cells after simulated gastrointestinal digestion. *Food Chem.* 369, 130940. doi: https://doi.org/10.1016/j.foodchem.2021.130940

Muñoz, C., Sánchez-Sevilla, J. F., Botella, M. A., Hoffmann, T., Schwab, W., and Valpuesta, V. (2011). Polyphenol Composition in the Ripe Fruits of Fragaria Species and Transcriptional Analyses of Key Genes in the Pathway. *J. Agric. Food Chem.* 59, 12598–12604. doi: 10.1021/jf203965j

Noriega, F., Mardones, C., Fischer, S., García-Viguera, C., Moreno, D. A., and López, M. D. (2021). Seasonal changes in white strawberry: Effect on aroma, phenolic compounds and its biological activity. *J. Berry Res.* 11, 103–118. doi: 10.3233/JBR-200585

Nowak, D., Gośliński, M., Przygoński, K., and Wojtowicz, E. (2018). The antioxidant properties of exotic fruit juices from acai, maqui berry and noni berries. *Eur. Food Res. Technol.* 244, 1897–1905. doi: 10.1007/s00217-018-3102-8

Olivares-Caro, L., Radojkovic, C., Chau, S. Y., Nova, D., Bustamante, L., Neira, J. Y., et al. (2020). Berberis microphylla G. Forst (Calafate) Berry Extract Reduces Oxidative Stress and Lipid Peroxidation of Human LDL. *Antioxidants* 9. doi: 10.3390/antiox9121171

Ono, M., Yamashita, M., Mori, K., Masuoka, C., Eto, M., Kinjo, J., et al. (2008). Sesquiterpenoids, Triterpenoids, and Flavonoids from the Fruits of Schinus molle. *Food Sci. Technol. Res.* 14, 499. doi: 10.3136/fstr.14.499

Osman, E. E. A., Morsi, E. A., El-Sayed, M. M., Gobouri, A., and Abdel-Hameed, E. S. S. (2021). Identification of the volatile and nonvolatile constituents of Schinus molle (L.) fruit extracts and estimation of their activities as anticancer agents. *J. Appl. Pharm. Sci.* 11, 163–171. doi: 10.7324/JAPS.2021.110719

Otero, C., Miranda-Rojas, S., Llancalahuén, F. M., Fuentes, J. A., Atala, C., González-Silva, G., et al. (2022). Biochemical characterization of Peumus boldus fruits: Insights of its antioxidant properties through a theoretical approach. *Food Chem.* 370, 131012. doi: https://doi.org/10.1016/j.foodchem.2021.131012

Overall, J., Bonney, S. A., Wilson, M., Beermann, A., Grace, M. H., Esposito, D., et al. (2017). Metabolic Effects of Berries with Structurally Diverse Anthocyanins. *Int. J. Mol. Sci.* 18. doi: 10.3390/ijms18020422

Oyarzún, P., Cornejo, P., Gómez-Alonso, S., and Ruiz, A. (2020). Influence of Profiles and Concentrations of Phenolic Compounds in the Coloration and Antioxidant Properties of Gaultheria poeppigii Fruits from Southern Chile. *Plant Foods Hum. Nutr.* 75, 532–539. doi: 10.1007/s11130-020-00843-x

Peçanha, J. de S., Santos, N. M. dos, Maróstica Júnior, M. R., Micheletti, A. C., Lião, L. M., and Alcantara, G. B. (2022). NMR-based metabolomics of dried berries in comparison with dietary supplements. *J. Pharm. Biomed. Anal.* 209, 114494. doi: https://doi.org/10.1016/j.jpba.2021.114494

Peñarrieta, J. M., Alvarado, J. A., Åkesson, B., and Bergenståhl, B. (2008). Total antioxidant capacity and content of flavonoids and other phenolic compounds in canihua (Chenopodium pallidicaule): An Andean pseudocereal. *Mol. Nutr. Food Res.* 52, 708–717. doi: https://doi.org/10.1002/mnfr.200700189

Pérez-Arancibia, R., Ordoñez, J. L., Rivas, A., Pihán, P., Sagredo, A., Ahumada, U., et al. (2021). A phenolic-rich extract from Ugni molinae berries reduces abnormal protein aggregation in a cellular model of Huntington’s disease. *PLoS One* 16, e0254834. Available at: https://doi.org/10.1371/journal.pone.0254834

Pérez-López, A., Cirio, A. T., Rivas-Galindo, V. M., Aranda, R. S., and de Torres, N. W. (2011). Activity against Streptococcus pneumoniae of the Essential Oil and δ-Cadinene Isolated from Schinus molle Fruit. *J. Essent. Oil Res.* 23, 25–28. doi: 10.1080/10412905.2011.9700477

Pérez, M. J., Cuello, A. S., Zampini, I. C., Ordoñez, R. M., Alberto, M. R., Quispe, C., et al. (2014). Polyphenolic compounds and anthocyanin content of Prosopis nigra and Prosopis alba pods flour and their antioxidant and anti-inflammatory capacities. *Food Res. Int.* 64, 762–771. doi: https://doi.org/10.1016/j.foodres.2014.08.013

Picariello, G., Sciammaro, L., Siano, F., Volpe, M. G., Puppo, M. C., and Mamone, G. (2017). Comparative analysis of C-glycosidic flavonoids from Prosopis spp. and Ceratonia siliqua seed germ flour. *Food Res. Int.* 99, 730–738. doi: https://doi.org/10.1016/j.foodres.2017.06.058

Pineda, A., Arenas, A., Balmaceda, J., and Zúñiga, G. E. (2022). Extracts of Fruits and Plants Cultivated In Vitro of Aristotelia chilensis (Mol.) Stuntz Show Inhibitory Activity of Aldose Reductase and Pancreatic Alpha-Amylase Enzymes. *Plants* 11. doi: 10.3390/plants11202772

Pinto, A. A., Fuentealba-Sandoval, V., López, M. D., Peña-Rojas, K., and Fischer, S. (2022). Accumulation of delphinidin derivatives and other bioactive compound in wild maqui under different environmental conditions and fruit ripening stages. *Ind. Crops Prod.* 184, 115064. doi: https://doi.org/10.1016/j.indcrop.2022.115064

Pozzo-Balbi, T., Nobile, L., Scapini, G., and Cini, M. (1978). The triterpenoid acids of Schinus molle. *Phytochemistry* 17, 2107–2110. doi: https://doi.org/10.1016/S0031-9422(00)89290-0

Prat, L., Espinoza, M. I., Agosin, E., and Silva, H. (2014). Identification of volatile compounds associated with the aroma of white strawberries (Fragaria chiloensis). *J. Sci. Food Agric.* 94, 752–759. doi: https://doi.org/10.1002/jsfa.6412

Quispe-Fuentes, I., Vega-Gálvez, A., and Aranda, M. (2018). Evaluation of phenolic profiles and antioxidant capacity of maqui (Aristotelia chilensis) berries and their relationships to drying methods. *J. Sci. Food Agric.* 98, 4168–4176. doi: https://doi.org/10.1002/jsfa.8938

Quispe-Fuentes, I., Vega-Gálvez, A., Uribe, E., Vásquez, V., Cárdenas, N., and Poblete, J. (2019). Vacuum drying application to maqui (Aristotelia chilensis [Mol] Stuntz) berry: Weibull distribution for process modelling and quality parameters. *J. Food Sci. Technol.* 56, 1899–1908. doi: 10.1007/s13197-019-03653-5

Rajaram, N., and Janardhanan, K. (1991). Studies on the underexploited tree pulses, Acacia catechu willd., Parkinsonia aculeata L. and Prosopis chilensis (molina) stunz: Chemical composition and antinutritional factors. *Food Chem.* 42, 265–273. doi: https://doi.org/10.1016/0308-8146(91)90069-Z

Ramirez, J. E., Zambrano, R., Sepúlveda, B., Kennelly, E. J., and Simirgiotis, M. J. (2015). Anthocyanins and antioxidant capacities of six Chilean berries by HPLC–HR-ESI-ToF-MS. *Food Chem.* 176, 106–114. doi: https://doi.org/10.1016/j.foodchem.2014.12.039

Ramirez, L. A., Quezada, J., Duarte, L., Concha, F., Escobillana, L., Rincon-Cervera, M. A., et al. (2021). The administration of an extract from Berberis microphylla stimulates energy expenditure, thermogenesis and mitochondrial dynamics in mice brown adipose tissue. *Food Biosci.* 41, 100988. doi: https://doi.org/10.1016/j.fbio.2021.100988

Rastrelli, L., De Simone, F., Schettino, O., and Dini, A. (1996a). Constituents of Chenopodium pallidicaule (Cañihua) Seeds:  Isolation and Characterization of New Triterpene Saponins. *J. Agric. Food Chem.* 44, 3528–3533. doi: 10.1021/jf950253p

Rastrelli, L., de Tommasi, N., and Ramos, I. (1996b). Ecdysteroids in Chenopodium pallidicaule seeds. *Biochem. Syst. Ecol.* 24, 353. doi: https://doi.org/10.1016/0305-1978(96)00019-1

Rastrelli, L., Saturnino, P., Schettino, O., and Dini, A. (1995). Studies on the Constituents of Chenopodium pallidicaule (Canihua) Seeds. Isolation and Characterization of Two New Flavonol Glycosides. *J. Agric. Food Chem.* 43, 2020–2024. doi: 10.1021/jf00056a012

Repo-Carrasco-Valencia, R., Hellström, J. K., Pihlava, J.-M., and Mattila, P. H. (2010). Flavonoids and other phenolic compounds in Andean indigenous grains: Quinoa (Chenopodium quinoa), kañiwa (Chenopodium pallidicaule) and kiwicha (Amaranthus caudatus). *Food Chem.* 120, 128–133. doi: https://doi.org/10.1016/j.foodchem.2009.09.087

Rey-Valeirón, C., Guzmán, L., Saa, L. R., López-Vargas, J., and Valarezo, E. (2017). Acaricidal activity of essential oils of Bursera graveolens (Kunth) Triana & Planch and Schinus molle L. on unengorged larvae of cattle tick Rhipicephalus (Boophilus) microplus (Acari:Ixodidae). *J. Essent. Oil Res.* 29, 344–350. doi: 10.1080/10412905.2016.1278405

Reyes-Farias, M., Vasquez, K., Fuentes, F., Ovalle-Marin, A., Parra-Ruiz, C., Zamora, O., et al. (2016). Extracts of Chilean native fruits inhibit oxidative stress, inflammation and insulin-resistance linked to the pathogenic interaction between adipocytes and macrophages. *J. Funct. Foods* 27, 69–83. doi: https://doi.org/10.1016/j.jff.2016.08.052

Reyes-Farias, M., Vasquez, K., Ovalle-Marin, A., Fuentes, F., Parra, C., Quitral, V., et al. (2014). Chilean Native Fruit Extracts Inhibit Inflammation Linked to the Pathogenic Interaction Between Adipocytes and Macrophages. *J. Med. Food* 18, 601–608. doi: 10.1089/jmf.2014.0031

Rocha, P. M. de M., Rodilla, J. M., Díez, D., Elder, H., Guala, M. S., Silva, L. A., et al. (2012). Synergistic Antibacterial Activity of the Essential Oil of Aguaribay (Schinus molle L.). *Molecules* 17, 12023–12036. doi: 10.3390/molecules171012023

Rodríguez, I. F., Cattaneo, F., Zech, X. V., Svavh, E., Pérez, M. J., Zampini, I. C., et al. (2020). Aloja and añapa, two traditional beverages obtained from Prosopis alba pods: Nutritional and functional characterization. *Food Biosci.* 35, 100546. doi: https://doi.org/10.1016/j.fbio.2020.100546

Rodriguez, I. F., Pérez, M. J., Cattaneo, F., Zampini, I. C., Cuello, A. S., Mercado, M. I., et al. (2019). Morphological, histological, chemical and functional characterization of Prosopis alba flours of different particle sizes. *Food Chem.* 274, 583–591. doi: https://doi.org/10.1016/j.foodchem.2018.09.024

Rodríguez, K., Ah-Hen, K. S., Vega-Gálvez, A., Vásquez, V., Quispe-Fuentes, I., Rojas, P., et al. (2016). Changes in bioactive components and antioxidant capacity of maqui, Aristotelia chilensis [Mol] Stuntz, berries during drying. *LWT - Food Sci. Technol.* 65, 537–542. doi: https://doi.org/10.1016/j.lwt.2015.08.050

Rodríguez, L., Trostchansky, A., Wood, I., Mastrogiovanni, M., Vogel, H., González, B., et al. (2021). Antiplatelet activity and chemical analysis of leaf and fruit extracts from Aristotelia chilensis. *PLoS One* 16, e0250852. Available at: https://doi.org/10.1371/journal.pone.0250852

Rojo, L. E., Ribnicky, D., Logendra, S., Poulev, A., Rojas-Silva, P., Kuhn, P., et al. (2012). In vitro and in vivo anti-diabetic effects of anthocyanins from Maqui Berry (Aristotelia chilensis). *Food Chem.* 131, 387–396. doi: https://doi.org/10.1016/j.foodchem.2011.08.066

Roldán, C. S., Caballé, G., Fontana, A., Viale, M., and Berli, F. (2021). Maqui (Aristotelia chilensis [Mol.] Stuntz) morphological and phenolic traits associated with forests type and latitudinal gradient in natural populations of Patagonia Argentina. *J. Appl. Res. Med. Aromat. Plants* 25, 100341. doi: https://doi.org/10.1016/j.jarmap.2021.100341

Romero-Román, M. E., Schoebitz, M., Bastías, R. M., Fernández, P. S., García-Viguera, C., and López-Belchi, M. D. (2021a). Native Species Facing Climate Changes: Response of Calafate Berries to Low Temperature and UV Radiation. *Foods* 10. doi: 10.3390/foods10010196

Romero-Román, M. E., Schoebitz, M., Fuentealba, J., García-Viguera, C., and Belchí, M. D. L. (2021b). Phenolic Compounds in Calafate Berries Encapsulated by Spray Drying: Neuroprotection Potential into the Ingredient. *Antioxidants* 10. doi: 10.3390/antiox10111830

Rubilar, M., Jara, C., Poo, Y., Acevedo, F., Gutierrez, C., Sineiro, J., et al. (2011). Extracts of Maqui (Aristotelia chilensis) and Murta (Ugni molinae Turcz.): Sources of Antioxidant Compounds and α-Glucosidase/α-Amylase Inhibitors. *J. Agric. Food Chem.* 59, 1630–1637. doi: 10.1021/jf103461k

Ruiz, A., Bustamante, L., Vergara, C., von Baer, D., Hermosín-Gutiérrez, I., Obando, L., et al. (2015). Hydroxycinnamic acids and flavonols in native edible berries of South Patagonia. *Food Chem.* 167, 84–90. doi: https://doi.org/10.1016/j.foodchem.2014.06.052

Ruiz, A., Hermosín-Gutiérrez, I., Mardones, C., Vergara, C., Herlitz, E., Vega, M., et al. (2010). Polyphenols and Antioxidant Activity of Calafate (Berberis microphylla) Fruits and Other Native Berries from Southern Chile. *J. Agric. Food Chem.* 58, 6081–6089. doi: 10.1021/jf100173x

Ruiz, A., Hermosín-Gutiérrez, I., Vergara, C., von Baer, D., Zapata, M., Hitschfeld, A., et al. (2013a). Anthocyanin profiles in south Patagonian wild berries by HPLC-DAD-ESI-MS/MS. *Food Res. Int.* 51, 706–713. doi: https://doi.org/10.1016/j.foodres.2013.01.043

Ruiz, A., Mardones, C., Vergara, C., Hermosín-Gutiérrez, I., von Baer, D., Hinrichsen, P., et al. (2013b). Analysis of hydroxycinnamic acids derivatives in calafate (Berberis microphylla G. Forst) berries by liquid chromatography with photodiode array and mass spectrometry detection. *J. Chromatogr. A* 1281, 38–45. doi: https://doi.org/10.1016/j.chroma.2013.01.059

Ruiz, A., Mardones, C., Vergara, C., von Baer, D., Gómez-Alonso, S., Gómez, M. V., et al. (2014a). Isolation and Structural Elucidation of Anthocyanidin 3,7-β-O-Diglucosides and Caffeoyl-glucaric Acids from Calafate Berries. *J. Agric. Food Chem.* 62, 6918–6925. doi: 10.1021/jf5012825

Ruiz, A., Pastene, E., Vergara, C., von Baer, D., Avello, M., and Mardones, C. (2016). Hydroxycinnamic acid derivatives and flavonol profiles of maqui (Aristotelia chilensis) fruits. *J. Chil. Chem. Soc.* 61, 2792–2796.

Ruiz, A., Zapata, M., Sabando, C., Bustamante, L., von Baer, D., Vergara, C., et al. (2014b). Flavonols, Alkaloids, and Antioxidant Capacity of Edible Wild Berberis Species from Patagonia. *J. Agric. Food Chem.* 62, 12407–12417. doi: 10.1021/jf502929z

Salazar, C., Bustos, E., Perez, C., Becerra, J., Bru, R., and Uribe, M. (2017). Callus Induction and Cellular Suspensions from Murtilla (Ugni molinae Turcz.) for trans-resveratrol Production. *Am. J. Biochem. Biotechnol.* 13. doi: 10.3844/ajbbsp.2017.7.14

Salvatierra, A., Pimentel, P., Moya-Leon, M. A., Caligari, P. D. S., and Herrera, R. (2010). Comparison of transcriptional profiles of flavonoid genes and anthocyanin contents during fruit development of two botanical forms of Fragaria chiloensis ssp. chiloensis. *Phytochemistry* 71, 1839–1847. doi: https://doi.org/10.1016/j.phytochem.2010.08.005

Salvatierra, A., Pimentel, P., Moya-León, M. A., and Herrera, R. (2013). Increased accumulation of anthocyanins in Fragaria chiloensis fruits by transient suppression of FcMYB1 gene. *Phytochemistry* 90, 25–36. doi: https://doi.org/10.1016/j.phytochem.2013.02.016

Sandoval, V., Femenias, A., Martínez-Garza, Ú., Sanz-Lamora, H., Castagnini, J. M., Quifer-Rada, P., et al. (2019). Lyophilized Maqui (Aristotelia chilensis) Berry Induces Browning in the Subcutaneous White Adipose Tissue and Ameliorates the Insulin Resistance in High Fat Diet-Induced Obese Mice. *Antioxidants* 8. doi: 10.3390/antiox8090360

Scheuermann, E., Seguel, I., Montenegro, A., Bustos, R. O., Hormazábal, E., and Quiroz, A. (2008). Evolution of aroma compounds of murtilla fruits (Ugni molinae Turcz) during storage. *J. Sci. Food Agric.* 88, 485–492. doi: https://doi.org/10.1002/jsfa.3111

Schmeda-Hirschmann, G., Antileo-Laurie, J., Theoduloz, C., Jiménez-Aspee, F., Avila, F., Burgos-Edwards, A., et al. (2021). Phenolic composition, antioxidant capacity and α-glucosidase inhibitory activity of raw and boiled Chilean Araucaria araucana kernels. *Food Chem.* 350, 129241. doi: https://doi.org/10.1016/j.foodchem.2021.129241

Schmeda-Hirschmann, G., Quispe, C., Soriano, M. D. P. C., Theoduloz, C., Jiménez-Aspée, F., Pérez, M. J., et al. (2015). Chilean Prosopis Mesocarp Flour: Phenolic Profiling and Antioxidant Activity. *Molecules* 20, 7017–7033. doi: 10.3390/molecules20047017

Schreckinger, M. E., Wang, J., Yousef, G., Lila, M. A., and Gonzalez de Mejia, E. (2010). Antioxidant Capacity and in Vitro Inhibition of Adipogenesis and Inflammation by Phenolic Extracts of Vaccinium floribundum and Aristotelia chilensis. *J. Agric. Food Chem.* 58, 8966–8976. doi: 10.1021/jf100975m

Schreckinger, M., Lila, M. A., Yousef, G., and de Mejia, E. (2012). “Inhibition of α-Glucosidase and α-Amylase by Vaccinium floribundum and Aristotelia chilensis Proanthocyanidins,” in *Hispanic Foods: Chemistry and Bioactive Compounds*, (American Chemical Society), 6–71. doi: doi:10.1021/bk-2012-1109.ch006

Silva, R., de Ruiz, R. E. L., and Ruiz, S. O. (1999). Estudio fitoquímico de frutos de Geoffroea decorticans (Gill ex Hook. et Arn.) Burk. Leguminosae (Fabaceae). *Acta Farm. Bonaer.* 18, 217–219.

Simirgiotis, M. J., Bórquez, J., and Schmeda-Hirschmann, G. (2013). Antioxidant capacity, polyphenolic content and tandem HPLC–DAD–ESI/MS profiling of phenolic compounds from the South American berries Luma apiculata and L. chequén. *Food Chem.* 139, 289–299. doi: https://doi.org/10.1016/j.foodchem.2013.01.089

Simirgiotis, M. J., and Schmeda-Hirschmann, G. (2010). Determination of phenolic composition and antioxidant activity in fruits, rhizomes and leaves of the white strawberry (Fragaria chiloensis spp. chiloensis form chiloensis) using HPLC-DAD–ESI-MS and free radical quenching techniques. *J. Food Compos. Anal.* 23, 545–553. doi: https://doi.org/10.1016/j.jfca.2009.08.020

Simirgiotis, M. J., Theoduloz, C., Caligari, P. D. S., and Schmeda-Hirschmann, G. (2009). Comparison of phenolic composition and antioxidant properties of two native Chilean and one domestic strawberry genotypes. *Food Chem.* 113, 377–385. doi: https://doi.org/10.1016/j.foodchem.2008.07.043

Soto-Covasich, J., Reyes-Farias, M., Torres, R. F., Vasquez, K., Duarte, L., Quezada, J., et al. (2020). A polyphenol-rich Calafate (Berberis microphylla) extract rescues glucose tolerance in mice fed with cafeteria diet. *J. Funct. Foods* 67, 103856. doi: https://doi.org/10.1016/j.jff.2020.103856

Takeoka, G., Felker, P., Prokopiuk, D., and Dao, L. (2008). “Volatile Constituents of Mesquite (<italic>Prosopis</italic>) Pods,” in *Food Flavor*, (American Chemical Society), 9–98. doi: doi:10.1021/bk-2008-0988.ch009

Tanaka, J., Kadekaru, T., Ogawa, K., Hitoe, S., Shimoda, H., and Hara, H. (2013). Maqui berry (Aristotelia chilensis) and the constituent delphinidin glycoside inhibit photoreceptor cell death induced by visible light. *Food Chem.* 139, 129–137. doi: https://doi.org/10.1016/j.foodchem.2013.01.036

Terhune, S. J., Hogg, J. W., and Lawrence, B. M. (1974). β-Spathulene: A new sesquiterpene in Schinus molle oil. *Phytochemistry* 13, 865–866. doi: https://doi.org/10.1016/S0031-9422(00)91153-1

Theoduloz, C., Burgos-Edwards, A., Schmeda-Hirschmann, G., and Jiménez-Aspee, F. (2018). Effect of polyphenols from wild Chilean currants (Ribes spp.) on the activity of intracellular antioxidant enzymes in human gastric AGS cells. *Food Biosci.* 24, 80–88. doi: https://doi.org/10.1016/j.fbio.2018.06.003

Thomas-Valdés, S., Theoduloz, C., Jiménez-Aspee, F., Burgos-Edwards, A., and Schmeda-Hirschmann, G. (2018). Changes in polyphenol composition and bioactivity of the native Chilean white strawberry (Fragaria chiloensis spp. chiloensis f. chiloensis) after in vitro gastrointestinal digestion. *Food Res. Int.* 105, 10–18. doi: https://doi.org/10.1016/j.foodres.2017.10.074

Thomas-Valdés, S., Theoduloz, C., Jiménez-Aspee, F., and Schmeda-Hirschmann, G. (2019). Effect of simulated gastrointestinal digestion on polyphenols and bioactivity of the native Chilean red strawberry (Fragaria chiloensis ssp. chiloensis f. patagonica). *Food Res. Int.* 123, 106–114. doi: https://doi.org/10.1016/j.foodres.2019.04.039

Tlili, N., Yahia, Y., Feriani, A., Labidi, A., Ghazouani, L., Nasri, N., et al. (2018). Schinus terebinthifolius vs Schinus molle: A comparative study of the effect of species and location on the phytochemical content of fruits. *Ind. Crops Prod.* 122, 559–565. doi: https://doi.org/10.1016/j.indcrop.2018.05.080

Vázquez-Espinosa, M., Espada-Bellido, E., V. González de Peredo, A., Ferreiro-González, M., Carrera, C., Palma, M., et al. (2018). Optimization of Microwave-Assisted Extraction for the Recovery of Bioactive Compounds from the Chilean Superfruit (Aristotelia chilensis (Mol.) Stuntz). *Agronomy* 8. doi: 10.3390/agronomy8110240

Vázquez-Espinosa, M., V. González de Peredo, A., Ferreiro-González, M., Carrera, C., Palma, M., F. Barbero, G., et al. (2019). Assessment of Ultrasound Assisted Extraction as an Alternative Method for the Extraction of Anthocyanins and Total Phenolic Compounds from Maqui Berries (Aristotelia chilensis (Mol.) Stuntz). *Agronomy* 9. doi: 10.3390/agronomy9030148

Velásquez, P., Orellana, J., Muñoz-Carvajal, E., Faúndez, M., Gómez, M., Montenegro, G., et al. (2022). Biological activity of native Myrtaceae fruits from Chile as a potential functional food. *Nat. Prod. Res.* 36, 3138–3142. doi: 10.1080/14786419.2021.1940176

Velásquez, P., Sandoval, M. I., Giordano, A., Górmez, M., and Montenegro, G. (2017). Nutritional Composition and Polyphenolic Content of Edible Peumus boldus Mol. Fruits. *Cienc. e Investig. Agrar.* 44, 54–63. doi: https://dx.doi.org/10.7764/rcia.v44i1.1684

Viktorová, J., Kumar, R., Řehořová, K., Hoang, L., Ruml, T., Figueroa, C. R., et al. (2020). Antimicrobial Activity of Extracts of Two Native Fruits of Chile: Arrayan (Luma apiculata) and Peumo (Cryptocarya alba). *Antibiotics* 9. doi: 10.3390/antibiotics9080444

Viuda-Martos, M., Lucas-Gonzalez, R., Ballester-Costa, C., Pérez-Álvarez, J. A., Muñoz, L. A., and Fernández-López, J. (2018). Evaluation of protective effect of different dietary fibers on polyphenolic profile stability of maqui berry (Aristotelia chilensis (Molina) Stuntz) during in vitro gastrointestinal digestion. *Food Funct.* 9, 573–584. doi: 10.1039/C7FO01671A

Volpini-Klein, A. F. N., Lima Júnior, S. E., Cardoso, C. A. L., Cabral, M. R. P., Louro, G. M., Coutinho, E. J., et al. (2021). Chemical Composition of Essential Oils from Leaves and Fruits of Schinus molle Obtained by Different Extraction Methods (Hydrodistillation, Fractional Hydrodistillation and Steam Distillation) and Seasonal Variations. *J. Essent. Oil Bear. Plants* 24, 228–242. doi: 10.1080/0972060X.2021.1914739

Volpini-Klein, A. F. N., Silva, C. A. A., Fernandes, S. S. L., Nicolau, C. L., Cardoso, C. A. L., Fiorucci, A. R., et al. (2020). Effect of leaf and fruit extracts of Schinus molle on oxidative stability of some vegetables oils under accelerated oxidation. *Grasas y Aceites* 71, e363. doi: 10.3989/gya.0456191

Wang, J. Z., Yousef, G. G., Rogers, R. B., Gonzalez de Mejia, E., Raskin, I., and Lila, M. A. (2012). “Maqui Berry (Aristotelia chilensis) Juices Fermented with Yeasts: Effects on Phenolic Composition, Antioxidant Capacity, and iNOS and COX-2 Protein Expression,” in *Emerging Trends in Dietary Components for Preventing and Combating Disease*, (American Chemical Society), 6–95. doi: doi:10.1021/bk-2012-1093.ch006

Wang, S. Y., and Lewers, K. S. (2007). Antioxidant Capacity and Flavonoid Content in Wild Strawberries. *J. Am. Soc. Hortic. Sci. J. Amer. Soc. Hort. Sci.* 132, 629–637. doi: 10.21273/JASHS.132.5.629

Young, J., Pesek, J., Matyska, M., Nguyen, T., Jarman, S., Diep, D., et al. (2017). LC–MS Characterization of Mesquite Flour Constituents. *LCGC Asia Pacific* 20, 6–9.

Yueqin, Z., Recio, M. C., Máñez, S., Giner, R. M., Cerdá-Nicolás, M., and Ríos, J.-L. (2003). Isolation of Two Triterpenoids and a Biflavanone with Anti-Inflammatory Activity from Schinus molle Fruits. *Planta Med* 69, 893–898. doi: 10.1055/s-2003-45096

Zahed, N., Hosni, K., Ben Brahim, N., Kallel, M., and Sebei, H. (2010). Allelopathic effect of Schinus molle essential oils on wheat germination. *Acta Physiol. Plant.* 32, 1221–1227. doi: 10.1007/s11738-010-0492-z

Zahed, N., Hosni, K., Brahim, N. B. E. N., and Sebei, H. (2011). Essential oil composition of Schinus molle L. fruits: an ornamental species used as condiment. *J. Food Biochem.* 35, 400–408. doi: https://doi.org/10.1111/j.1745-4514.2010.00391.x

Zhou, G., Chen, L., Sun, Q., Mo, Q.-G., Sun, W.-C., and Wang, Y.-W. (2019). Maqui berry exhibited therapeutic effects against DSS-induced ulcerative colitis in C57BL/6 mice. *Food Funct.* 10, 6655–6665. doi: 10.1039/C9FO00663J
