# Supplementary material for: Fermented beverages in prehispanic Chile: a comprehensive review of their phytochemistry, traditional medicinal uses, bioactivity, and social aspects
Source: Front Pharmacol. 2024 Nov 21;15:1505873. doi: 10.3389/fphar.2024.1505873 (PMC11617176; doi:10.3389/fphar.2024.1505873)
Supplement: Supplementary file 2 [file Table2.DOCX]

**Table S2.** Traditional medicinal uses reported for the prehispanic fermented beverages in Chile and for the raw materials used in their elaboration.

| **Species - Family** | **Traditional medicinal uses** | **References** |
| --- | --- | --- |
| *Alstroemeria ligtu* L. [Alstroemeriaceae] | **Rhizomes:** nutritive for children and patients in recovery, stomachic, against inflammations of the digestive tract, remedy against bacterial skin infections and other skin conditions | (Murillo, 1889; Espinoza, 1897; Muñoz S. et al., 1981; de Mösbach, 1992) |
| *Amomyrtus luma* (Molina) D.Legrand & Kausel [Myrtaceae] | **Fermented beverage:** stomachic  **Whole plant:** stimulant, astringent | (Muñoz S. et al., 1981; Molina, 1987; Rapoport et al., 2003a) |
| *Araucaria araucana* (Molina) K.Koch (syn. *Araucaria imbricata* Pav.) [Araucariaceae] | **Seeds:** galactagogue, aphrodisiac | (Murillo, 1889; Muñoz S. et al., 1981) |
| *Aristotelia chilensis* (Molina) Stuntz [Elaeocarpaceae] | **Fermented beverage:** astringent, tonic  **Fruits:** astringent, antidiarrheic, refrigerant, tonic, against dysentery, throat infections, mouth ulcers, wounds, remedy against hair loss – febrifuge, stomachic, against chronic diarrheas and dysentery (infusion) | (Murillo, 1889; Espinoza, 1897; Muñoz S. et al., 1981; Hoffmann et al., 1992; Rapoport et al., 2003a; Cárdenas Álvarez and Villagrán, 2005) |
| *Berberis spp.* [Berberidaceae] | **Fruits:** anti-inflammatory (infusion) | (Lenz, 1910) |
| *Berberis darwinii* Hook. [Berberidaceae] | **Fruits:** anti-inflammatory, febrifuge, mild laxative, refrigerant | (Rapoport et al., 2003a, 2003b) |
| *Berberis microphylla* G. Forst. (syn*. Berberis buxifolia* Lam*.*, *Berberis parodii* Job) [Berberidaceae] | **Fruits:** astringent, febrifuge, mild laxative, refrigerant, remedy against colds and flu | (de Mösbach, 1992; Rapoport et al., 2003a) |
| *Fragaria chiloensis* (L.) Mill (syn. *Potentilla chiloensis* (L.) Mabb.) [Rosaceae] | **Whole plant:** astringent, digestive, antihemorrhagic, antidiarrheic, diuretic, emollient, aperitive, against conditions affecting vision (herbal tea) | (de Mösbach, 1992; Rapoport et al., 2003a) |
| *Geoffroea decorticans* (Gillies ex Hook. & Arn.) Burkat [Fabaceae] | **Fruits:** emollient, expectorant – antitussive, remedy for respiratory infections, bronchitis and asthma (syrup of crushed fruits with water and burnt sugar) | (Muñoz S. et al., 1981) |
| *Greigia sphacelata* (Ruiz & Pav.) Regel [Bromeliaceae] | **Seeds:** cathartic | (Muñoz S. et al., 1981) |
| *Jubaea chilensis* (Molina) Baill. [Arecaceae] | **Trunk sap:** digestive, mild laxative | (Murillo, 1889) |
| *Luma apiculata* (DC.) Burret (syn. *Myrceugenella apiculata* (DC.) Kausel) [Myrtaceae] | **Fruits:** tonic  **Whole plant:** stimulant, astringent, vulnerary, antihemorrhagic, antidiarrhetic, against dysentery – stomachic (decoction) | (de Mösbach, 1992; Hoffmann et al., 1992) |
| *Muehlenbeckia hastulata* (Sm.) I.M. Johnst. [Polygonaceae] | **Fermented beverage:** refrigerant  **Whole plant:** laxative, antihypertensive | (Muñoz S. et al., 1981) |
| *Neltuma chilensis* (Molina) C.E.Hughes & G.P.Lewis (syn. *Prosopis chilensis* (Molina) Stuntz) [Fabaceae] | **Fermented beverage:** diuretic  **Fruits:** cicatrizant, antihemorrhagic (topic application of crushed fruit) – remedy for heart conditions (fruit pulp) | (Muñoz S. et al., 1981) |
| *Otholobium glandulosum* (L.) J.W.Grimes (syn. *Psoralea glandulosa* L.) [Fabaceae] | **Leaves:** carminative, digestive, stomachic, against anorexia, cholera, migraines (herbal tea, infusion) – febrifuge (crushed or powdered) - vulnerary (infusion and poultice), remedy against hemorrhoids (baths with decoction and topic exposure to steam), against intestinal infections caused by pinworms (used in form of poultice of crashed leaves mixed with urine)  **Whole plant:** antidiabetic, digestive, emollient, sudorific, vulnerary, remedy against ulcers and fractures (decoction) - against intestinal infection caused by pinworms – against ulcers (ash) | (Hoffmann et al., 1992) |
| *Peumus boldus* Molina [Monimiaceae] | **Whole plant:** stimulant, stomachic, diuretic, antirheumatic, cholagogue, choleretic, mild hypnotic, affects nervous system, aperitive, remedy against dislocations, colds, liver ulcers, respiratory and urinary infections, digestive (tincture), diuretic (essential oil) | (Muñoz S. et al., 1981; de Mösbach, 1992; Hoffmann et al., 1992; Bussmann and Sharon, 2015) |
| *Schinus latifolia* (Gillies ex Lindl.) Engl. (syn. *Lithraea molle* Gay) [Anacardiaceae] | **Fermented beverage:** refrigerant  **Fruits:** carminative, cicatrizant, diuretic, galactagogue, laxative, tonic, against hemorrhoids (baked or fresh fruits’ decoction) | (Murillo, 1889; Muñoz S. et al., 1981; Pardo and Pizarro, 2013, 2016) |
| *Schinus molle* L. [Anacardiaceae] | **Fermented beverage:** astringent  **Fruits:** anti-emetic, aperitive, stomachic (crushed fruits as warm poultice or fruit juice with sugar) | (Cobo, 1964; Muñoz S. et al., 1981; Pardo and Pizarro, 2013, 2016) |
| *Schinus polygama* (Cav.) Cabrera (syn. *Duvaua dependens* DC.) [Anacardiaceae] | **Seeds:** against urinary infections, oedemas (infusion)  **Whole plant:** against urinary infections and oedemas (infusion) – carminative, digestive, emollient, against bronchitis, rheumatisms, skin irritations, ulcers, urinary infections (decoction) | (Murillo, 1889; Lenz, 1910; Muñoz S. et al., 1981) |
| *Ugni molinae* Turcz. (syn. *Ugni philippii* O.Berg, *Ugni poeppigii* O.Berg) [Myrtaceae] | **Fermented beverage:** aperitive, stomachic  **Whole plant:** stimulant, astringent, stomachic  **Fruits:** astringent, stimulant | (Espinoza, 1897; Muñoz S. et al., 1981; Molina, 1987; de Mösbach, 1992) |
| *Ugni selkirkii* (Hook. & Arn.) O.Berg (syn. *Ugni berteroi* (Phil.) F.Phil.) [Myrtaceae] | **Fruits:** astringent, stimulant | (de Mösbach, 1992) |

**References**

Bussmann, R. W., and Sharon, D. (2015). *Plantas medicinales de los Andes y la Amazonia. La Flora mágica y medicinal del Norte del Perú.* Peru.

Cárdenas Álvarez, R., and Villagrán, C. (2005). *Chiloé: Botánica de la cotidianidad.* Santiago, Chile: Consejo Nacional del Libro y la Lectura.

Cobo, B. (1964). *Obras del Bernabé Cobo. Tomo I.*, ed. F. Mateos. Madrid: Atlas.

de Mösbach, E. W. (1992). *Botánica indígena de Chile*. Chile: Editorial Andrés Bello.

Espinoza, E. (1897). *Plantas medicinales de Chile: Fragmento de la cuarta edición de la Jeografía Descriptiva de la República de Chile.* Santiago de Chile: Imprenta i Encuadernación Barcelona.

Hoffmann, A., Farga, C., Lastra González, J. de la, and Veghazi, E. (1992). *Plantas medicinales de uso común en Chile*. Santiago, Chile: Fundación Claudio Gay.

Lenz, R. (1910). *Diccionario etimológico de las voces chilenas derivadas de lenguas indígenas americanas*., ed. M. Ferreccio Podestá. Santiago, Chile: Universidad de Chile.

Molina, C. I. (1987). *Ensayo sobre la historia natural de Chile*. Santiago, Chile: Ediciones Maule.

Muñoz S., M., Barrera M., E., and Meza P., I. (1981). *El uso medicinal y alimenticio de plantas nativas y naturalizadas en Chile*. Santiago de Chile: Museo Nacional de Historia Nacional.

Murillo, A. (1889). *Plantes médicinales du Chili*. Paris: Exposition Universelle de Paris, Section chilienne.

Pardo, O., and Pizarro, J. L. (2013). *Chile: Plantas alimentarias prehispánicas.* Arica: Ediciones Parina.

Pardo, O., and Pizarro, J. L. (2016). *Chile. Bebidas fermentadas prehispánicas.* Arica: Ediciones Parina.

Rapoport, E. H., Ladio, A., and Sanz, E. H. (2003a). *Plantas comestibles de la Patagonia andina argentino/chilena. Parte I*. Bariloche, Argentina: Ediciones de Imaginaria.

Rapoport, E. H., Ladio, A., and Sanz, E. H. (2003b). *Plantas comestibles de la Patagonia andina argentino/chilena. Parte II*. Bariloche, Argentina: Ediciones de Imaginaria.
