# Supplementary material for: Fermented beverages in prehispanic Chile: a comprehensive review of their phytochemistry, traditional medicinal uses, bioactivity, and social aspects
Source: Front Pharmacol. 2024 Nov 21;15:1505873. doi: 10.3389/fphar.2024.1505873 (PMC11617176; doi:10.3389/fphar.2024.1505873)
Supplement: Supplementary file 3 [file Table3.DOCX]

**Table S3.** Evidence-based bioactivity of the raw materials used in the elaboration of prehispanic fermented beverages in Chile. Only the bioactivity of the part used in fermentation (**Table 1**) has been considered.

| **Species - Family** | **Bioactivity** | **References** |
| --- | --- | --- |
| *Araucaria araucana* (Molina) K.Koch (syn. *Araucaria imbricata* Pav.) [Araucariaceae] | Antioxidant capacity *in vitro* and *in vivo* (extracts) | (Schmeda-Hirschmann et al., 2021) |
|  | Goitrogenic activity *in vivo* (seed flour) | (Tellez et al., 1969) |
|  | Inhibition *in vitro* of *α*-amylase and *α*-glucosidase (extracts) | (Schmeda-Hirschmann et al., 2021) |
|  | Inhibition of lipoperoxidation *in vitro* (extracts) | (Gallia et al., 2020) |
| *Aristotelia chilensis* (Molina) Stuntz [Elaeocarpaceae] | Antibacterial activity against *Achromobacter denitrificans*, *Aeromonas hydrophila*, *Alcaligenes faecalis*, *Enterobacter amnigenus, E. gergoviae*, *Escherichia coli,* *Listeria monocytogenes, L. innocua*, *Serratia marcescens*, and *Shewanella putrefaciens* (extracts) | (Genskowsky et al., 2016) |
|  | Antidepressive effect *in vivo* in post-stroke mice (extracts) | (Di Lorenzo et al., 2019) |
|  | Anti-diabetic effects on H4IIE rat liver cells and L6 myotubes (extracts) | (Rojo et al., 2012) |
|  | Anti-inflammatory effect on human visceral preadipocytes, THP-1 & RAW 264.7 macrophages (alone or in co-culture with 3T3-L1 adipocytes), against TPA-induced ear oedema & against DNCB-atopic dermatitis in mice (extracts) | (Céspedes et al., 2010a, 2010b, 2017; Schreckinger et al., 2010; Reyes-Farias et al., 2014; Céspedes-Acuña et al., 2018; Ovalle-Marin et al., 2020; Quispe-Fuentes et al., 2020) |
|  | Antinociceptive effect in mice subjected to formalin test (extracts) | (Agulló et al., 2021a, 2021b) |
|  | Antioxidant capacity *in vitro,* in 661W, HUVEC cells, THP-1 macrophages, human platelets & human visceral preadipocytes, *in vivo* in mice & rats and in healthy & overweight adults & adult smokers (fermented and not fermented extracts, isolated compounds) | (Miranda-Rottmann et al., 2002; Céspedes et al., 2009, 2010a, 2010b, 2017; Ruiz et al., 2010; Schreckinger et al., 2010; Rubilar et al., 2011; Gironés-Vilaplana et al., 2012, 2014; Tanaka et al., 2013; Fredes et al., 2014; Genskowsky et al., 2016; Lucas-Gonzalez et al., 2016; Reyes-Farias et al., 2016; Rodríguez et al., 2016; Li et al., 2017; Nowak et al., 2018; Quispe-Fuentes et al., 2018, 2019; Céspedes-Acuña et al., 2018; Bribiesca-Cruz et al., 2021; Roldán et al., 2021; Concha-Meyer et al., 2021; del Campo et al., 2021; Bastías-Montes et al., 2022; Gomez Mattson et al., 2022; Peçanha et al., 2022; Pinto et al., 2022) |
|  | Antiplatelet effect on human platelet-rich plasma treated with several agonists (extracts) | (Rodríguez et al., 2021) |
|  | Antiproliferative effect on HT-29 (colon), Caco-2 (colon) and Ishikawa (endometrium) cancer cells (extracts) | (Céspedes-Acuña et al., 2018; Mena et al., 2021) |
|  | Anti-tyrosinase and anti-cholinesterase activity *in vitro* (extracts) | (Céspedes et al., 2017) |
|  | Cardioprotective activity against reperfusion dysrhythmias in rats (extracts) | (Céspedes et al., 2008) |
|  | Cognitive deficit prevention in rats after chronic exposure to ozone (extracts) | (Bribiesca-Cruz et al., 2021) |
|  | Hypoglycemic effect on obese hyperglycemic mice and prediabetic individuals (extracts) | (Alvarado et al., 2016; Sandoval et al., 2019) |
|  | Hypoinsulinemic effect on prediabetic individuals (extracts) | (Alvarado et al., 2016) |
|  | Inhibition of α-amylase, α-glucosidase, aldose reductase and pancreatic lipase *in vitro* (extracts) | (Schreckinger et al., 2012; Gironés-Vilaplana et al., 2014; Pineda et al., 2022) |
|  | Inhibition of DBNB-induced atopic dermatitis indications in mice (extracts) | (Moon and Kim, 2020) |
|  | Inhibition of COX-2 and iNOS protein expression in RAW 264.7 macrophages **(fermented and not fermented extracts)** | (Wang et al., 2012) |
|  | Inhibition of lipid accumulation in 3T3-L1 adipocytes (extracts) | (Schreckinger et al., 2010) |
|  | Inhibition of high-fat diet-induced hepatic lipogenesis in mice (extracts) | (Sandoval et al., 2019, 2021; del Campo et al., 2021) |
|  | Inhibition of lipoperoxidation *in vitro* (extracts) | (Céspedes et al., 2008, 2009, 2010a, 2010b, 2017; Céspedes-Acuña et al., 2018; Bribiesca-Cruz et al., 2021) |
|  | Inhibition of human LDL oxidation *in vitro* (extracts) | (Miranda-Rottmann et al., 2002) |
|  | Inhibition of light-induced 661W photoreceptor cell death (extracts, isolated compounds) | (Tanaka et al., 2013) |
|  | Mitigation of insulin resistance in high diet-induced obese mice (extracts) | (Sandoval et al., 2019; del Campo et al., 2021) |
|  | Neuroprotective effects mice neurons model of amyloid-β-induced Alzheimer’s disease (extracts) | (Fuentealba et al., 2012) |
|  | Normalization of IL-6 and H_2_O_2_ concentrations in exhaled breath condensate from individuals with mild smoking habits (extracts) | (Vergara et al., 2015) |
|  | Prevention of olanzapine-induced lipid accumulation in HepG2 (liver) cells (isolated compounds) | (del Campo et al., 2021) |
|  | Photoprotective effect on HaCaT (keratinocyte) cells and in mice | (Chen et al., 2020) |
|  | Quinone reductase-inducing effect in murine Hepa1c1c7 cells (extracts, isolated compounds) | (Li et al., 2017) |
|  | Tear secretion restoration in rat blink-suppressed dry eye model (extracts, isolated compounds) | (Nakamura et al., 2014) |
|  | Therapeutic effects against DSS-induced ulcerative colitis in mice (extracts) | (Zhou et al., 2019) |
| *Berberis darwinii* Hook. [Berberidaceae] | Antioxidant capacity *in vitro* (extracts) | (Chamorro et al., 2019) |
|  | Inhibition of α-glucosidase *in vitro* (extracts) |  |
| *Berberis microphylla* G. Forst. (syn*. Berberis buxifolia* Lam*.*, *Berberis parodii* Job) [Berberidaceae] | Anti-inflammatory effect on human visceral preadipocytes, THP-1 & RAW 264.7 macrophages (alone or in co-culture with 3T3-L1 adipocytes) and on diet-induced insulin-resistant C57BL/6 mice (extracts) | (Ovalle-Marin et al., 2020) |
|  | Antioxidant capacity *in* vitro, in HUVEC cells, endothelial cells from rat mesentery, THP-1 macrophages & human visceral preadipocytes, and *in vivo* in AAPH-challenged zebrafish embryos (extracts) | (Ruiz et al., 2010, 2013; Brito et al., 2014; Ramirez et al., 2015; Arena et al., 2017; López et al., 2018; Calfío and Huidobro-Toro, 2019; Chamorro et al., 2019; Boeri et al., 2020; Ovalle-Marin et al., 2020; Romero-Román et al., 2021b, 2021a; Pinto-Morales et al., 2022) |
|  | Attenuation of high-fat diet effects on the expression of thermogenic and browning markers in inguinal white adipose tissue in obese mice (extracts\|) | (Duarte et al., 2021) |
|  | Decrease high-fat diet-induced body weight gain in mice (extracts) | (Olivares-Caro et al., 2020) |
|  | Decrease of *in vitro* viability and migration capacity of human gastric carcinoma (AGC) and gallbladder carcinoma (G415) cell lines (extracts) | (Calderón-Reyes et al., 2020) |
|  | Improvement of brown adipose tissue mitochondrial function in high-fat diet-induced obese mice (extracts) | (Ramirez et al., 2015) |
|  | Improvement of glucose tolerance in diet-induced insulin-resistant C57BL/6 mice (extracts) | (Soto-Covasich et al., 2020) |
|  | Increase of energy expenditure in high-fat diet-induced obese mice (extracts) | (Ramirez et al., 2015) |
|  | Inhibition of acetylcholinesterase (extracts microencapsulated in maltodextrin) and α-glucosidase *in vitro* (extracts) | (Chamorro et al., 2019; Romero-Román et al., 2021b) |
|  | Inhibition of human LDL oxidation *in vitro* (extracts) | (Olivares-Caro et al., 2020) |
|  | Inhibition of lipid peroxidation in human erythrocytes (extracts) | (Olivares-Caro et al., 2020) |
|  | Prevention of amyloid beta (Aβ)-induced cytotoxicity in PC12 cells (extracts microencapsulated in maltodextrin) | (Romero-Román et al., 2021b) |
|  | Vasodilation in rat arterial mesenteric bed model (extracts) | (Calfío and Huidobro-Toro, 2019) |
| *Chenopodium pallidicaule* Aellen [Amaranthaceae] | Antianemic effect on mice (flour) | (Moscoso-Mujica et al., 2022) |
|  | Antioxidant capacity *in vitro* (extracts) | (Peñarrieta et al., 2008; Abderrahim et al., 2012; Coronado-Olano et al., 2021) |
|  | Hypoglycemic effect on mice (extracts) | (Zambrana et al., 2020) |
|  | Inhibition of α-amylase and α-glucosidase *in vitro* (extracts) | (Galvez Ranilla et al., 2009; Coronado-Olano et al., 2021) |
| *Fragaria chiloensis* (L.) Mill (syn. *Potentilla chiloensis* (L.) Mabb.) [Rosaceae] | Antioxidant capacity *in vitro* (extracts) | (Cheel et al., 2007; Wang and Lewers, 2007; Simirgiotis et al., 2009; Simirgiotis and Schmeda-Hirschmann, 2010; Ávila et al., 2017; Thomas-Valdés et al., 2018, 2019; Chamorro et al., 2019; Han et al., 2019; Noriega et al., 2021) |
|  | Cytoprotective effect on gastric epithelial AGS cells stressed by H_2_O_2_, AAPH (extracts) | (Ávila et al., 2017) |
|  | Inhibition of acetylcholinesterase, α-glucosidase and pancreatic lipase *in vitro* (extracts) | (Thomas-Valdés et al., 2018, 2019; Chamorro et al., 2019; Noriega et al., 2021) |
|  | Inhibition of colonic inflammation in dextran-sulfate-sodium-treated mice (fruit powder as feed supplement) | (Han et al., 2019) |
|  | Inhibition of lipid peroxidation in human erythrocytes (extracts) | (Cheel et al., 2007) |
| *Gaultheria mucronata* (L.f.) Hook. & Arn. [Ericaceae] | Antioxidant capacity *in vitro* (extracts) | (Ruiz et al., 2013, 2015) |
| *Gaultheria poeppigii* DC. (syn. *Pernettya myrtilloides* Zucc. ex Steud.) [Ericaceae] | Antioxidant capacity *in vitro* (extracts) | (Mieres-Castro et al., 2019; Oyarzún et al., 2020) |
|  | Inhibition *in vitro* of *α*-amylase, *α*-glucosidase (extracts, isolated compounds) | (Mieres-Castro et al., 2022) |
|  | Reduction of glucose uptake in Caco-2 cells (extracts, isolated compounds) | (Mieres-Castro et al., 2022) |
|  | Reduction of mRNA expression of glucose transporters and sucrose-isomaltase in Caco-2 cells (extracts, isolated compounds) | (Mieres-Castro et al., 2022) |
| *Geoffroea decorticans* (Gillies ex Hook. & Arn.) Burkat [Fabaceae] | Anti-inflammatory effect against carrageenan induced edema and cotton pellet-induced granuloma formation in rats (extracts) | (Reynoso et al., 2013) |
|  | Antinociceptive effect in rats (extracts) | (Reynoso et al., 2013) |
|  | Antioxidant capacity *in vitro* (extracts) | (Costamagna et al., 2013, 2016, 2017; Reynoso et al., 2013; Jiménez-Aspee et al., 2017; Somaini et al., 2021) |
|  | Antitussive activity against ammonia induced cough in rats (extracts) | (Reynoso et al., 2013) |
|  | Expectorant effect in rats (extracts) | (Reynoso et al., 2013) |
|  | Inhibition *in vitro* of *α*-amylase, *α*-glucosidase, lipase, HMG-CoA reductase, cyclooxygenase, lipoxygenase, phospholipase A2 (extracts) | (Costamagna et al., 2013, 2016, 2017; Jiménez-Aspee et al., 2017) |
|  | Inhibition of the wnt/β-catenin signaling pathway in *Xenopus* embryos (extracts) | (Somaini et al., 2021) |
| *Greigia sphacelata* (Ruiz & Pav.) Regel [Bromeliaceae] | Antioxidant capacity *in vitro* (extracts) | (Aravena-Sanhueza et al., 2020; Barrientos et al., 2020) |
|  | Inhibition *in vitro* of acetylcholinesterase and butyrylcholinesterase (extracts) | (Barrientos et al., 2020) |
| *Luma apiculata* (DC.) Burret (syn. *Myrceugenella apiculata* (DC.) Kausel) [Myrtaceae] | Antibacterial activity against *Staphylococcus aureus* *in vitro* (extracts) | (Viktorová et al., 2020; Velásquez et al., 2022) |
|  | Antioxidant capacity *in vitro* (extracts) | (Simirgiotis et al., 2013; Brito et al., 2014; Ramirez et al., 2015; Fuentes et al., 2016; Velásquez et al., 2022) |
|  | Inhibition of lipid peroxidation in human erythrocytes (extracts) | (Ramirez et al., 2015) |
|  | Vascular protective effect in rat thoracic aorta rings under high glucose conditions (extracts) | (Fuentes et al., 2016) |
| *Neltuma alba* (Griseb.) C.E.Hughes & G.P.Lewis (syn. *Prosopis alba* Griseb.) [Fabaceae] | Antioxidant capacity *in vitro* and in human blood cells previously treated with Stx and Hly toxins of *Escherichia coli* or with chloramphenicol (extracts, **beverages produced after spontaneous fermentation**) | (Schmeda-Hirschmann et al., 1999; Albrecht et al., 2010, 2011; Cardozo et al., 2010; Cattaneo et al., 2014, 2016; Pérez et al., 2014; Rodriguez et al., 2019; Rodríguez et al., 2020; Sciammaro et al., 2021; Correa Uriburu et al., 2022) |
|  | Cytoprotective effect in Vero cells treated with Stx toxin (extracts) | (Pellarín et al., 2013) |
|  | Anti-inflammatory activity by inhibition *in vitro* of cyclooxygenase, lipoxygenase, phospholipase A2 (extracts) | (Cattaneo et al., 2014, 2016; Pérez et al., 2014) |
|  | Inhibition *in vitro* of lipid peroxidation (extracts; **beverages produced after spontaneous fermentation**) | (Pérez et al., 2014; Rodriguez et al., 2019) |
|  | Vasoprotective effect on rabbits suffering from high fat diet-induced metabolic syndrome (seed flour feed supplement) | (Cattaneo et al., 2019) |
| *Neltuma chilensis* (Molina) C.E.Hughes & G.P.Lewis (syn. *Prosopis chilensis* (Molina) Stuntz) [Fabaceae] | Antioxidant capacity *in vitro* (extracts) | (Schmeda-Hirschmann et al., 2015) |
| *Otholobium glandulosum* (L.) J.W.Grimes (syn. *Psoralea glandulosa* L.) [Fabaceae] | Antioxidant capacity *in vitro* (extract, isolated compounds) | (Madrid et al., 2013) |
| *Peumus boldus* Molina [Monimiaceae] | Anti-inflammatory effect *in vitro* (extracts) | (Otero et al., 2022) |
|  | Mild cytotoxic effect on murine macrophage RAW 264.7 cells (extracts, isolated compound) |  |
| *Prumnopitys andina* (Poepp. ex Endl.) de Laub. (syn. *Podocarpus andinus* Poepp. ex Endl.) [Podocarpacea] | Antioxidant capacity *in vitro* and in AGS cells (extract & isolated compounds) | (Jiménez-Aspee et al., 2019) |
|  | Inhibition of α-glucosidase *in vitro* (extract, isolated compounds) |  |
|  | Inhibition of lipoperoxidation *in vitro* (extracts) |  |
| *Ribes magellanicum* Poir. [Grossulariaceae] | Antioxidant capacity *in vitro* & in human gastric adenocarcinoma AGS cells (extracts & extracts previously submitted to *in vitro* colonic fermentation) | (Ruiz et al., 2013, 2015; Jiménez-Aspee et al., 2016b; Ávila et al., 2017; Burgos-Edwards et al., 2017; Theoduloz et al., 2018; Burgos-Edwards et al., 2019, 2020, 2024) |
|  | Cytoprotective effect on gastric epithelial AGS cells stressed by H_2_O_2_, AAPH or H_2_O_2_/CuSO_4_ (extracts) | (Ávila et al., 2017) |
|  | Downregulation of mRNA expression of cyclooxygenase 2 (COX-2) and inducible nitric oxide synthase (iNOS) in Caco-2 cells stimulated with IL-1β (extracts previously submitted to *in vitro* gastrointestinal digestion) | (Burgos-Edwards et al., 2019) |
|  | Increase of *Clostridium* cluster XIVa and *Akkermansia muciniphila* bacteria after *in vitro* gastrointestinal digestion and colonic fermentation (extracts) | (Burgos-Edwards et al., 2020) |
|  | Inhibition *in vitro* of *α*-amylase, *α*-glucosidase, lipase (extracts & extracts previously submitted to *in vitro* colonic fermentation) | (Burgos-Edwards et al., 2017) |
|  | Reduction of IL-6 and TNF-α in Caco-2 cells stimulated with IL-1β (extracts previously submitted to *in vitro* gastrointestinal digestion) | (Burgos-Edwards et al., 2019) |
| *Rubus geoides* Sm. [Rosaceae] | Antioxidant capacity *in vitro* (extracts) | (Ruiz et al., 2013, 2015; Jiménez-Aspee et al., 2016a) |
|  | Cytoprotective effect on gastric epithelial AGS cells stressed by H_2_O_2_, AAPH, H_2_O_2_/CuSO_4_ or methylglyoxal (MGO) (extracts) | (Ávila et al., 2017) |
|  | Increase of intracellular glutathione (GSH) in AGS cells (extracts) | (Jiménez-Aspee et al., 2016a) |
| *Schinus molle* L. [Anacardiaceae] | Antibacterial activity against *Bacillus cereus*, *Bacillus subtilis*, *Enterococcus faecalis*, *Escherichia coli*, *Haemophilus influenzae* (resistant strain to penicillin, methicillin), *Klebsiella oxytoca*, *Klebsiella pneumoniae*, *Listeria monocytogenes*, *Micrococcus luteus*, *Mycobacterium tuberculosis*, *Proteus mirabilis*, *Pseudomonas aeruginosa*, *Pseudomonas morgani*, *Salmonella anatum*, *Salmonella enteritidis*, *Salmonella typhimurium*, *Staphylococcus aureus* (susceptible and multidrug-resistant strains), *Staphylococcus* epidermidis, *Streptococcus pneumoniae* (essential oil, extracts) | (Hayouni et al., 2008; Hosni et al., 2011; Pérez-López et al., 2011; Rocha et al., 2012; Martins et al., 2014; Al-Andal et al., 2019) |
|  | Antifungal activity against *Aspergillus japonicus*, *Aspergillus niger*, *Aspergillus* *oryzae*, *Candida albicans*, *Fusarium oxysporum*, *Saccharomyces cerevisiae* (essential oil) | (Martins et al., 2014) |
|  | Anti-inflammatory effect against LPS-challenged RAW264.7 macrophages & UVB-challenged HaCaT cells; *in vivo* against acute paw (phospholipase A_2_- or carrageenan-induced) and chronic ear (TPA-induced) inflammation in mice and rats (extracts, isolated compounds) | (Yueqin et al., 2003; Feriani et al., 2021; Kim et al., 2021) |
|  | Antinociceptive effect on rats submitted to acetic acid writhing, hot plate and formalin tests (extracts) | (Feriani et al., 2021) |
|  | Antioxidant capacity *in vitro* and in LPS-challenged RAW264.7 macrophages (essential oil, isolated compounds) | (Ono et al., 2008; Bendaoud et al., 2010; Galvez Ranilla et al., 2010; Hosni et al., 2011; Martins et al., 2014; Eryigit et al., 2017; Tlili et al., 2018; Volpini-Klein et al., 2020; Feriani et al., 2021; Kim et al., 2021; Belhoussaine et al., 2022) |
|  | Antiproliferative effect on human cancer cell lines MCF-7 (breast), T47D (breast), Caco-2 (colon), HCT-116 (colon) and HepG2 (liver) (essential oil) | (Ferrero et al., 2006; Feriani et al., 2021, 2022; Osman et al., 2021) |
|  | Cardioprotective effect against isoproterenol‑induced infarction in rats (extracts) | (Feriani et al., 2021, 2022) |
|  | Inhibition *in vitro* of acetylcholinesterase, ACE, *α*-amylase, *α*-glucosidase (essential oil, extracts) | (Galvez Ranilla et al., 2010; Aboalhaija et al., 2019; Feriani et al., 2021) |
|  | Inhibition of H_2_O_2_-induced oxidative hemolysis in human erythrocytes (extracts) | (Feriani et al., 2021) |
|  | Repellent and ovicidal activity against the Chagas’ disease vector, *Triatoma infestans* (extracts) | (Ferrero et al., 2006) |
| *Ugni molinae* Turcz. (syn. *Ugni philippii* O.Berg, *Ugni poeppigii* O.Berg) [Myrtaceae] | Antibacterial activity against *Escherichia coli*, *Listeria monocytogenes*, *Salmonella typhi* and *Staphylococcus aureus* (extracts) | (Junqueira-Gonçalves et al., 2015; López de Dicastillo et al., 2017) |
|  | Antioxidant capacity *in vitro* and in HUVEC-C cells, human erythrocytes & in boar sperm cells (extracts) | (Ruiz et al., 2010; Rubilar et al., 2011; Brito et al., 2014; Rodríguez et al., 2014; Suwalsky and Avello, 2014; Junqueira-Gonçalves et al., 2015; Ramirez et al., 2015; Jofré et al., 2016, 2019; López de Dicastillo et al., 2017; López et al., 2017b, 2017a, 2019; Gómez-Pérez et al., 2022) |
|  | Inhibition *in vitro* of α-amylase, α-glucosidase and tyrosinase | (Rubilar et al., 2011; Junqueira-Gonçalves et al., 2015) |
|  | Inhibition of lipid peroxidation *in vitro* and in HUVEC-C cells & human erythrocytes (extracts) | (Jofré et al., 2016) |
|  | Prebiotic effect on human gut beneficial bacteria | (Shene et al., 2009) |
|  | Reduction of abnormal protein aggregation in Huntington’s disease cellular model (extracts) | (Pérez-Arancibia et al., 2021) |
|  | Vasodilator effect in rat aortic rings (extracts) | (Jofré et al., 2016) |

**References**

Abderrahim, F., Huanatico, E., Repo-Carrasco-Valencia, R., Arribas, S. M., Gonzalez, M. C., and Condezo-Hoyos, L. (2012). Effect of germination on total phenolic compounds, total antioxidant capacity, Maillard reaction products and oxidative stress markers in canihua (Chenopodium pallidicaule). *J. Cereal Sci.* 56, 410–417. doi: https://doi.org/10.1016/j.jcs.2012.04.013

Aboalhaija, N. H., Amro, R., Abaza, I. F., Khalil, Al-Aboudi, A., Abu-Zarga, M., et al. (2019). Schinus molle L. Collected from Jordan and Turkey: Essential Oil Composition and Anticholinesterase Activity. *J. Essent. Oil Bear. Plants* 22, 704–716. doi: 10.1080/0972060X.2019.1639552

Agulló, V., González-Trujano, M. E., Hernandez-Leon, A., Estrada-Camarena, E., Pellicer, F., and García-Viguera, C. (2021a). Antinociceptive effects of maqui-berry (Aristotelia chilensis (Mol.) Stuntz). *Int. J. Food Sci. Nutr.* 72, 947–955. doi: 10.1080/09637486.2021.1895727

Agulló, V., González-Trujano, M. E., Hernandez-Leon, A., Estrada-Camarena, E., Pellicer, F., and García-Viguera, C. (2021b). Synergistic Interaction in the Analgesic-Like Effects of Maqui Berry and Citrus Is Antagonized by Sweeteners. *Nutrients* 13. doi: 10.3390/nu13072466

Al-Andal, A., Moustafa, M., and Alrumman, S. (2019). Variations in Chemicals and Antimicrobial Properties of Schinus molle Fruits Grown in Abha Area, Saudi Arabia. *Arab. J. Sci. Eng.* 44, 87–101. doi: 10.1007/s13369-018-3660-x

Albrecht, C., Pellarin, G., Rojas, M. J., Albesa, I., and Eraso, A. J. (2010). Beneficial effect of Berberis buxifolia Lam, Zizyphus mistol Griseb and Prosopis alba extracts on oxidative stress induced by chloramphenicol. *Med. (B Aires)* 70, 65–70.

Albrecht, C., Pellarin, M. G., Baronetti, J., Rojas, M. J., Albesa, I., and Eraso, A. J. (2011). Chemiluminescence determination of antioxidant property of Zizyphus mistol and Prosopis alba during oxidative stress generated in blood by Hemolytic Uremic Syndrome-producing Escherichia coli. *Luminescence* 26, 424–428. doi: https://doi.org/10.1002/bio.1247

Alvarado, J. L., Leschot, A., Olivera-Nappa, Á., Salgado, A.-M., Rioseco, H., Lyon, C., et al. (2016). Delphinidin-Rich Maqui Berry Extract (Delphinol®) Lowers Fasting and Postprandial Glycemia and Insulinemia in Prediabetic Individuals during Oral Glucose Tolerance Tests. *Biomed Res. Int.* 2016, 9070537. doi: 10.1155/2016/9070537

Aravena-Sanhueza, F., Pérez-Rivera, M., Castillo-Felices, R., Mundaca-Uribe, R., Aranda Bustos, M., and Peña Farfal, C. (2020). Determination of antioxidant capacity (ORAC) of Greigia sphacelata and correlation with voltammetric methods. *J. Chil. Chem. Soc.* 65, 4925–4928.

Arena, M. E., Postemsky, P. D., and Curvetto, N. R. (2017). Changes in the phenolic compounds and antioxidant capacity of Berberis microphylla G. Forst. berries in relation to light intensity and fertilization. *Sci. Hortic. (Amsterdam).* 218, 63–71. doi: https://doi.org/10.1016/j.scienta.2017.02.004

Ávila, F., Theoduloz, C., López-Alarcón, C., Dorta, E., and Schmeda-Hirschmann, G. (2017). Cytoprotective Mechanisms Mediated by Polyphenols from Chilean Native Berries against Free Radical-Induced Damage on AGS Cells. *Oxid. Med. Cell. Longev.* 2017, 9808520. doi: 10.1155/2017/9808520

Barrientos, R. E., Ahmed, S., Cortés, C., Fernández-Galleguillos, C., Romero-Parra, J., Simirgiotis, M. J., et al. (2020). Chemical Fingerprinting and Biological Evaluation of the Endemic Chilean Fruit Greigia sphacelata (Ruiz and Pav.) Regel (Bromeliaceae) by UHPLC-PDA-Orbitrap-Mass Spectrometry. *Molecules* 25. doi: 10.3390/molecules25163750

Bastías-Montes, J. M., Vidal-San-Martín, C., Tamarit-Pino, Y., Muñoz-Fariña, O., García-Figueroa, O., Quevedo-León, R., et al. (2022). Cryoconcentration by Centrifugation&ndash;Filtration: A Simultaneous, Efficient and Innovative Method to Increase Thermosensitive Bioactive Compounds of Aqueous Maqui (Aristotelia chilensis (Mol.) Stuntz) Extract. *Processes* 10. doi: 10.3390/pr10010025

Belhoussaine, O., El Kourchi, C., Harhar, H., Bouyahya, A., El Yadini, A., Fozia, F., et al. (2022). Chemical Composition, Antioxidant, Insecticidal Activity, and Comparative Analysis of Essential Oils of Leaves and Fruits of *Schinus molle* and *Schinus terebinthifolius*. *Evidence-Based Complement. Altern. Med.* 2022, 4288890. doi: 10.1155/2022/4288890

Bendaoud, H., Romdhane, M., Souchard, J. P., Cazaux, S., and Bouajila, J. (2010). Chemical Composition and Anticancer and Antioxidant Activities of Schinus Molle L. and Schinus Terebinthifolius Raddi Berries Essential Oils. *J. Food Sci.* 75, C466–C472. doi: https://doi.org/10.1111/j.1750-3841.2010.01711.x

Boeri, P., Piñuel, L., Dalzotto, D., Monasterio, R., Fontana, A., Sharry, S., et al. (2020). Argentine Patagonia barberry chemical composition and evaluation of its antioxidant capacity. *J. Food Biochem.* 44, e13254. doi: https://doi.org/10.1111/jfbc.13254

Bribiesca-Cruz, I., Moreno, D. A., García-Viguera, C., Gallardo, J. M., Segura-Uribe, J. J., Pinto-Almazán, R., et al. (2021). Maqui berry (Aristotelia chilensis) extract improves memory and decreases oxidative stress in male rat brain exposed to ozone. *Nutr. Neurosci.* 24, 477–489. doi: 10.1080/1028415X.2019.1645438

Brito, A., Areche, C., Sepúlveda, B., Kennelly, E. J., and Simirgiotis, M. J. (2014). Anthocyanin Characterization, Total Phenolic Quantification and Antioxidant Features of Some Chilean Edible Berry Extracts. *Molecules* 19, 10936–10955. doi: 10.3390/molecules190810936

Burgos-Edwards, A., Fernández-Romero, A., Carmona, M., Thuissard-Vasallo, I., Schmeda-Hirschmann, G., and Larrosa, M. (2020). Effects of gastrointestinal digested polyphenolic enriched extracts of Chilean currants (Ribes magellanicum and Ribes punctatum) on in vitro fecal microbiota. *Food Res. Int.* 129, 108848. doi: https://doi.org/10.1016/j.foodres.2019.108848

Burgos-Edwards, A., Jiménez-Aspee, F., Thomas-Valdés, S., Schmeda-Hirschmann, G., and Theoduloz, C. (2017). Qualitative and quantitative changes in polyphenol composition and bioactivity of Ribes magellanicum and R. punctatum after in vitro gastrointestinal digestion. *Food Chem.* 237, 1073–1082. doi: https://doi.org/10.1016/j.foodchem.2017.06.060

Burgos-Edwards, A., Martín-Pérez, L., Jiménez-Aspee, F., Theoduloz, C., Schmeda-Hirschmann, G., and Larrosa, M. (2019). Anti-inflammatory effect of polyphenols from Chilean currants (Ribes magellanicum and R. punctatum) after in vitro gastrointestinal digestion on Caco-2 cells: Anti-inflammatory activity of in vitro digested Chilean currants. *J. Funct. Foods* 59, 329–336. doi: https://doi.org/10.1016/j.jff.2019.06.007

Burgos-Edwards, A., Theoduloz, C., Miño, S., Ghosh, D., Shulaev, V., Ramírez, C., et al. (2024). Phenolic composition and bioactivity of Ribes magellanicum fruits from southern Patagonia. *Heliyon* 10, e25542. doi: 10.1016/j.heliyon.2024.e25542

Calderón-Reyes, C., Pezoa, R. S., Leal, P., Ribera-Fonseca, A., Cáceres, C., Riquelme, I., et al. (2020). Anthocyanin-Rich Extracts of Calafate (Berberis microphylla G. Forst.) Fruits Decrease In Vitro Viability and Migration of Human Gastric and Gallbladder Cancer Cell Lines. *J. Soil Sci. Plant Nutr.* 20, 1891–1903. doi: 10.1007/s42729-020-00260-8

Calfío, C., and Huidobro-Toro, J. P. (2019). Potent Vasodilator and Cellular Antioxidant Activity of Endemic Patagonian Calafate Berries (Berberis microphylla) with Nutraceutical Potential. *Molecules* 24. doi: 10.3390/molecules24152700

Cardozo, M. L., Ordoñez, R. M., Zampini, I. C., Cuello, A. S., Dibenedetto, G., and Isla, M. I. (2010). Evaluation of antioxidant capacity, genotoxicity and polyphenol content of non conventional foods: Prosopis flour. *Food Res. Int.* 43, 1505–1510. doi: https://doi.org/10.1016/j.foodres.2010.04.004

Cattaneo, F., Costamagna, M. S., Zampini, I. C., Sayago, J., Alberto, M. R., Chamorro, V., et al. (2016). Flour from Prosopis alba cotyledons: A natural source of nutrient and bioactive phytochemicals. *Food Chem.* 208, 89–96. doi: https://doi.org/10.1016/j.foodchem.2016.03.115

Cattaneo, F., Roco, J., Alarcón, G., Isla, M. I., and Jeréz, S. (2019). <em>Prosopis alba</em> seed flour improves vascular function in a rabbit model of high fat diet-induced metabolic syndrome. *Heliyon* 5. doi: 10.1016/j.heliyon.2019.e01967

Cattaneo, F., Sayago, J. E., Alberto, M. R., Zampini, I. C., Ordoñez, R. M., Chamorro, V., et al. (2014). Anti-inflammatory and antioxidant activities, functional properties and mutagenicity studies of protein and protein hydrolysate obtained from Prosopis alba seed flour. *Food Chem.* 161, 391–399. doi: https://doi.org/10.1016/j.foodchem.2014.04.003

Céspedes-Acuña, C. L., Xiao, J., Wei, Z.-J., Chen, L., Bastias, J. M., Avila, J. G., et al. (2018). Antioxidant and anti-inflammatory effects of extracts from Maqui berry Aristotelia chilensis in human colon cancer cells. *J. Berry Res.* 8, 275–296. doi: 10.3233/JBR-180356

Céspedes, C. L., Alarcon, J., Avila, J. G., and Nieto, A. (2010a). Anti-inflammatory Activity of Aristotelia chilensis Mol. (Stuntz) (Elaeocarpaceae). *Boletín Latinoam. y del Caribe Plantas Med. y Aromáticas* 9, 127–135.

Céspedes, C. L., Alarcon, J., Valdez-Morales, M., and Paredes-López, O. (2009). Antioxidant Activity of an Unusual 3-Hydroxyindole Derivative Isolated from Fruits of Aristotelia chilensis (Molina) Stuntz. 64, 759–762. doi: doi:10.1515/znc-2009-9-1024

Céspedes, C. L., El-Hafidi, M., Pavon, N., and Alarcon, J. (2008). Antioxidant and cardioprotective activities of phenolic extracts from fruits of Chilean blackberry Aristotelia chilensis (Elaeocarpaceae), Maqui. *Food Chem.* 107, 820–829. doi: https://doi.org/10.1016/j.foodchem.2007.08.092

Céspedes, C. L., Pavon, N., Dominguez, M., Alarcon, J., Balbontin, C., Kubo, I., et al. (2017). The chilean superfruit black-berry Aristotelia chilensis (Elaeocarpaceae), Maqui as mediator in inflammation-associated disorders. *Food Chem. Toxicol.* 108, 438–450. doi: https://doi.org/10.1016/j.fct.2016.12.036

Céspedes, C. L., Valdez-Morales, M., Avila, J. G., El-Hafidi, M., Alarcón, J., and Paredes-López, O. (2010b). Phytochemical profile and the antioxidant activity of Chilean wild black-berry fruits, Aristotelia chilensis (Mol) Stuntz (Elaeocarpaceae). *Food Chem.* 119, 886–895. doi: https://doi.org/10.1016/j.foodchem.2009.07.045

Chamorro, M. F., Reiner, G., Theoduloz, C., Ladio, A., Schmeda-Hirschmann, G., Gómez-Alonso, S., et al. (2019). Polyphenol Composition and (Bio)Activity of Berberis Species and Wild Strawberry from the Argentinean Patagonia. *Molecules* 24. doi: 10.3390/molecules24183331

Cheel, J., Theoduloz, C., Rodríguez, J. A., Caligari, P. D. S., and Schmeda-Hirschmann, G. (2007). Free radical scavenging activity and phenolic content in achenes and thalamus from Fragaria chiloensis ssp. chiloensis, F. vesca and F. x ananassa cv. Chandler. *Food Chem.* 102, 36–44. doi: https://doi.org/10.1016/j.foodchem.2006.04.036

Chen, L., Zhou, G., Meng, X.-S., Fu, H.-Y., Mo, Q.-G., and Wang, Y.-W. (2020). Photoprotection of maqui berry against ultraviolet B-induced photodamage in vitro and in vivo. *Food Funct.* 11, 2749–2762. doi: 10.1039/C9FO01902B

Concha-Meyer, A. A., Sepúlveda, G., Pérez-Díaz, R., and Torres, C. A. (2021). Effect of preservation processing on quality attributes and phenolic profile of maqui (Aristotelia chilensis mol. Stuntz) fruit. *LWT* 149, 111920. doi: https://doi.org/10.1016/j.lwt.2021.111920

Coronado-Olano, J., Repo-Carrasco-Valencia, R., Reategui, O., Toscano, E., Valdez, E., Zimic, M., et al. (2021). Inhibitory activity against α-amylase and α-glucosidase by phenolic compounds of quinoa (Chenopodium quinoa Willd.) and cañihua (Chenopodium pallidicaule Aellen) from the Andean region of Peru. *Pharmacogn. J.* 13.

Correa Uriburu, F. M., Cattaneo, F., Maldonado, L. M., Zampini, I. C., Alberto, M. R., and Isla, M. I. (2022). Prosopis alba Seed as a Functional Food Waste for Food Formulation Enrichment. *Foods* 11. doi: 10.3390/foods11182857

Costamagna, M. S., Gómez-Mascaraque, L. G., Zampini, I. C., Alberto, M. R., Pérez, J., López-Rubio, A., et al. (2017). Microencapsulated chañar phenolics: A potential ingredient for functional foods development. *J. Funct. Foods* 37, 523–530. doi: https://doi.org/10.1016/j.jff.2017.08.018

Costamagna, M. S., Ordoñez, R. M., Zampini, I. C., Sayago, J. E., and Isla, M. I. (2013). Nutritional and antioxidant properties of Geoffroea decorticans, an Argentinean fruit, and derived products (flour, arrope, decoction and hydroalcoholic beverage). *Food Res. Int.* 54, 160–168. doi: https://doi.org/10.1016/j.foodres.2013.05.038

Costamagna, M. S., Zampini, I. C., Alberto, M. R., Cuello, S., Torres, S., Pérez, J., et al. (2016). Polyphenols rich fraction from Geoffroea decorticans fruits flour affects key enzymes involved in metabolic syndrome, oxidative stress and inflammatory process. *Food Chem.* 190, 392–402. doi: https://doi.org/10.1016/j.foodchem.2015.05.068

del Campo, A., Salamanca, C., Fajardo, A., Díaz-Castro, F., Bustos, C., Calfío, C., et al. (2021). Anthocyanins from Aristotelia chilensis Prevent Olanzapine-Induced Hepatic-Lipid Accumulation but Not Insulin Resistance in Skeletal Muscle Cells. *Molecules* 26. doi: 10.3390/molecules26206149

Di Lorenzo, A., Sobolev, A. P., Nabavi, S. F., Sureda, A., Moghaddam, A. H., Khanjani, S., et al. (2019). Antidepressive effects of a chemically characterized maqui berry extract (Aristotelia chilensis (Molina) Stuntz) in a mouse model of post-stroke depression. *Food Chem. Toxicol.* 129, 434–443. doi: https://doi.org/10.1016/j.fct.2019.04.023

Duarte, L., Quezada, J., Ramirez, L. A., Vasquez, K., Orellana, J. F., Villanueva, V., et al. (2021). The treatment with an extract from Calafate ( Berberis microphylla) induces transcript and protein expression of molecules involved in thermogenesis and adipocyte browning in adipose tissue from obese mice. *J. Berry Res.* 11, 267–277. doi: 10.3233/JBR-200670

Eryigit, T., Yildirim, B., Ekici, K., and Çirka, M. (2017). Chemical Composition, Antimicrobial and Antioxidant Properties of Schinus molle L. Essential Oil from Turkey. *J. Essent. Oil Bear. Plants* 20, 570–577. doi: 10.1080/0972060X.2017.1304286

Feriani, A., Tir, M., Aldahmash, W., Mnafgui, K., Hichem, A., Gómez-Caravaca, A. M., et al. (2022). In vivo evaluation and molecular docking studies of Schinus molle L. fruit extract protective effect against isoproterenol-induced infarction in rats. *Environ. Sci. Pollut. Res.* 29, 80910–80925. doi: 10.1007/s11356-022-21422-4

Feriani, A., Tir, M., Mufti, A., Caravaca, A. M. G., Contreras, M. del M., Taamalli, A., et al. (2021). HPLC–ESI–QTOF–MS/MS profiling and therapeutic effects of Schinus terebinthifolius and Schinus molle fruits: investigation of their antioxidant, antidiabetic, anti-inflammatory and antinociceptive properties. *Inflammopharmacology* 29, 467–481. doi: 10.1007/s10787-021-00791-1

Ferrero, A. A., Werdin González, J. O., and Sánchez Chopa, C. (2006). Biological activity of Schinus molle on Triatoma infestans. *Fitoterapia* 77, 381–383. doi: https://doi.org/10.1016/j.fitote.2006.03.004

Fredes, C., Yousef, G. G., Robert, P., Grace, M. H., Lila, M. A., Gómez, M., et al. (2014). Anthocyanin profiling of wild maqui berries (Aristotelia chilensis [Mol.] Stuntz) from different geographical regions in Chile. *J. Sci. Food Agric.* 94, 2639–2648. doi: https://doi.org/10.1002/jsfa.6602

Fuentealba, J., Dibarrart, A., Saez-Orellana, F., Fuentes-Fuentes, M. C., Oyanedel, C. N., Guzmán, J., et al. (2012). Synaptic Silencing and Plasma Membrane Dyshomeostasis Induced by Amyloid-β Peptide are Prevented by Aristotelia chilensis Enriched Extract. *J. Alzheimer’s Dis.* 31, 879–889. doi: 10.3233/JAD-2012-120229

Fuentes, L., Valdenegro, M., Gómez, M.-G., Ayala-Raso, A., Quiroga, E., Martínez, J.-P., et al. (2016). Characterization of fruit development and potential health benefits of arrayan (Luma apiculata), a native berry of South America. *Food Chem.* 196, 1239–1247. doi: https://doi.org/10.1016/j.foodchem.2015.10.003

Gallia, M. C., Bachmeier, E., Ferrari, A., Queralt, I., Mazzeo, M. A., and Bongiovanni, G. A. (2020). Pehuén (Araucaria araucana) seed residues are a valuable source of natural antioxidants with nutraceutical, chemoprotective and metal corrosion-inhibiting properties. *Bioorg. Chem.* 104, 104175. doi: https://doi.org/10.1016/j.bioorg.2020.104175

Galvez Ranilla, L., Apostolidis, E., Genovese, M. I., Lajolo, F. M., and Shetty, K. (2009). Evaluation of Indigenous Grains from the Peruvian Andean Region for Antidiabetes and Antihypertension Potential Using In Vitro Methods. *J. Med. Food* 12, 704–713. doi: 10.1089/jmf.2008.0122

Galvez Ranilla, L., Kwon, Y.-I., Apostolidis, E., and Shetty, K. (2010). Phenolic compounds, antioxidant activity and in vitro inhibitory potential against key enzymes relevant for hyperglycemia and hypertension of commonly used medicinal plants, herbs and spices in Latin America. *Bioresour. Technol.* 101, 4676–4689. doi: https://doi.org/10.1016/j.biortech.2010.01.093

Genskowsky, E., Puente, L. A., Pérez-Álvarez, J. A., Fernández-López, J., Muñoz, L. A., and Viuda-Martos, M. (2016). Determination of polyphenolic profile, antioxidant activity and antibacterial properties of maqui [Aristotelia chilensis (Molina) Stuntz] a Chilean blackberry. *J. Sci. Food Agric.* 96, 4235–4242. doi: https://doi.org/10.1002/jsfa.7628

Gironés-Vilaplana, A., Baenas, N., Villaño, D., Speisky, H., García-Viguera, C., and Moreno, D. A. (2014). Evaluation of Latin-American fruits rich in phytochemicals with biological effects. *J. Funct. Foods* 7, 599–608. doi: https://doi.org/10.1016/j.jff.2013.12.025

Gironés-Vilaplana, A., Valentão, P., Moreno, D. A., Ferreres, F., Garcı́a-Viguera, C., and Andrade, P. B. (2012). New Beverages of Lemon Juice Enriched with the Exotic Berries Maqui, Açaı́, and Blackthorn: Bioactive Components and in Vitro Biological Properties. *J. Agric. Food Chem.* 60, 6571–6580. doi: 10.1021/jf300873k

Gómez-Pérez, L. S., Moraga, N., Ah-Hen, K. S., Rodríguez, A., and Vega-Gálvez, A. (2022). Dietary fibre in processed murta (Ugni molinae Turcz) berries: bioactive components and antioxidant capacity. *J. Food Sci. Technol.* 59, 3093–3101. doi: 10.1007/s13197-022-05416-1

Gomez Mattson, M., Sozzi, A., Corfield, R., Gagneten, M., Franceschinis, L., Schebor, C., et al. (2022). Colorant and antioxidant properties of freeze-dried extracts from wild berries: use of ultrasound-assisted extraction method and drivers of liking of colored yogurts. *J. Food Sci. Technol.* 59, 944–955. doi: 10.1007/s13197-021-05096-3

Han, Y., Song, M., Gu, M., Ren, D., Zhu, X., Cao, X., et al. (2019). Dietary Intake of Whole Strawberry Inhibited Colonic Inflammation in Dextran-Sulfate-Sodium-Treated Mice via Restoring Immune Homeostasis and Alleviating Gut Microbiota Dysbiosis. *J. Agric. Food Chem.* 67, 9168–9177. doi: 10.1021/acs.jafc.8b05581

Hayouni, E. A., Chraief, I., Abedrabba, M., Bouix, M., Leveau, J.-Y., Mohammed, H., et al. (2008). Tunisian Salvia officinalis L. and Schinus molle L. essential oils: Their chemical compositions and their preservative effects against Salmonella inoculated in minced beef meat. *Int. J. Food Microbiol.* 125, 242–251. doi: https://doi.org/10.1016/j.ijfoodmicro.2008.04.005

Hosni, K., Jemli, M., Dziri, S., M’rabet, Y., Ennigrou, A., Sghaier, A., et al. (2011). Changes in phytochemical, antimicrobial and free radical scavenging activities of the Peruvian pepper tree (Schinus molle L.) as influenced by fruit maturation. *Ind. Crops Prod.* 34, 1622–1628. doi: https://doi.org/10.1016/j.indcrop.2011.06.004

Jiménez-Aspee, F., Theoduloz, C., Ávila, F., Thomas-Valdés, S., Mardones, C., von Baer, D., et al. (2016a). The Chilean wild raspberry (Rubus geoides Sm.) increases intracellular GSH content and protects against H2O2 and methylglyoxal-induced damage in AGS cells. *Food Chem.* 194, 908–919. doi: https://doi.org/10.1016/j.foodchem.2015.08.117

Jiménez-Aspee, F., Theoduloz, C., Pormetter, L., Mettke, J., Ávila, F., and Schmeda-Hirschmann, G. (2019). Andean Prumnopitys Andina (Podocarpacae) Fruit Extracts: Characterization of Secondary Metabolites and Potential Cytoprotective Effect. *Molecules* 24. doi: 10.3390/molecules24224028

Jiménez-Aspee, F., Theoduloz, C., Soriano, M. D. P. C., Ugalde-Arbizu, M., Alberto, M. R., Zampini, I. C., et al. (2017). The Native Fruit Geoffroea decorticans from Arid Northern Chile: Phenolic Composition, Antioxidant Activities and In Vitro Inhibition of Pro-Inflammatory and Metabolic Syndrome-Associated Enzymes. *Molecules* 22. doi: 10.3390/molecules22091565

Jiménez-Aspee, F., Thomas-Valdés, S., Schulz, A., Ladio, A., Theoduloz, C., and Schmeda-Hirschmann, G. (2016b). Antioxidant activity and phenolic profiles of the wild currant Ribes magellanicum from Chilean and Argentinean Patagonia. *Food Sci. Nutr.* 4, 595–610. doi: https://doi.org/10.1002/fsn3.323

Jofré, I., Cuevas, M., de Castro, L. S., de Agostini Losano, J. D., Torres, M. A., Alvear, M., et al. (2019). Antioxidant Effect of a Polyphenol-Rich Murtilla (*Ugni molinae* Turcz.) Extract and Its Effect on the Regulation of Metabolism in Refrigerated Boar Sperm. *Oxid. Med. Cell. Longev.* 2019, 2917513. doi: 10.1155/2019/2917513

Jofré, I., Pezoa, C., Cuevas, M., Scheuermann, E., Freires, I. A., Rosalen, P. L., et al. (2016). Antioxidant and Vasodilator Activity of *Ugni molinae* Turcz. (Murtilla) and Its Modulatory Mechanism in Hypotensive Response. *Oxid. Med. Cell. Longev.* 2016, 6513416. doi: 10.1155/2016/6513416

Junqueira-Gonçalves, M. P., Yáñez, L., Morales, C., Navarro, M., Contreras, R. A., and Zúñiga, G. E. (2015). Isolation and Characterization of Phenolic Compounds and Anthocyanins from Murta (Ugni molinae Turcz.) Fruits. Assessment of Antioxidant and Antibacterial Activity. *Molecules* 20, 5698–5713. doi: 10.3390/molecules20045698

Kim, M. J., Kim, D. W., Kim, J. G., Shin, Y., Jung, S. K., and Kim, Y.-J. (2021). Analysis of the Chemical, Antioxidant, and Anti-Inflammatory Properties of Pink Pepper (Schinus molle L.). *Antioxidants* 10. doi: 10.3390/antiox10071062

Li, J., Yuan, C., Pan, L., Benatrehina, P. A., Chai, H., Keller, W. J., et al. (2017). Bioassay-Guided Isolation of Antioxidant and Cytoprotective Constituents from a Maqui Berry (Aristotelia chilensis) Dietary Supplement Ingredient As Markers for Qualitative and Quantitative Analysis. *J. Agric. Food Chem.* 65, 8634–8642. doi: 10.1021/acs.jafc.7b03261

López de Dicastillo, C., Bustos, F., Valenzuela, X., López-Carballo, G., Vilariño, J. M., and Galotto, M. J. (2017). Chilean berry Ugni molinae Turcz. fruit and leaves extracts with interesting antioxidant, antimicrobial and tyrosinase inhibitory properties. *Food Res. Int.* 102, 119–128. doi: https://doi.org/10.1016/j.foodres.2017.09.073

López, J., Shun Ah-Hen, K., Vega-Gálvez, A., Morales, A., García-Segovia, P., and Uribe, E. (2017a). Effects of drying methods on quality attributes of murta (Ugni molinae turcz) berries: bioactivity, nutritional aspects, texture profile, microstructure and functional properties. *J. Food Process Eng.* 40, e12511. doi: https://doi.org/10.1111/jfpe.12511

López, J., Vega-Gálvez, A., Bilbao-Sainz, C., Chiou, B.-S., Uribe, E., and Quispe-Fuentes, I. (2017b). Influence of vacuum drying temperature on: Physico-chemical composition and antioxidant properties of murta berries. *J. Food Process Eng.* 40, e12569. doi: https://doi.org/10.1111/jfpe.12569

López, J., Vega-Gálvez, A., Rodríguez, A., Stucken, K., Barraza, C., and Aguilera, L. E. (2019). Relationship between antimicrobial activity, phenolic profile and antioxidant capacity of murta ( Ugni molinae Turcz) extracts prepared by different drying methods. *J. Berry Res.* 9, 587–601. doi: 10.3233/JBR-190403

López, M. D., Baenas, N., Retamal-Salgado, J., Zapata, N., and Moreno, D. A. (2018). Underutilized Native Biobío Berries: Opportunities for Foods and Trade. *Nat. Prod. Commun.* 13, 1934578X1801301226. doi: 10.1177/1934578X1801301226

Lucas-Gonzalez, R., Navarro-Coves, S., Pérez-Álvarez, J. A., Fernández-López, J., Muñoz, L. A., and Viuda-Martos, M. (2016). Assessment of polyphenolic profile stability and changes in the antioxidant potential of maqui berry (Aristotelia chilensis (Molina) Stuntz) during in vitro gastrointestinal digestion. *Ind. Crops Prod.* 94, 774–782. doi: https://doi.org/10.1016/j.indcrop.2016.09.057

Madrid, A., Espinoza, L., Mellado, M., Montenegro, I., Gonzalez, C., Santander, R., et al. (2013). Study of the chemical composition of the resinous exudate isolated from Psoralea glandulosa and evaluation of the antioxidant properties of the terpenoids and the resin. *Boletín Latinoam. y del Caribe Plantas Med. y Aromáticas* 12, 338–345.

Martins, M. do R., Arantes, S., Candeias, F., Tinoco, M. T., and Cruz-Morais, J. (2014). Antioxidant, antimicrobial and toxicological properties of Schinus molle L. essential oils. *J. Ethnopharmacol.* 151, 485–492. doi: https://doi.org/10.1016/j.jep.2013.10.063

Mena, J., Elgueta, E., Espinola-Gonzales, F., Cardenas, H., and Orihuela, P. A. (2021). Hydroethanolic Extracts of the Aristotelia Chilensis (Maqui) Berry Reduces Cellular Viability and Invasiveness in the Endometrial Cancer Cell Line Ishikawa. *Integr. Cancer Ther.* 20, 15347354211007560. doi: 10.1177/15347354211007560

Mieres-Castro, D., Schmeda-Hirschmann, G., Theoduloz, C., Gómez-Alonso, S., Pérez-Navarro, J., Márquez, K., et al. (2019). Antioxidant activity and the isolation of polyphenols and new iridoids from Chilean Gaultheria phillyreifolia and G. poeppigii berries. *Food Chem.* 291, 167–179. doi: https://doi.org/10.1016/j.foodchem.2019.04.019

Mieres-Castro, D., Theoduloz, C., Sus, N., Burgos-Edwards, A., Schmeda-Hirschmann, G., Frank, J., et al. (2022). Iridoids and polyphenols from chilean Gaultheria spp. berries decrease the glucose uptake in Caco-2 cells after simulated gastrointestinal digestion. *Food Chem.* 369, 130940. doi: https://doi.org/10.1016/j.foodchem.2021.130940

Miranda-Rottmann, S., Aspillaga, A. A., Pérez, D. D., Vasquez, L., Martinez, A. L. F., and Leighton, F. (2002). Juice and Phenolic Fractions of the Berry Aristotelia chilensis Inhibit LDL Oxidation in Vitro and Protect Human Endothelial Cells against Oxidative Stress. *J. Agric. Food Chem.* 50, 7542–7547. doi: 10.1021/jf025797n

Moon, H.-D., and Kim, B.-H. (2020). Inhibitory effects of Aristotelia chilensis water extract on 2, 4-Dinitrochlorobenzene induced atopic-like dermatitis in BALB/c Mice. *Asian Pacific J. Allergy Immunol.* 38, 190–199.

Moscoso-Mujica, G., Mujica, Á., Chávez, J., Peña, C., Begazo, N., Estrella, J., et al. (2022). Antianemic activity of quinoa (Chenopodium quinoa Willd) Collana Negra variety and kanihua (Chenopodium pallidicaule Aellen) Ramis variety seed flour in anemic rats. *SN Appl. Sci.* 4, 318. doi: 10.1007/s42452-022-05202-w

Nakamura, S., Tanaka, J., Imada, T., Shimoda, H., and Tsubota, K. (2014). Delphinidin 3,5-O-diglucoside, a constituent of the maqui berry (Aristotelia chilensis) anthocyanin, restores tear secretion in a rat dry eye model. *J. Funct. Foods* 10, 346–354. doi: https://doi.org/10.1016/j.jff.2014.06.027

Noriega, F., Mardones, C., Fischer, S., García-Viguera, C., Moreno, D. A., and López, M. D. (2021). Seasonal changes in white strawberry: Effect on aroma, phenolic compounds and its biological activity. *J. Berry Res.* 11, 103–118. doi: 10.3233/JBR-200585

Nowak, D., Gośliński, M., Przygoński, K., and Wojtowicz, E. (2018). The antioxidant properties of exotic fruit juices from acai, maqui berry and noni berries. *Eur. Food Res. Technol.* 244, 1897–1905. doi: 10.1007/s00217-018-3102-8

Olivares-Caro, L., Radojkovic, C., Chau, S. Y., Nova, D., Bustamante, L., Neira, J. Y., et al. (2020). Berberis microphylla G. Forst (Calafate) Berry Extract Reduces Oxidative Stress and Lipid Peroxidation of Human LDL. *Antioxidants* 9. doi: 10.3390/antiox9121171

Ono, M., Yamashita, M., Mori, K., Masuoka, C., Eto, M., Kinjo, J., et al. (2008). Sesquiterpenoids, Triterpenoids, and Flavonoids from the Fruits of Schinus molle. *Food Sci. Technol. Res.* 14, 499. doi: 10.3136/fstr.14.499

Osman, E. E. A., Morsi, E. A., El-Sayed, M. M., Gobouri, A., and Abdel-Hameed, E. S. S. (2021). Identification of the volatile and nonvolatile constituents of Schinus molle (L.) fruit extracts and estimation of their activities as anticancer agents. *J. Appl. Pharm. Sci.* 11, 163–171. doi: 10.7324/JAPS.2021.110719

Otero, C., Miranda-Rojas, S., Llancalahuén, F. M., Fuentes, J. A., Atala, C., González-Silva, G., et al. (2022). Biochemical characterization of Peumus boldus fruits: Insights of its antioxidant properties through a theoretical approach. *Food Chem.* 370, 131012. doi: https://doi.org/10.1016/j.foodchem.2021.131012

Ovalle-Marin, A., Reyes-Farias, M., Vasquez, K., Parra-Ruiz, C., Quitral, V., Jimenez, P., et al. (2020). Maqui, Calafate, and Blueberry fruits extracts treatments suppress the pathogenic interaction amongst human adipocytes and macrophages. *J. Berry Res.* 10, 531–545. doi: 10.3233/JBR-200576

Oyarzún, P., Cornejo, P., Gómez-Alonso, S., and Ruiz, A. (2020). Influence of Profiles and Concentrations of Phenolic Compounds in the Coloration and Antioxidant Properties of Gaultheria poeppigii Fruits from Southern Chile. *Plant Foods Hum. Nutr.* 75, 532–539. doi: 10.1007/s11130-020-00843-x

Peçanha, J. de S., Santos, N. M. dos, Maróstica Júnior, M. R., Micheletti, A. C., Lião, L. M., and Alcantara, G. B. (2022). NMR-based metabolomics of dried berries in comparison with dietary supplements. *J. Pharm. Biomed. Anal.* 209, 114494. doi: https://doi.org/10.1016/j.jpba.2021.114494

Pellarín, M. G., Albrecht, C., Rojas, M. J., Aguilar, J. J., Konigheim, B. S., Paraje, M. G., et al. (2013). Inhibition of Cytotoxicity of Shiga Toxin of Escherichia coli O157:H7 on Vero Cells by Prosopis alba Griseb (Fabaceae) and Ziziphus mistol Griseb (Rhamnaceae) Extracts. *J. Food Prot.* 76, 1733–1739. doi: https://doi.org/10.4315/0362-028X.JFP-13-087

Peñarrieta, J. M., Alvarado, J. A., Åkesson, B., and Bergenståhl, B. (2008). Total antioxidant capacity and content of flavonoids and other phenolic compounds in canihua (Chenopodium pallidicaule): An Andean pseudocereal. *Mol. Nutr. Food Res.* 52, 708–717. doi: https://doi.org/10.1002/mnfr.200700189

Pérez-Arancibia, R., Ordoñez, J. L., Rivas, A., Pihán, P., Sagredo, A., Ahumada, U., et al. (2021). A phenolic-rich extract from Ugni molinae berries reduces abnormal protein aggregation in a cellular model of Huntington’s disease. *PLoS One* 16, e0254834. Available at: https://doi.org/10.1371/journal.pone.0254834

Pérez-López, A., Cirio, A. T., Rivas-Galindo, V. M., Aranda, R. S., and de Torres, N. W. (2011). Activity against Streptococcus pneumoniae of the Essential Oil and δ-Cadinene Isolated from Schinus molle Fruit. *J. Essent. Oil Res.* 23, 25–28. doi: 10.1080/10412905.2011.9700477

Pérez, M. J., Cuello, A. S., Zampini, I. C., Ordoñez, R. M., Alberto, M. R., Quispe, C., et al. (2014). Polyphenolic compounds and anthocyanin content of Prosopis nigra and Prosopis alba pods flour and their antioxidant and anti-inflammatory capacities. *Food Res. Int.* 64, 762–771. doi: https://doi.org/10.1016/j.foodres.2014.08.013

Pineda, A., Arenas, A., Balmaceda, J., and Zúñiga, G. E. (2022). Extracts of Fruits and Plants Cultivated In Vitro of Aristotelia chilensis (Mol.) Stuntz Show Inhibitory Activity of Aldose Reductase and Pancreatic Alpha-Amylase Enzymes. *Plants* 11. doi: 10.3390/plants11202772

Pinto-Morales, F., Retamal-Salgado, J., López, M. D., Zapata, N., Vergara-Retamales, R., and Palma, D. (2022). Variation in Physical-Chemical Parameters and Phenolic Compounds in Fruits of Four Calafate Clones. *Agronomy* 12. doi: 10.3390/agronomy12092146

Pinto, A. A., Fuentealba-Sandoval, V., López, M. D., Peña-Rojas, K., and Fischer, S. (2022). Accumulation of delphinidin derivatives and other bioactive compound in wild maqui under different environmental conditions and fruit ripening stages. *Ind. Crops Prod.* 184, 115064. doi: https://doi.org/10.1016/j.indcrop.2022.115064

Quispe-Fuentes, I., Vega-Gálvez, A., and Aranda, M. (2018). Evaluation of phenolic profiles and antioxidant capacity of maqui (Aristotelia chilensis) berries and their relationships to drying methods. *J. Sci. Food Agric.* 98, 4168–4176. doi: https://doi.org/10.1002/jsfa.8938

Quispe-Fuentes, I., Vega-Gálvez, A., Aranda, M., Poblete, J., Pasten, A., Bilbao-Sainz, C., et al. (2020). Effects of drying processes on composition, microstructure and health aspects from maqui berries. *J. Food Sci. Technol.* 57, 2241–2250. doi: 10.1007/s13197-020-04260-5

Quispe-Fuentes, I., Vega-Gálvez, A., Uribe, E., Vásquez, V., Cárdenas, N., and Poblete, J. (2019). Vacuum drying application to maqui (Aristotelia chilensis [Mol] Stuntz) berry: Weibull distribution for process modelling and quality parameters. *J. Food Sci. Technol.* 56, 1899–1908. doi: 10.1007/s13197-019-03653-5

Ramirez, J. E., Zambrano, R., Sepúlveda, B., Kennelly, E. J., and Simirgiotis, M. J. (2015). Anthocyanins and antioxidant capacities of six Chilean berries by HPLC–HR-ESI-ToF-MS. *Food Chem.* 176, 106–114. doi: https://doi.org/10.1016/j.foodchem.2014.12.039

Reyes-Farias, M., Vasquez, K., Fuentes, F., Ovalle-Marin, A., Parra-Ruiz, C., Zamora, O., et al. (2016). Extracts of Chilean native fruits inhibit oxidative stress, inflammation and insulin-resistance linked to the pathogenic interaction between adipocytes and macrophages. *J. Funct. Foods* 27, 69–83. doi: https://doi.org/10.1016/j.jff.2016.08.052

Reyes-Farias, M., Vasquez, K., Ovalle-Marin, A., Fuentes, F., Parra, C., Quitral, V., et al. (2014). Chilean Native Fruit Extracts Inhibit Inflammation Linked to the Pathogenic Interaction Between Adipocytes and Macrophages. *J. Med. Food* 18, 601–608. doi: 10.1089/jmf.2014.0031

Reynoso, M. A., Vera, N., Aristimuño, M. E., Daud, A., and Sánchez Riera, A. (2013). Antinociceptive activity of fruits extracts and “arrope” of Geoffroea decorticans (chañar). *J. Ethnopharmacol.* 145, 355–362. doi: https://doi.org/10.1016/j.jep.2012.11.022

Rocha, P. M. de M., Rodilla, J. M., Díez, D., Elder, H., Guala, M. S., Silva, L. A., et al. (2012). Synergistic Antibacterial Activity of the Essential Oil of Aguaribay (Schinus molle L.). *Molecules* 17, 12023–12036. doi: 10.3390/molecules171012023

Rodríguez, I. F., Cattaneo, F., Zech, X. V., Svavh, E., Pérez, M. J., Zampini, I. C., et al. (2020). Aloja and añapa, two traditional beverages obtained from Prosopis alba pods: Nutritional and functional characterization. *Food Biosci.* 35, 100546. doi: https://doi.org/10.1016/j.fbio.2020.100546

Rodriguez, I. F., Pérez, M. J., Cattaneo, F., Zampini, I. C., Cuello, A. S., Mercado, M. I., et al. (2019). Morphological, histological, chemical and functional characterization of Prosopis alba flours of different particle sizes. *Food Chem.* 274, 583–591. doi: https://doi.org/10.1016/j.foodchem.2018.09.024

Rodríguez, K., Ah-Hen, K. S., Vega-Gálvez, A., Vásquez, V., Quispe-Fuentes, I., Rojas, P., et al. (2016). Changes in bioactive components and antioxidant capacity of maqui, Aristotelia chilensis [Mol] Stuntz, berries during drying. *LWT - Food Sci. Technol.* 65, 537–542. doi: https://doi.org/10.1016/j.lwt.2015.08.050

Rodríguez, K., Ah-Hen, K., Vega-Gálvez, A., López, J., Quispe-Fuentes, I., Lemus-Mondaca, R., et al. (2014). Changes in bioactive compounds and antioxidant activity during convective drying of murta (Ugni molinae T.) berries. *Int. J. Food Sci. Technol.* 49, 990–1000. doi: https://doi.org/10.1111/ijfs.12392

Rodríguez, L., Trostchansky, A., Wood, I., Mastrogiovanni, M., Vogel, H., González, B., et al. (2021). Antiplatelet activity and chemical analysis of leaf and fruit extracts from Aristotelia chilensis. *PLoS One* 16, e0250852. Available at: https://doi.org/10.1371/journal.pone.0250852

Rojo, L. E., Ribnicky, D., Logendra, S., Poulev, A., Rojas-Silva, P., Kuhn, P., et al. (2012). In vitro and in vivo anti-diabetic effects of anthocyanins from Maqui Berry (Aristotelia chilensis). *Food Chem.* 131, 387–396. doi: https://doi.org/10.1016/j.foodchem.2011.08.066

Roldán, C. S., Caballé, G., Fontana, A., Viale, M., and Berli, F. (2021). Maqui (Aristotelia chilensis [Mol.] Stuntz) morphological and phenolic traits associated with forests type and latitudinal gradient in natural populations of Patagonia Argentina. *J. Appl. Res. Med. Aromat. Plants* 25, 100341. doi: https://doi.org/10.1016/j.jarmap.2021.100341

Romero-Román, M. E., Schoebitz, M., Bastías, R. M., Fernández, P. S., García-Viguera, C., and López-Belchi, M. D. (2021a). Native Species Facing Climate Changes: Response of Calafate Berries to Low Temperature and UV Radiation. *Foods* 10. doi: 10.3390/foods10010196

Romero-Román, M. E., Schoebitz, M., Fuentealba, J., García-Viguera, C., and Belchí, M. D. L. (2021b). Phenolic Compounds in Calafate Berries Encapsulated by Spray Drying: Neuroprotection Potential into the Ingredient. *Antioxidants* 10. doi: 10.3390/antiox10111830

Rubilar, M., Jara, C., Poo, Y., Acevedo, F., Gutierrez, C., Sineiro, J., et al. (2011). Extracts of Maqui (Aristotelia chilensis) and Murta (Ugni molinae Turcz.): Sources of Antioxidant Compounds and α-Glucosidase/α-Amylase Inhibitors. *J. Agric. Food Chem.* 59, 1630–1637. doi: 10.1021/jf103461k

Ruiz, A., Bustamante, L., Vergara, C., von Baer, D., Hermosín-Gutiérrez, I., Obando, L., et al. (2015). Hydroxycinnamic acids and flavonols in native edible berries of South Patagonia. *Food Chem.* 167, 84–90. doi: https://doi.org/10.1016/j.foodchem.2014.06.052

Ruiz, A., Hermosín-Gutiérrez, I., Mardones, C., Vergara, C., Herlitz, E., Vega, M., et al. (2010). Polyphenols and Antioxidant Activity of Calafate (Berberis microphylla) Fruits and Other Native Berries from Southern Chile. *J. Agric. Food Chem.* 58, 6081–6089. doi: 10.1021/jf100173x

Ruiz, A., Hermosín-Gutiérrez, I., Vergara, C., von Baer, D., Zapata, M., Hitschfeld, A., et al. (2013). Anthocyanin profiles in south Patagonian wild berries by HPLC-DAD-ESI-MS/MS. *Food Res. Int.* 51, 706–713. doi: https://doi.org/10.1016/j.foodres.2013.01.043

Sandoval, V., Femenias, A., Martínez-Garza, Ú., Sanz-Lamora, H., Castagnini, J. M., Quifer-Rada, P., et al. (2019). Lyophilized Maqui (Aristotelia chilensis) Berry Induces Browning in the Subcutaneous White Adipose Tissue and Ameliorates the Insulin Resistance in High Fat Diet-Induced Obese Mice. *Antioxidants* 8. doi: 10.3390/antiox8090360

Sandoval, V., Sanz-Lamora, H., Marrero, P. F., Relat, J., and Haro, D. (2021). Lyophilized Maqui (Aristotelia chilensis) Berry Administration Suppresses High-Fat Diet-Induced Liver Lipogenesis through the Induction of the Nuclear Corepressor SMILE. *Antioxidants* 10. doi: 10.3390/antiox10050637

Schmeda-Hirschmann, G., Antileo-Laurie, J., Theoduloz, C., Jiménez-Aspee, F., Avila, F., Burgos-Edwards, A., et al. (2021). Phenolic composition, antioxidant capacity and α-glucosidase inhibitory activity of raw and boiled Chilean Araucaria araucana kernels. *Food Chem.* 350, 129241. doi: https://doi.org/10.1016/j.foodchem.2021.129241

Schmeda-Hirschmann, G., Quispe, C., Soriano, M. D. P. C., Theoduloz, C., Jiménez-Aspée, F., Pérez, M. J., et al. (2015). Chilean Prosopis Mesocarp Flour: Phenolic Profiling and Antioxidant Activity. *Molecules* 20, 7017–7033. doi: 10.3390/molecules20047017

Schmeda-Hirschmann, G., Razmilic, I., Gutierrez, M. I., and Loyola, J. I. (1999). Proximate composition and biological activity of food plants gathered by chilean Amerindians. *Econ. Bot.* 53, 177–187. doi: 10.1007/BF02866496

Schreckinger, M. E., Wang, J., Yousef, G., Lila, M. A., and Gonzalez de Mejia, E. (2010). Antioxidant Capacity and in Vitro Inhibition of Adipogenesis and Inflammation by Phenolic Extracts of Vaccinium floribundum and Aristotelia chilensis. *J. Agric. Food Chem.* 58, 8966–8976. doi: 10.1021/jf100975m

Schreckinger, M., Lila, M. A., Yousef, G., and de Mejia, E. (2012). “Inhibition of α-Glucosidase and α-Amylase by Vaccinium floribundum and Aristotelia chilensis Proanthocyanidins,” in *Hispanic Foods: Chemistry and Bioactive Compounds*, (American Chemical Society), 6–71. doi: doi:10.1021/bk-2012-1109.ch006

Sciammaro, L. P., Quintero Ruiz, N. A., Ferrero, C., Giacomino, S., Picariello, G., Mamone, G., et al. (2021). Prosopis spp. powder: influence of chemical components in water adsorption properties. *Int. J. Food Sci. Technol.* 56, 278–286. doi: https://doi.org/10.1111/ijfs.14629

Shene, C., Reyes, A. K., Villarroel, M., Sineiro, J., Pinelo, M., and Rubilar, M. (2009). Plant location and extraction procedure strongly alter the antimicrobial activity of murta extracts. *Eur. Food Res. Technol.* 228, 467–475. doi: 10.1007/s00217-008-0954-3

Simirgiotis, M. J., Bórquez, J., and Schmeda-Hirschmann, G. (2013). Antioxidant capacity, polyphenolic content and tandem HPLC–DAD–ESI/MS profiling of phenolic compounds from the South American berries Luma apiculata and L. chequén. *Food Chem.* 139, 289–299. doi: https://doi.org/10.1016/j.foodchem.2013.01.089

Simirgiotis, M. J., and Schmeda-Hirschmann, G. (2010). Determination of phenolic composition and antioxidant activity in fruits, rhizomes and leaves of the white strawberry (Fragaria chiloensis spp. chiloensis form chiloensis) using HPLC-DAD–ESI-MS and free radical quenching techniques. *J. Food Compos. Anal.* 23, 545–553. doi: https://doi.org/10.1016/j.jfca.2009.08.020

Simirgiotis, M. J., Theoduloz, C., Caligari, P. D. S., and Schmeda-Hirschmann, G. (2009). Comparison of phenolic composition and antioxidant properties of two native Chilean and one domestic strawberry genotypes. *Food Chem.* 113, 377–385. doi: https://doi.org/10.1016/j.foodchem.2008.07.043

Somaini, G. C., Aybar, M. J., Vera, N. R., and Tríbulo, C. (2021). Geoffroea decorticans fruit extracts inhibit the wnt/β-catenin pathway, a therapeutic target in cancer. *Biochem. Biophys. Res. Commun.* 546, 118–123. doi: https://doi.org/10.1016/j.bbrc.2021.01.087

Soto-Covasich, J., Reyes-Farias, M., Torres, R. F., Vasquez, K., Duarte, L., Quezada, J., et al. (2020). A polyphenol-rich Calafate (Berberis microphylla) extract rescues glucose tolerance in mice fed with cafeteria diet. *J. Funct. Foods* 67, 103856. doi: https://doi.org/10.1016/j.jff.2020.103856

Suwalsky, M., and Avello, M. (2014). Antioxidant Capacity of Ugni molinae Fruit Extract on Human Erythrocytes: An In Vitro Study. *J. Membr. Biol.* 247, 703–712. doi: 10.1007/s00232-014-9692-6

Tanaka, J., Kadekaru, T., Ogawa, K., Hitoe, S., Shimoda, H., and Hara, H. (2013). Maqui berry (Aristotelia chilensis) and the constituent delphinidin glycoside inhibit photoreceptor cell death induced by visible light. *Food Chem.* 139, 129–137. doi: https://doi.org/10.1016/j.foodchem.2013.01.036

Tellez, M., Gianetti, A., Covarrubias, E., and Barzelatto, J. (1969). “Endemic goiter in Pedregoso (Chile). Experimental goitrogenic activity of ‘piñon’.,” in *Endemic Goiter.*, ed. J. B. Stanbury (World Health Organization).

Theoduloz, C., Burgos-Edwards, A., Schmeda-Hirschmann, G., and Jiménez-Aspee, F. (2018). Effect of polyphenols from wild Chilean currants (Ribes spp.) on the activity of intracellular antioxidant enzymes in human gastric AGS cells. *Food Biosci.* 24, 80–88. doi: https://doi.org/10.1016/j.fbio.2018.06.003

Thomas-Valdés, S., Theoduloz, C., Jiménez-Aspee, F., Burgos-Edwards, A., and Schmeda-Hirschmann, G. (2018). Changes in polyphenol composition and bioactivity of the native Chilean white strawberry (Fragaria chiloensis spp. chiloensis f. chiloensis) after in vitro gastrointestinal digestion. *Food Res. Int.* 105, 10–18. doi: https://doi.org/10.1016/j.foodres.2017.10.074

Thomas-Valdés, S., Theoduloz, C., Jiménez-Aspee, F., and Schmeda-Hirschmann, G. (2019). Effect of simulated gastrointestinal digestion on polyphenols and bioactivity of the native Chilean red strawberry (Fragaria chiloensis ssp. chiloensis f. patagonica). *Food Res. Int.* 123, 106–114. doi: https://doi.org/10.1016/j.foodres.2019.04.039

Tlili, N., Yahia, Y., Feriani, A., Labidi, A., Ghazouani, L., Nasri, N., et al. (2018). Schinus terebinthifolius vs Schinus molle: A comparative study of the effect of species and location on the phytochemical content of fruits. *Ind. Crops Prod.* 122, 559–565. doi: https://doi.org/10.1016/j.indcrop.2018.05.080

Velásquez, P., Orellana, J., Muñoz-Carvajal, E., Faúndez, M., Gómez, M., Montenegro, G., et al. (2022). Biological activity of native Myrtaceae fruits from Chile as a potential functional food. *Nat. Prod. Res.* 36, 3138–3142. doi: 10.1080/14786419.2021.1940176

Vergara, D., Ávila, D., Escobar, E., Carrasco-Pozo, C., Sánchez, A., and Gotteland, M. (2015). The intake of maqui (Aristotelia chilensis) berry extract normalizes H2O2 and IL-6 concentrations in exhaled breath condensate from healthy smokers - an explorative study. *Nutr. J.* 14, 27. doi: 10.1186/s12937-015-0008-1

Viktorová, J., Kumar, R., Řehořová, K., Hoang, L., Ruml, T., Figueroa, C. R., et al. (2020). Antimicrobial Activity of Extracts of Two Native Fruits of Chile: Arrayan (Luma apiculata) and Peumo (Cryptocarya alba). *Antibiotics* 9. doi: 10.3390/antibiotics9080444

Volpini-Klein, A. F. N., Silva, C. A. A., Fernandes, S. S. L., Nicolau, C. L., Cardoso, C. A. L., Fiorucci, A. R., et al. (2020). Effect of leaf and fruit extracts of Schinus molle on oxidative stability of some vegetables oils under accelerated oxidation. *Grasas y Aceites* 71, e363. doi: 10.3989/gya.0456191

Wang, J. Z., Yousef, G. G., Rogers, R. B., Gonzalez de Mejia, E., Raskin, I., and Lila, M. A. (2012). “Maqui Berry (Aristotelia chilensis) Juices Fermented with Yeasts: Effects on Phenolic Composition, Antioxidant Capacity, and iNOS and COX-2 Protein Expression,” in *Emerging Trends in Dietary Components for Preventing and Combating Disease*, (American Chemical Society), 6–95. doi: doi:10.1021/bk-2012-1093.ch006

Wang, S. Y., and Lewers, K. S. (2007). Antioxidant Capacity and Flavonoid Content in Wild Strawberries. *J. Am. Soc. Hortic. Sci. J. Amer. Soc. Hort. Sci.* 132, 629–637. doi: 10.21273/JASHS.132.5.629

Yueqin, Z., Recio, M. C., Máñez, S., Giner, R. M., Cerdá-Nicolás, M., and Ríos, J.-L. (2003). Isolation of Two Triterpenoids and a Biflavanone with Anti-Inflammatory Activity from Schinus molle Fruits. *Planta Med* 69, 893–898. doi: 10.1055/s-2003-45096

Zambrana, S., Mamani, O., Canaviri, M., Gutiérrez, M. del P., Sergiu-Bogdan, C., Claes-Goran, O., et al. (2020). Glycemia-reducing effects of Bolivian nutraceutical plants. *Ars Pharm.* 62, 52–65.

Zhou, G., Chen, L., Sun, Q., Mo, Q.-G., Sun, W.-C., and Wang, Y.-W. (2019). Maqui berry exhibited therapeutic effects against DSS-induced ulcerative colitis in C57BL/6 mice. *Food Funct.* 10, 6655–6665. doi: 10.1039/C9FO00663J
